# New Multi-Targeted Antiproliferative Agents: Design and Synthesis of IC261-Based Oxindoles as Potential Tubulin, CK1 and EGFR Inhibitors

**DOI:** 10.3390/ph14111114

**Published:** 2021-10-30

**Authors:** Momen R. Fareed, Mai E. Shoman, Mohammed I. A. Hamed, Mohamed Badr, Hanin A. Bogari, Sameh S. Elhady, Tarek S. Ibrahim, Gamal El-Din A. Abuo-Rahma, Taha F. S. Ali

**Affiliations:** 1 Department of Medicinal Chemistry, Faculty of Pharmacy, Minia University, Minia 61519, Egypt; Momen.ramadan@mu.edu.eg (M.R.F.); tahafaroukali@gmail.com (T.F.S.A.); gamal.aborahma@mu.edu.eg (G.E.-D.A.A.-R.); 2 Department of Organic and Medicinal Chemistry, Faculty of Pharmacy, Fayoum University, Fayoum 63514, Egypt; mia06@fayoum.edu.eg; 3 Department of Biochemistry, Faculty of Pharmacy, Menoufia University, Shibin el Kom 32511, Egypt; mohamed.badr@phrm.menofia.edu.eg; 4 Department of Pharmacy Practice, Faculty of Pharmacy, King Abdulaziz University, Jeddah 21589, Saudi Arabia; hbogari@kau.edu.sa; 5 Department of Natural Products, Faculty of Pharmacy, King Abdulaziz University, Jeddah 21589, Saudi Arabia; ssahmed@kau.edu.sa; 6 Department of Pharmaceutical Chemistry, Faculty of Pharmacy, King Abdulaziz University, Jeddah 21589, Saudi Arabia; 7 Department of Pharmaceutical Organic Chemistry, Faculty of Pharmacy, Zagazig University, Zagazig 44519, Egypt; 8 Department of Pharmaceutical Medicinal Chemistry, Faculty of Pharmacy, Deraya University, New Minia 61111, Egypt

**Keywords:** oindoles, antiproliferative, tubulin polymerization inhibitors, EGFR kinase inhibitors, casein kinase

## Abstract

A series of 3-benzylideneindolin-2-one compounds was designed and synthesized based on combretastatin A-4 and compound **IC261**, a dual casein kinase (CK1)/tubulin polymerization inhibitor, taking into consideration the pharmacophore required for EGFR-tyrosine kinase inhibition. The new molecular entities provoked significant growth inhibition against PC-3, MCF-7 and COLO-205 at a 10 μM dose. Compounds 6-chloro-3-(2,4,6-trimethoxybenzylidene) indolin-2-one, **4b,** and 5-methoxy-3-(2,4,6-trimethoxybenzylidene)indolin-2-one, **4e,** showed potent activity against the colon cancer COLO-205 cell line with an IC_50_ value of 0.2 and 0.3 μM. A mechanistic study demonstrated **4b**’s efficacy in inhibiting microtubule assembly (IC_50_ = 1.66 ± 0.08 μM) with potential binding to the colchicine binding site (docking study). With an IC_50_ of 1.92 ± 0.09 μg/mL, **4b** inhibited CK1 almost as well as **IC261**. Additionally, **4b** and **4e** were effective inhibitors of EGFR-TK with IC_50_s of 0.19 μg/mL and 0.40 μg/mL compared to Gifitinib (IC_50_ = 0.05 μg/mL). Apoptosis was induced in COLO-205 cells treated with **4b,** with apoptotic markers dysregulated. Caspase 3 levels were elevated to more than three-fold, while Cytochrome C levels were doubled. The cell cycle was arrested in the pre-G1 phase with extensive cellular accumulation in the pre-G1 phase, confirming apoptosis induction. Levels of cell cycle regulating proteins BAX and Bcl-2 were also defective. The binding interaction patterns of these compounds at the colchicine binding site of tubulin and the Gifitinib binding site of EGFR were verified by molecular docking, which adequately matched the reported experimental result. Hence, **4b** and **4e** are considered promising potent multitarget agents against colon cancer that require optimization.

## 1. Introduction

Nowadays, cancer is considered to be the second leading cause of death after cardiovascular diseases [1,2,3]. According to the World Health Organization, around 14 million new cases and 9 million new deaths occur per year [4]. Moreover, alarming predictions claim that cancer deaths may reach 13 million per year by 2030 [5]. So, many efforts are directed to defeat this disease. Several anticancer agents have been developed to encounter this rapid, uncontrolled, and pathological proliferation of abnormal cells, but many barriers are still present and need to be overcome. Obstacles such as high toxicity, low efficacy, high cost, and acquired drug resistance have a great impact on the normal life of patients [6]. Consequently, designing novel potent, selective and safe anticancer compounds is a major challenge in modern cancer research [7].

The indole scaffold is a critical motif in drug design and incorporated in 5–10% of currently known drugs. It is also one of the most important frameworks with considerable anticancer activities and frequently presents in active compounds and natural products. Mechanisms by which indole derivatives exhibit their anticancer activity include inhibition of microtubules [8,9], protein kinase [7,10], DNA topoisomerase [11] and histone deacetylase, in addition to induction of apoptosis [12].

Microtubules, built from a basic α/β-tubulin building block, are components of the cellular cytoskeleton shaped by the dynamic assembly of tubulin heterodimers. In view of its key role in cell growth and function, the microtubule system of eukaryotic cells is a substantially important molecular target for cancer chemotherapeutic agents [13].

**Figure 1:**
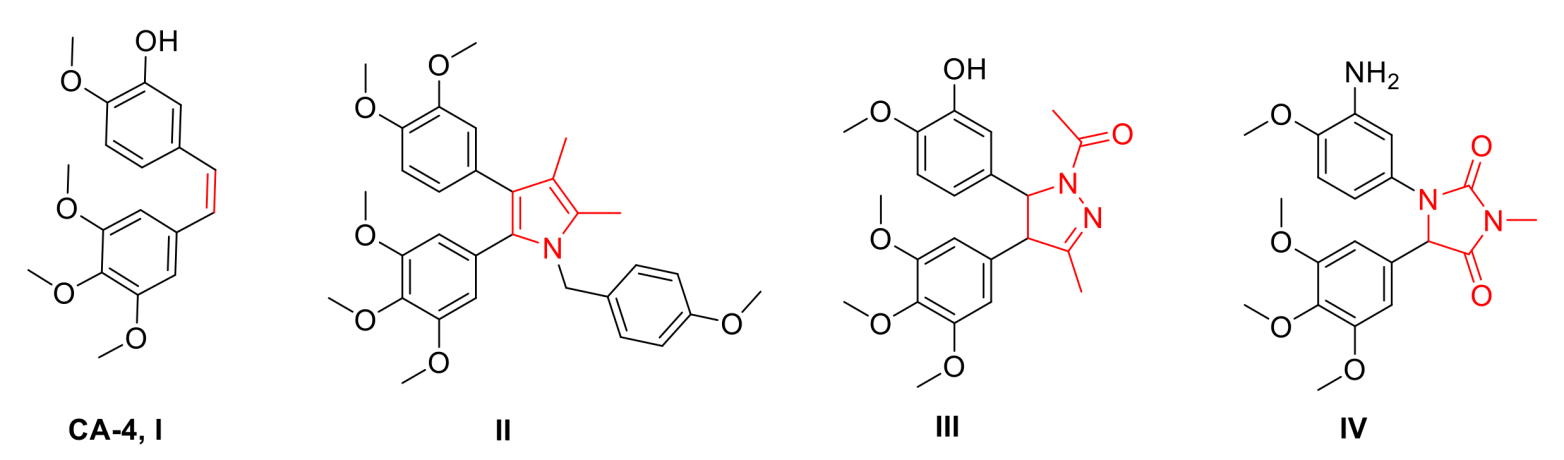
Chemical structure of CA-4, (**I**) and its analogs (**II**–**IV**).

**Figure 2:**
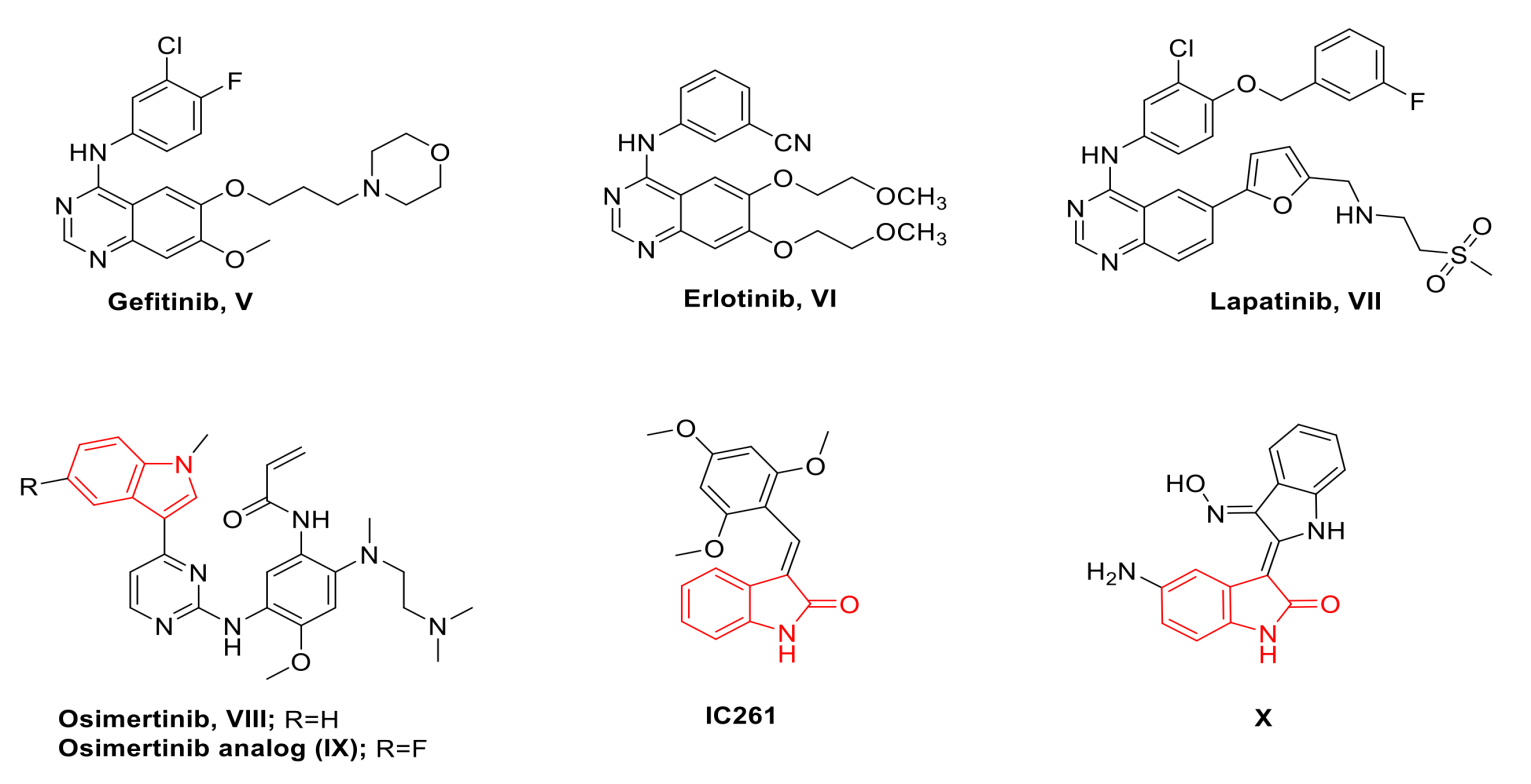
Structures of EGFR and CK1 inhibitors as potent anticancer agents.

The disturbance of microtubule dynamics exposes cells to metaphase arrest and mitotic catastrophe, and this obstruction was found to be valuable for developing novel anticancer agents, such as vinca alkaloids (vinblastine and vincristine) and taxanes (paclitaxel and docetaxel) [8,14]. One of the foremost important tubulin inhibitors is combretastatin A-4 (CA-4), which is derived from the bark of the South African tree Combretum Caffrum [15,16] (Figure 1). CA-4 is a cis-stilbenoid phenol that exhibits significant inhibition of tubulin polymerization upon binding to the colchicine active site, as well as remarkable in vitro cytotoxicity against various cancer cell lines. In addition, CA-4 destroys the vascular endothelial cadherin leading to ischemic necrosis of the tumor cells [15,17,18]. Due to the spontaneous isomerization of the olefinic bond from the cis-isomer to the more stable trans- isomer, the in vivo antitubulin and cytotoxic activities of CA-4 are significantly reduced [19]. Consequently, much research has been carried out to restrict the cis-orientation of the ethylene bond by replacing it with rigid heterocyclic rings such as pyrrole (II, Figure 1) [20], imidazole (IV, Figure 1) [21], pyrazoline (III, Figure 1) [22], oxadiazole [23], triazoles [24], pyridine [25], pyrimidine [26], β-lactam [27] and hydantoin [28]. The indole ring was used for the same purpose in compound **IC261**, Figure 2.

Another target for designing indole derivatives as anticancer agents is the family of protein kinase receptors. The epidermal growth factor receptor (EGFR) is a transmembrane glycoprotein that is one of four members of the ErbB family of tyrosine kinase receptors [29]. Activation of EGFR prompts autophosphorylation of the receptor tyrosine kinase, thereby initiating a series of downstream signaling pathways that are involved in regulating cellular proliferation, differentiation, and survival. EGFR is abnormally activated through various mechanisms, such as receptor overexpression, mutation, ligand-dependent receptor dimerization, and ligand-independent activation, and is associated with the development of a variety of human cancers, so EGFR inhibition is one of the key objectives for cancer chemotherapy [30]. Approved tyrosine kinase inhibitors such as gefitinib, erlotinib and lapatinib (V–VII, Figure 2) for the treatment of non-small cell lung cancer have led to tremendous development of novel EGFR inhibitors in the last decade [30]. Osimertinib (VIII, Figure 2) is an FDA-approved indole-containing drug to treat NSCLC as an EGFR inhibitor; however, it exhibits severe adverse effects and cardiotoxicity at high doses and is of low selectivity towards EGFR [31]. To increase the selectivity to EGFR, Zaho et al., studied the SAR of osimertinib, VIII [32]. They concluded that fluorinated analogs showed increased selectivity toward EGFR^T790M/L858R^ double mutants (IC_50_ = 8 nM) and about a 200-fold improvement in selectivity against wild type EGFR (Figure 2). Thus, it was supposed that the fluorinated indole (IX) has a crucial role in improving the binding affinity to the T790M active binding domain of EGFR and stabilizing the indole ring against demethylation, which can protect osimertinib derivatives against metabolism [9].

Moreover, casein kinase 1 (CK1) is a family of ubiquitous serine/threonine protein kinases containing seven family members α, β, γ1, γ2, γ3, δ and ε [33]. Numerous studies have reported that CK1 kinase can regulate various cellular biological behaviors, including DNA replication, differentiation, proliferation, membrane transport signal transduction and apoptosis [34,35]. Based on these biological regulatory effects, it is not surprising that carcinogenesis involves changes and mutations in CK1 expression [36]. Some small molecule have been synthesized and clinically targeted drugs have been developed as CK1 inhibitors [37]. Among these inhibitors, **IC261** (Figure 2), which targets CK1δ and CK1ε [8,38], has been the most widely studied. In addition, 5-amino-3-N-oxime-indirubin derivative X exhibited inhibitory activity against CK1 in the submicromolar range of 0.13 μM [39].

### Rationale of Design

One of the most significant drug design strategies used to decrease drug toxicity and advance biological activity is the multitarget directed ligand (MTDL) technique, which involves the covalent conjugation of various active moieties (pharmacophores) with diverse mechanisms of action, yet with particular pharmacophoric collection for each individual target [40]. Founded on this concept, the design of our recently recommended multitubulin/protein kinase inhibitors were based on maintaining the essential pharmacophores to hit the targets concomitantly while keeping good pharmacodynamic and pharmacokinetic profiles. Based on the affectivity and importance of the indole skeleton in design and development of anticancer drugs, the authors overviewed the synthesis of indole-based derivatives, aiming to develop new small molecules that are potentially capable of interfering with tubulin polymerization and protein kinase (EGFR and CK1) receptors, leading to potential anticancer activity.

**Figure 3:**
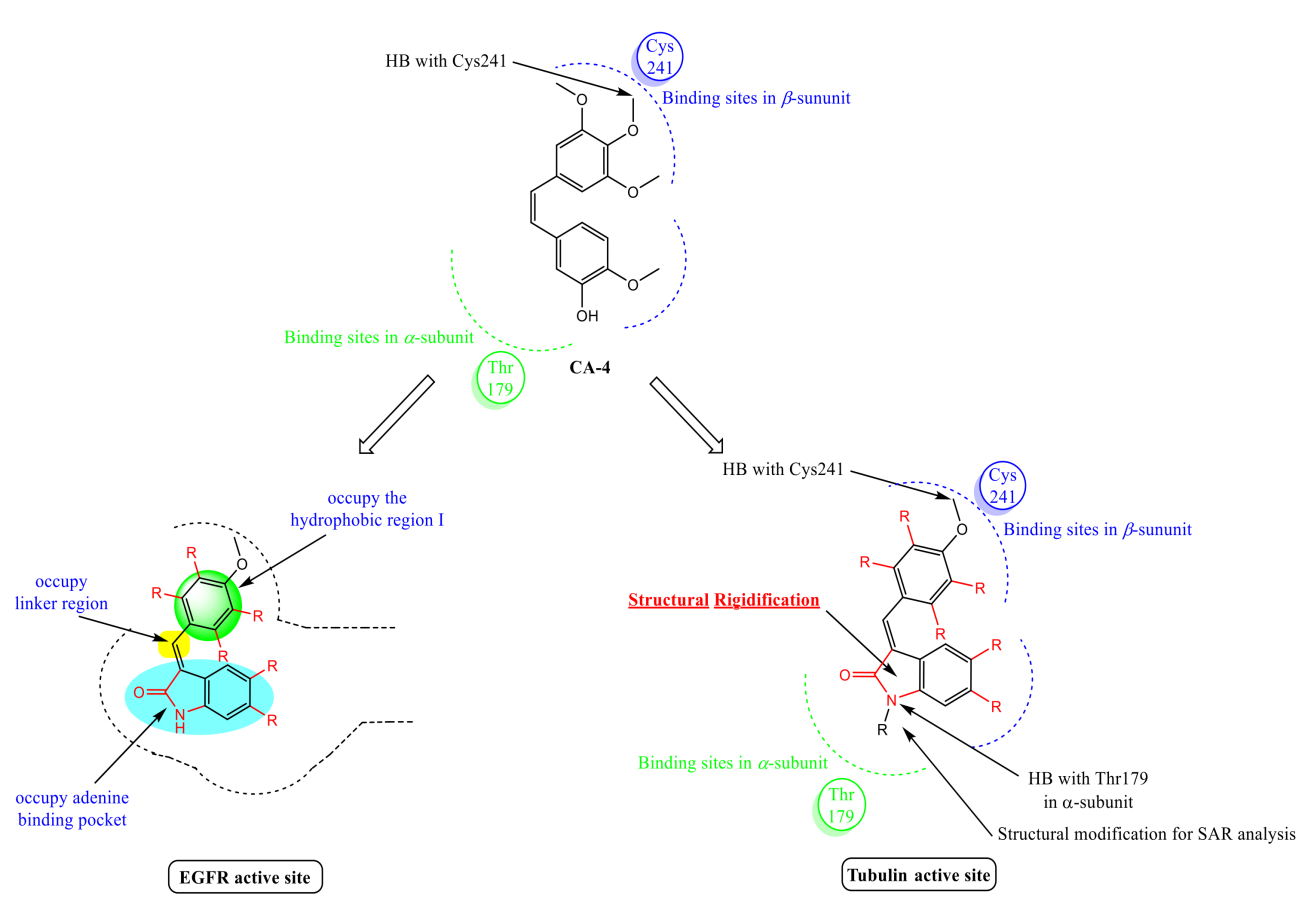
Design of 3-benzylideneindolin-2-one derivatives as tubulin polymerization and protein kinase inhibitors, and their potential binding to tubulin and EGFR kinase active sites. Dotted lines represent active site outline while solid arrows refer to the design flow.

The discovery of compounds that act on the colchicine binding site of β-tubulin has aroused great interest because these compounds selectively disrupt the vasculature of tumors without damaging normal blood vessels. Considering the potent cytotoxic activity of CA-4 through tubulin polymerization inhibition, novel 3-benzylideneindolin-2-one derivatives were designed to interact with both α and β-subunits of tubulin (Figure 3). The SAR study of CA-4 has three fundamental features [24,41]. (A) the presence of a trimethoxy group in ring A is generally critical for the activity. Subsequently, it was fixed for all the synthesized compounds either 3,5,6-trimethoxy or 2,4,6-trimethoxy. (B) It was observed that a drastic decrease in the antitubulin and cytotoxic activity of an in vivo CA-4 inhibitory assay occurred due to the spontaneous isomerization of the olefinic bond from cis-isomer to the more stable trans-isomer, so the present work involved restriction of the cis-orientation for the ethylene bond through structural rigidification (cyclization) with a rigid indole heterocyclic ring and, in the same way, facilitated the interaction with the key amino acid Thr179 in the α-subunit. (C) Ring B showed a successful bioisosteric replacement of the m-hydroxy group by a chlorine group. The p-methoxy group of most analogs was maintained or replaced by the electron-withdrawing group either fluorine or chlorine. Searching for more bindings inside the colchicine active site, the indolin-2-one ring substituted at position 1 with p-substituted benzyl group provided an extra-binding site, which in turn permitted more interactions inside the pocket.

Other drug targets for designing 3-benzylideneindolin-2-one derivatives anticancer agents are protein kinase (EGFR and CK1) receptors. These newly synthesized compounds were designed to accommodate the EGFR binding site through bioisosteric replacement of quinazoline moiety in gefitinib by indolin-2-one moiety to interact by hydrogen bonding with the key amino acid Met793. The 3-chloro-4-flurophenyl group of the anticancer drug gefitinib replaced by trimethoxybenzylidene group exhibited different interactions within the hydrophobic pocket. The interactions of the newly synthesized compounds inside the targets binding sites are described in detail in the molecular docking study in Section 2.3. Concerning the CK1 receptor, optimization was carried out for the lead compound **IC261** (**4a**) to improve its metabolic resistance through blocking positions 5 and 6 at indolin-2-one moiety by diverse substituents (F, Cl and OCH_3_).

In the present work, a new series of 3-benzylidenindolin-2-one derivatives were designed and synthesized, as shown in Figure 3. All the synthesized analogs were evaluated for their cytotoxic activity against three cancer cell lines (MCF-7, PC-3 and COLO-205). The results prompted more investigations to gain a thorough understanding of the mechanism of action of the synthesized compounds. The most potent compounds, **4b** and **4e,** were subjected to further investigation for protein kinase inhibitory activity against the EGFR and CK1 receptors. Moreover, in vitro tubulin polymerization inhibitory activity was evaluated for the most active compound, **4b**.

## 2. Results

### 2.1. Chemistry

**Scheme 1:**
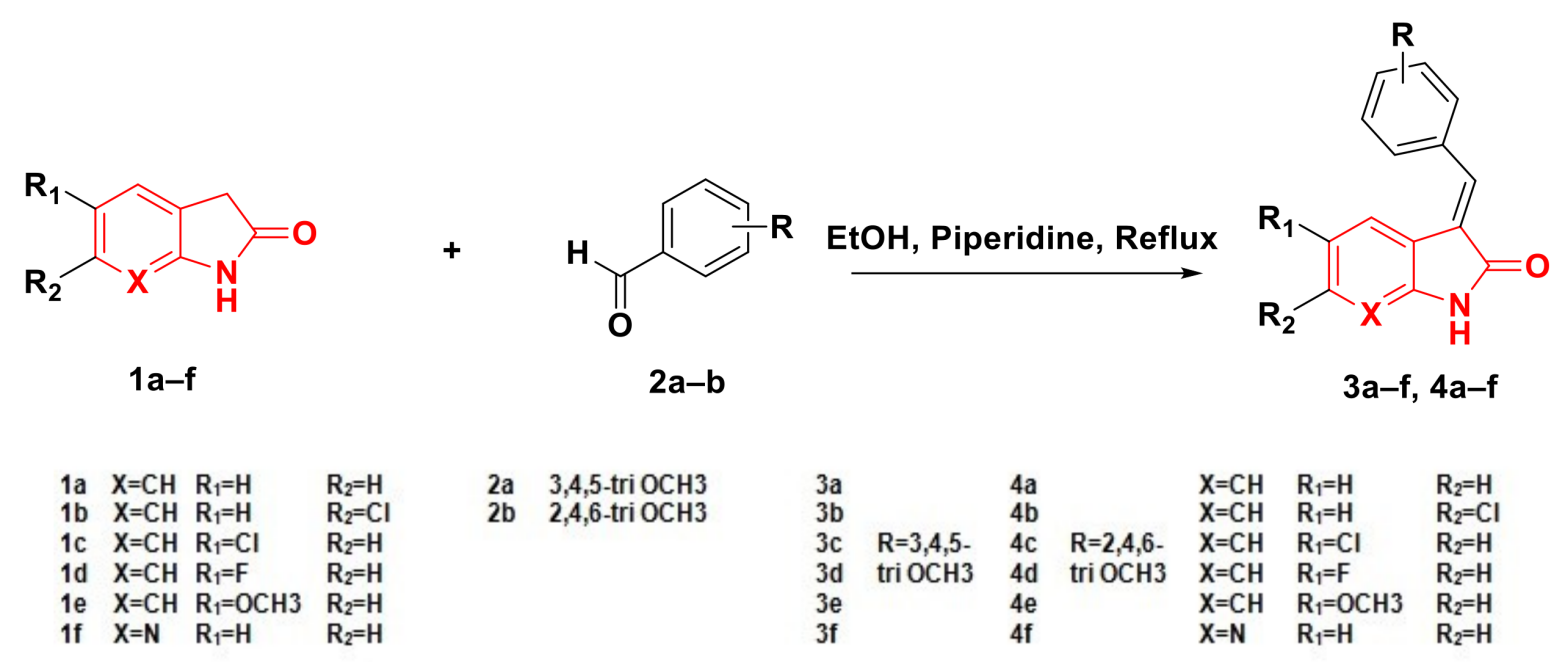
Synthesis of oxindole derivatives (**3a–f**) and (**4a–f**).

Synthesis of the designed substituted oxindole derivatives, **3a**–**f** and **4a**–**f**, started with the condensation of various oxindoles **1a–f** with 3,4,5 or 2,4,6 trimethoxy benzaldehyde **2a** or **2b** in the presence of piperidine as a catalyst, Scheme 1. ^1^H NMR showed a signal for the benzylidine proton at 7.38–7.49 ppm along with NH and other aromatic protons, demonstrating the success of the condensation process. The presence of the olifenic proton at 7.38 ppm affirmed the presence of compounds **3** and **4** in the E configuration. This agrees with previous literature reporting the same reaction [42] in which 2D NMR tools were used to confirm a correlation between the benzylidine proton with the trimethoxyphenyl ring, not the indole H-4 proton, as expected. A possible explanation for such results is the presence of a τ-τ stacking interaction between the phenyl ring and the electron rich indole ring.

**Scheme 2:**
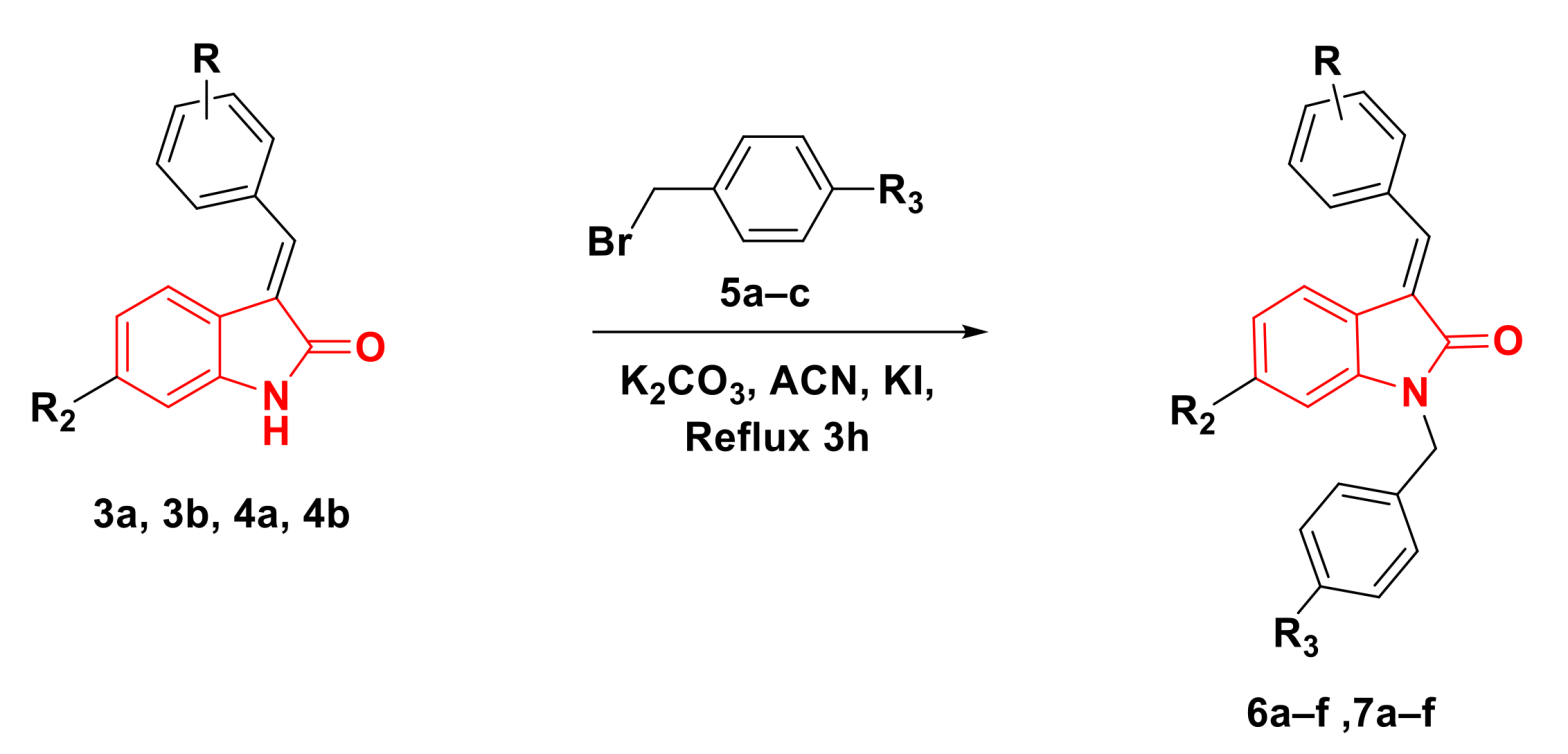

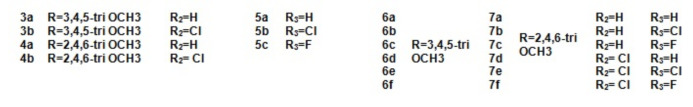
Synthesis of alkylated derivatives **6a–f** and **7a–f**.

**Scheme 3:**
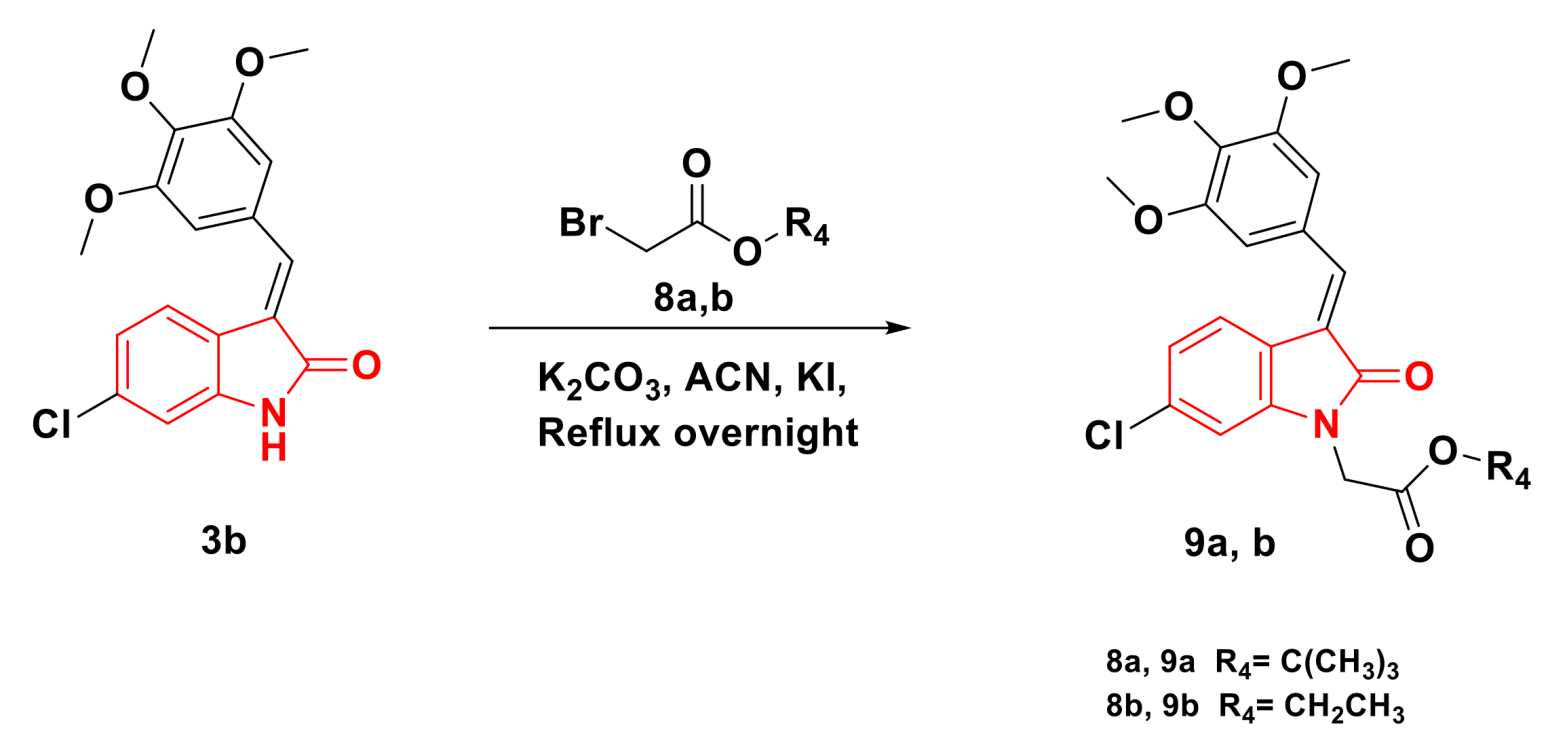
Synthesis of alkylated derivatives **9a** and **9b**.

Further alkylation of **3a–f** and **4a–f** with benzyl bromides **5a–c** in the presence of potassium carbonate and potassium iodide as catalysts afforded the alkylated oxindole derivatives **6a–f** and **7a–f**, as in Scheme 2. Similarly, oxindole derivatives **9a** and **9b** were synthesized through alkylation of compound **3b** with the corresponding ester of 2-bromoacetate, as in Scheme 3. The disappearance of NH signal, and appearance of benzylic CH_2_ protons peak at 4.50–5.00 ppm in ^1^H NMR confirmed the construction of alkylated oxindole derivatives. All other protons appeared at their expected values. ESI/MS data validated the structures of all synthesized compounds, and their purity was ascertained by elemental analysis.

### 2.2. Biological Evaluation 

#### 2.2.1. In Vitro Screening against COLO 205, MCF-7 and PC-3 Cell Lines

Compounds **3a–f**, **4a–f**, **6a–f**, **7a–f** and **9a–b** were used for in vitro anticancer screenings in Nawah Scientific Inc., Egypt. Compounds were screened at two doses (1 and 10 μM) against three cancer cell lines from breast, prostate, and colon (MCF-7, PC-3 and COLO-205) cancer cell lines, respectively. 

The results were recorded as growth inhibition (GI, %) observed on the tested cell lines upon treatment with the test compounds. Experiments were done in triplicate, and results listed as average ± standard error of mean (SEM) and recorded in Table 1. At a 1 μM dose, compounds **4a** and **4b** caused GI of 44 and 46%, respectively, on the MCF-7 cell line, while on the PC-3 cell line, compounds **4a, 4b** and 4e led to 60, 65 and 43% of GI, respectively. On the COLO-205 cell line, compounds 4a and 4b showed GI values of 64 and 74%, Other compounds were not effective on these three cancer cell lines at a dose of 1 μM. Thus, further screenings using concentration of 10 μM were executed.

On the MCF-7 cell line, 10 μM doses of compounds **3b, 3e, 4a–f, 6a, 6d, 7d and 7e** induced GIs of 41–69% while the remaining tested compounds caused GI values less than 40%. Higher GI rates were observed in the PC-3 cell line. Compounds **3e, 4a–f**, and **7e** caused growth inhibition of 52–72%, while other tested compounds caused GI values less than 40%. A greater number of compounds were active against the COLO-205 cell line, as compounds **4a–f, 6b, 6e, 6f, 7f, 9b** elicited GIs of 41–83%, but the other tested compounds caused GI values less than 40%. In summary, the COLO-205 cell line was most sensitive to the tested compounds and was used for all further investigations. 

#### 2.2.2. Antiproliferative Activity against COLO-205 and A459 Cell Lines

Compounds **4b, 4d, 4e, 6e and 6f,** the five compounds with the highest observed activity on COLO-205 cell lines, were selected for determining the concentration required to inhibit 50% of cellular growth (IC_50_) against the COLO-205 cell line compared to compound **4a**. Compound **4a,** or **IC261**, is a known casein kinase (CK1) inhibitor that has been found to affect cell growth and induce cell cycle arrest and apoptosis in different malignant cell lines [43].

The IC_50_ for these compounds was 0.2, 1.0, 0.3, 15.3, and 10.6, respectively (Table 2). Compound **4b** and **4e** were almost equipotent to **4a**, with **4b** being the most potent among the test compounds and used for further mechanistic investigations.

Moreover, the two most active compounds were further tested against the lung cancer A549 cell line using the MTT assay. Compound **4b,** with highest observed anticancer activity, retained its cytotoxicity against the lung cancer line with an IC_50_ of 9.5 μM, being three times more potent than its parent compound **4a** with an IC_50_ of 28 μM, Table 2.

#### 2.2.3. Assay of Tubulin Polymerization Inhibitory Activity

To show the tubulin polymerization inhibitory activity for the desired compounds and to validate the design, the most potent compound **4b** was selected to explore the tubulin polymerization inhibitory activity, in addition to **4a** (IC 261), using combretastatin A4 (CA-4) as a positive control. The obtained results (Table 3) showed that compound **4b** (IC_50_ = 1.66 ± 0.08 μM) preserved tubulin polymerization inhibitory activity, though slightly lower than that of the reference combretastatin A4 (IC_50_ = 0.42 ± 0.02 μM).

#### 2.2.4. Inhibition of EGFR Activity

The EGFR kinase kit assay was performed to assess the EGFR inhibitory activity of compounds **4a**, **4b** and **4e** using Geftinib as a reference drug. Compound **4e** exhibited good inhibition of EGFR-TK with an IC_50_ of 0.401 μg/mL, while compounds **4a** and **4b** were about twice more potent than compound **4e** with IC_50_ = 0.10 and 0.19 μg/mL, which demonstrated comparable activity to the positive reference drug Geftinib (IC_50_ = 0.057 μg/mL). These results confirmed the findings of the cancer-cell based assay and in silico studies (sec 2.3), which indicated that the anticancer activity of both compounds 4b and 4e is partially provided by the EGFR inhibition (Table 3).

#### 2.2.5. Inhibition of Casein Kinase (CK) Activity

Compounds **4b** and **4e** were tested with a Casein Kinase 1 (CK 1) assay against compound **4a (IC261)** as a standard reference compound to evaluate their inhibition potency. The results showed that compound 4e had good anticancer activity on CK1 with an IC_50_ = 13.08 μg/mL, which was about 6.8-fold less potent than compound **4b** that was effective on CK1 with an IC_50_ = 1.92 μg/mL comparable to the standard reference compound **4a** (**IC261**) with an IC_50_ = 2.39 μg/mL. These results are good evidence that the anticancer activities of both compound **4b** and **4e** are related to the Casein Kinase inhibition (Table 3).

#### 2.2.6. Effect on Bax, Bcl-2, Caspase 3 and Cytochrome C Expression Levels 

**Figure 4:**
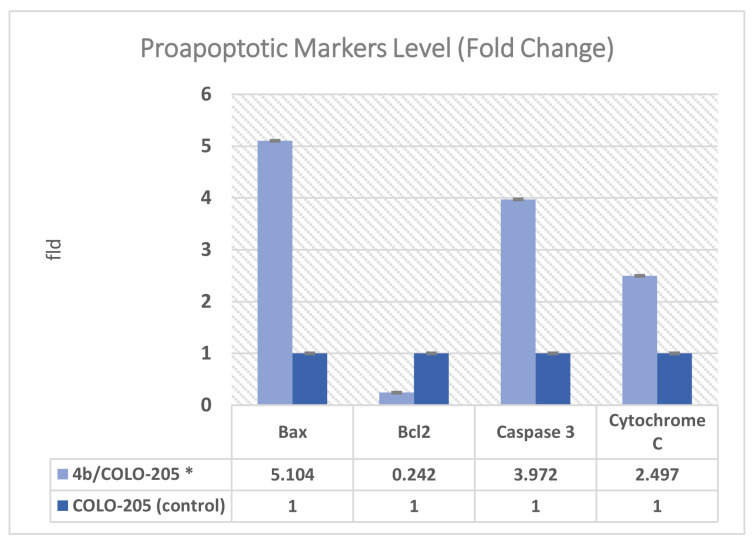
Bax, Bcl-2, Caspase 3 and Cytochrome C expression levels in COLO-205 cells treated with compound **4b** (light blue) or DMSO (dark blue) as control. * Data obtained from this group are significantly different from control with two-tailed *p* value less than 0.0001.

To shed more light on the mechanism of action of compound **4b**, it was further tested for its effect on Bax, Bcl-2, Caspase 3 and Cytochrome C expression levels in the colorectal cancer cell line (COLO-205). Compound **4b** increased the Bax expression level by 5.10-fold compared to the DMSO-treated COLO-205 (Figure 4). In addition, compound **4b** prompted down-regulation of Bcl-2 protein level 4.16-fold lower than untreated COLO cells (Figure 4). The ability of compound **4b** to activate caspase-3 was also tested and compared to the control. Compound **4b** induced accumulation of caspase 3 levels by 3.97-fold compared to the DMSO-treated control cell (Figure 4). Compound **4b** was further investigated for activation of cytochrome C to evaluate its effect on the apoptotic pathway. It was found that compound **4b** increased the cytochrome C level 2.49-fold relative to the DMSO-treated COLO-205 cell (Figure 4). Collective data imply the involvement of **4b** in the apoptotic pathway.

#### 2.2.7. Cell Cycle Analysis

**Figure 5:**
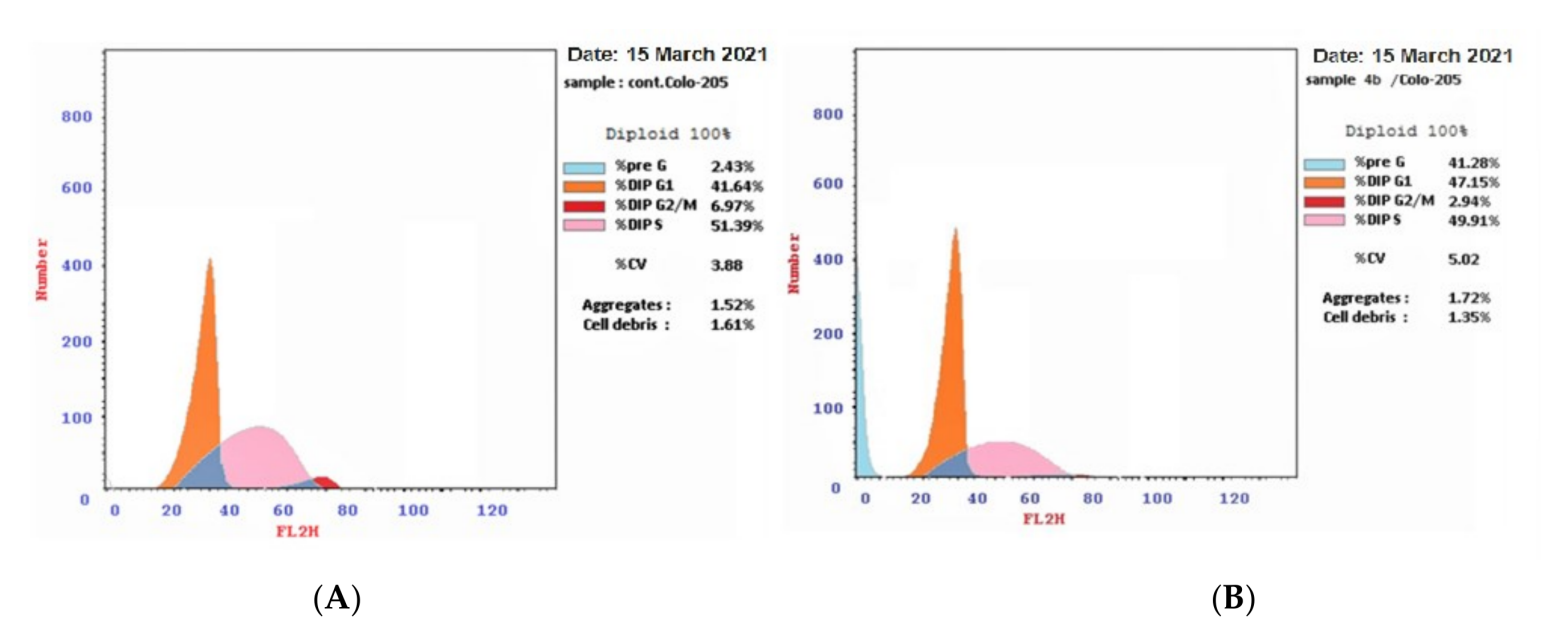
Effect of compound (**A**) **4b**, and (**B**) DMSO as control on the percentage of accumulation of cells at different phases of cell cycle.

To investigate the mechanism used by compound **4b** to exert its antiproliferative activity, the effect of **4b** on cell cycle progression and induction of apoptosis on COLO-205 cell line was carried out using DNA flow cytometric analysis. The resulting data for compound **4b** is shown in Figure 5 and Table 4, displaying remarkable disruption of cell cycle profile and the significant induction of apoptosis compared with that of untreated COLO-205 cells. Treatment of COLO-205 cells by compound **4b** increased the percentage of accumulation of cells at G0-G1 (from 41.46 to 47.15) combined with a decrease in the proportion at the G_2_/M phase and S phase, indicating that compound **4b** arrested cell cycle at G0-G1phase. Additionally, compound **4b** induced apoptosis as observed from the increase in cells at the pre-G1 phase by 16.99-fold (from 2.43 to 41.28, Table 4).

#### 2.2.8. Annexin V-FITC Apoptosis Determination

The ability of compound **4b** to induce apoptosis in COLO-205 and its effects on different stages with flow cytometry were studied using Annexin V/PI staining. During the early stages of apoptosis, phosphatidyltransferase (PS) is translocated from the plasma membrane inner side into the outer surface of cell [44,45]. Labeled Annexin V (conjugated to FITC) interacts with PS and fluoresces green, detecting PS and indicating the early stages of apoptosis [46]. In this assay, propidium iodide (PI), a fluorescent counterstain, intercalates into nucleic acid but only of dead cells (living cells cannot enter) indicating the late stages of apoptosis. This assay can also be used to differentiate between apoptosis and necrosis using PI/Annexin V.

**Figure 6:**
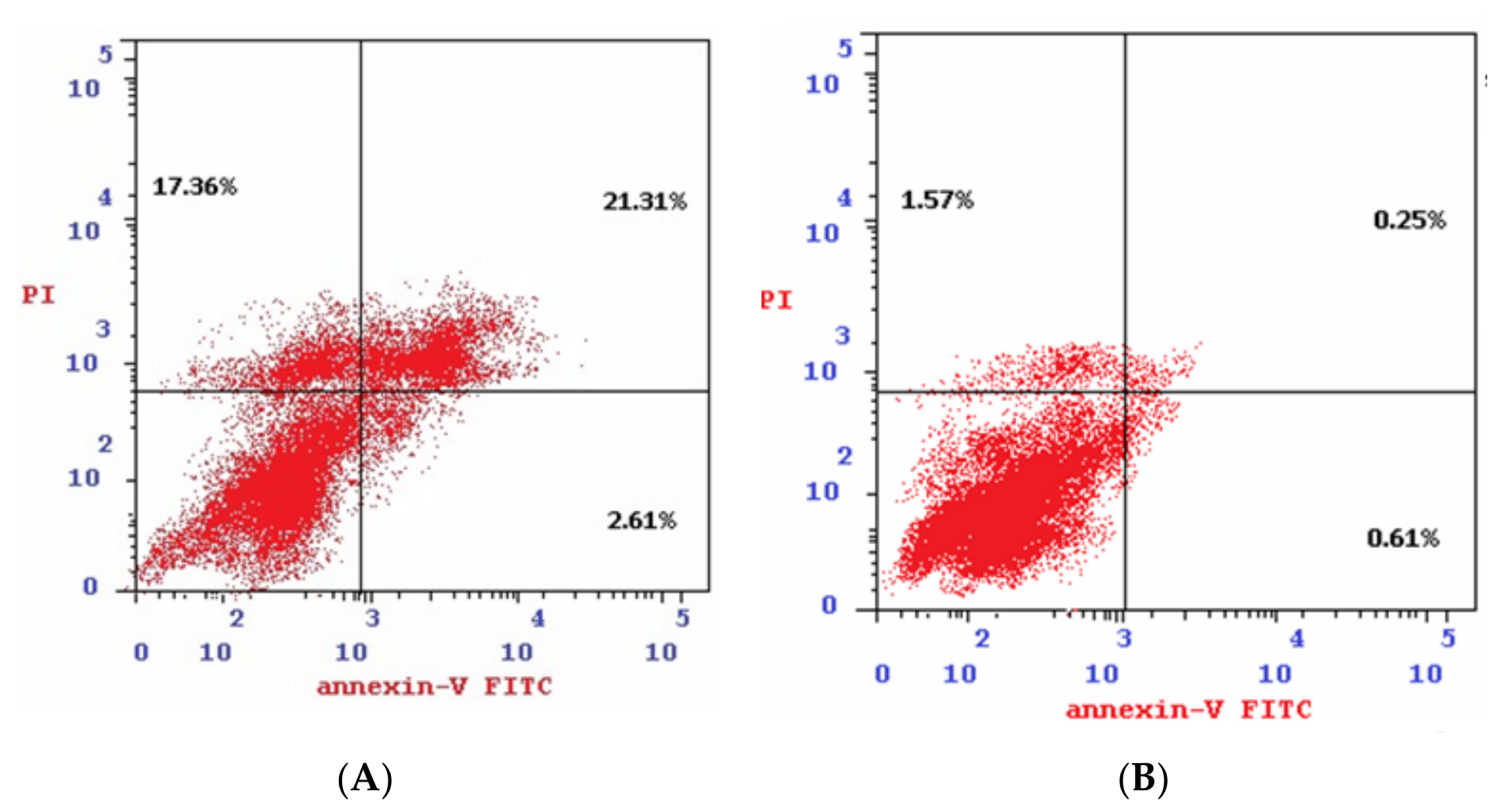
The effect of (**A**) **4b**, (**B**) DMSO (control) on the percentage of annexin V-FITC-positive staining in COLO-205 cells. The experiments were done in triplicate. The four quadrants are identified as LL: viable; LR: early apoptotic; UR: late apoptotic; UL: necrotic.

The results with compound **4b** indicate that it increased the ratio of early apoptosis from 0.61% to 2.61% and increased the ratio of the late apoptosis from 0.25% to 21.31% in comparison to the DMSO-treated COLO-205cells. This revealed that compound **4b** induced both early and late stages of apoptosis up to 27.81 times compared to the control, and via necrosis up to 11.05 times (increased from 1.57% to 17.36%), suggesting that compound **4b** induced apoptosis through a cellular programmed death pathway rather than the necrotic path (Table 4, Figure 6).

This assay not only can be used to differentiate between apoptosis and necrosis using PI/Annexin V but also to distinguish between cytostatic and cytotoxic effects of the tested compounds. The overall effect of compound **4b** is considered cytotoxic, rather than cytostatic.

### 2.3. Molecular Docking Study 

**Figure 7:**
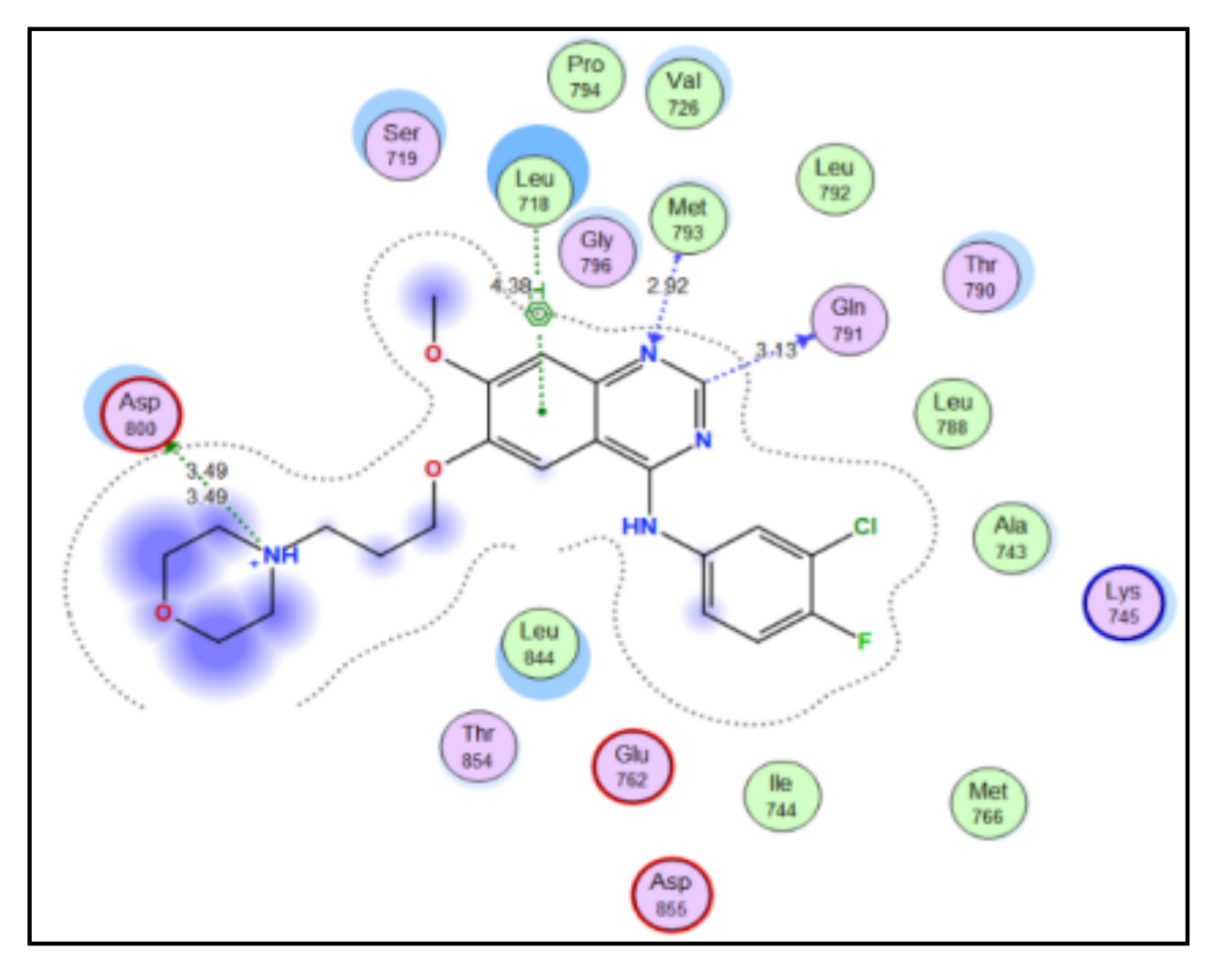
Two-dimensional interaction diagram showing cocrystallized (gefitinib) docking pose interactions with key amino acids in the EGFR binding site.

**Figure 8:**
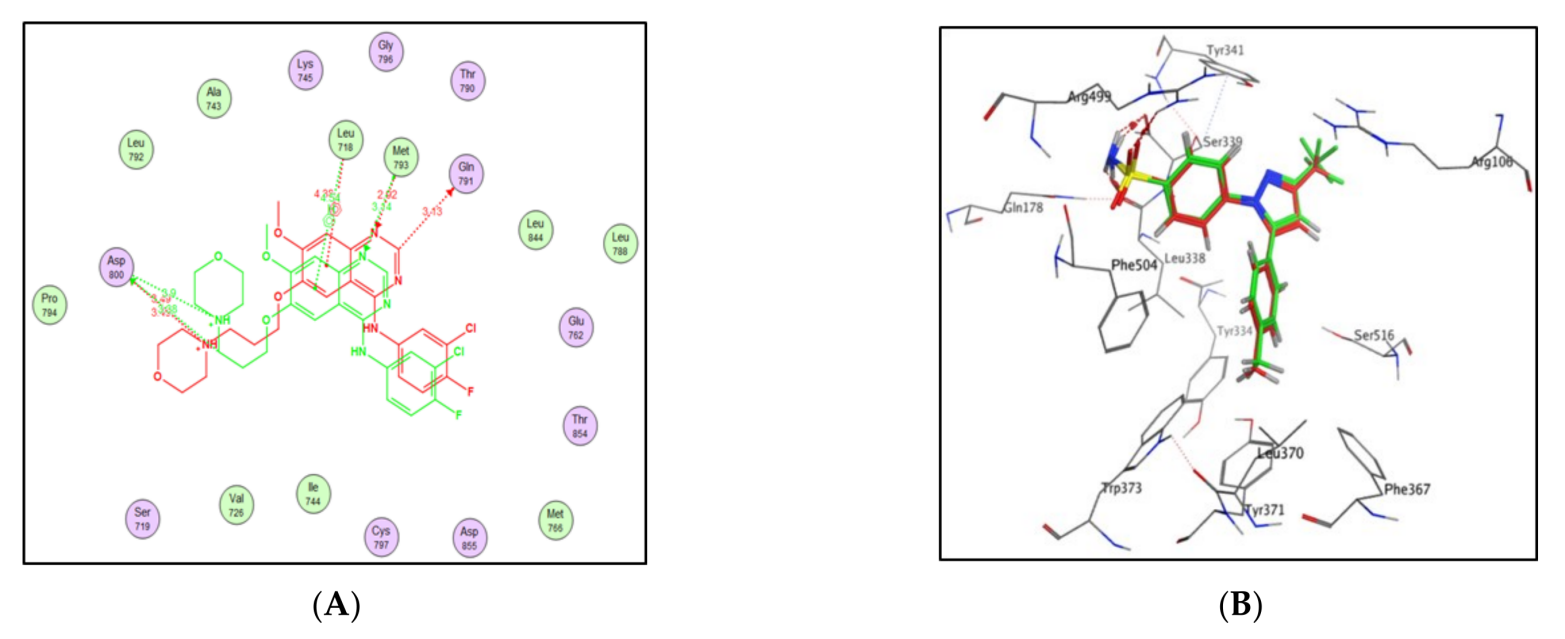
Two-dimensional diagram (**A**) and 3D representation (**B**) of the superimposition of the cocrystallized (red) and the docking pose (blue) of ligand (gefitinib) in the EGFR binding site with RMSD of 1.3 Å.

Docking simulations were performed to study the binding characteristics of the newly synthesized 3-benzylideneindolin-2-one derivatives in the binding site of both EGFR and tubulin. Molecular docking studies were performed by Molecular Operating Environment (MOE, 2010.10) software. The X-ray crystallographic structure of EGFR (PDB ID: 2ITO) was downloaded from the Protein Data Bank (PDB). The docking setup was first validated by performing self-docking of the cocrystallized (gefitinib) ligand in the binding site of EGFR. The self-docking validation step reproduced the experimental binding mode of the cocrystallized (gefitinib) ligand precisely demonstrating that the utilized docking protocol was suitable for the current docking study. This was shown by the small RMSD value between the docked pose and the cocrystallized ligand (1.3 Å), the energy score (S) = −8.1787 kcal/mol and the ability of the docking pose to reproduce all the key interactions attained by the cocrystallized ligand with the hot spots in the EGFR active site (Figure 7 and Figure 8). Consequently, the validated setup was used in anticipating the ligand-target interactions at the binding site for the compounds of interest.

**Figure 9:**
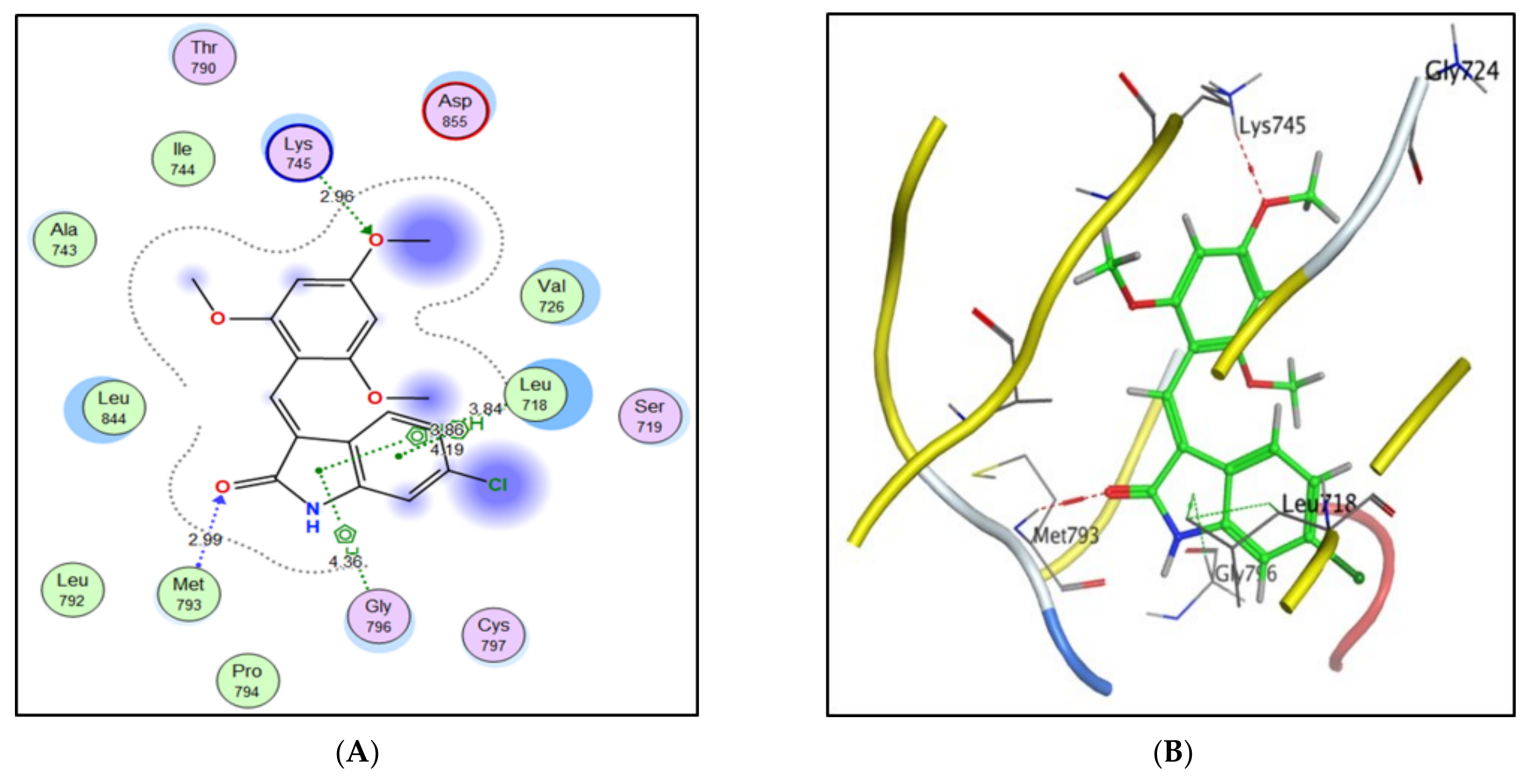
Two-dimensional diagram (**A**) and 3D representation (**B**) of compound **4b** in the EGFR binding site.

Generally, the most active derivative **4b** (IC_50_ = 0.19 ± 0.004 μg/mL) acquired a binding pattern similar to that of Gefitinib in the active site of EGFR (Figure 9) with an energy score of −6.4257 kcal/mol. The indolin-2-one moiety showed important interactions in the adenine region of the EGFR active site, where the carbonyl group at position 2 showed a hydrogen bond with the key amino acid Met793, and the indole nucleus showed four <−H stacking with Leu718 and Gly796. Moreover, the 2,4,6-trimethoxy group accumulated in the hydrophobic pocket and exhibited a hydrogen bond with Lys745.

**Figure 10:**
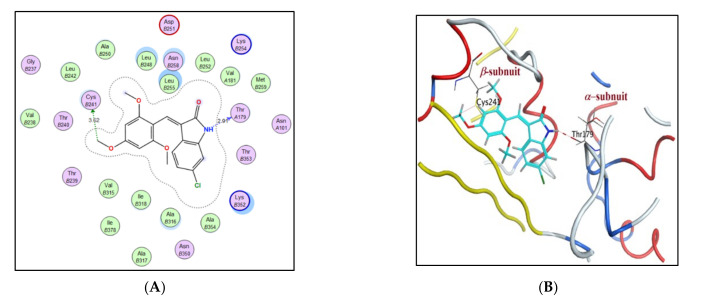
Two-dimensional diagram (**A**) and 3D representation (**B**) of compound **4b** (cyan) with the CA-4 binding site of tubulin (PDB code: 5LYJ).

**Figure 11:**
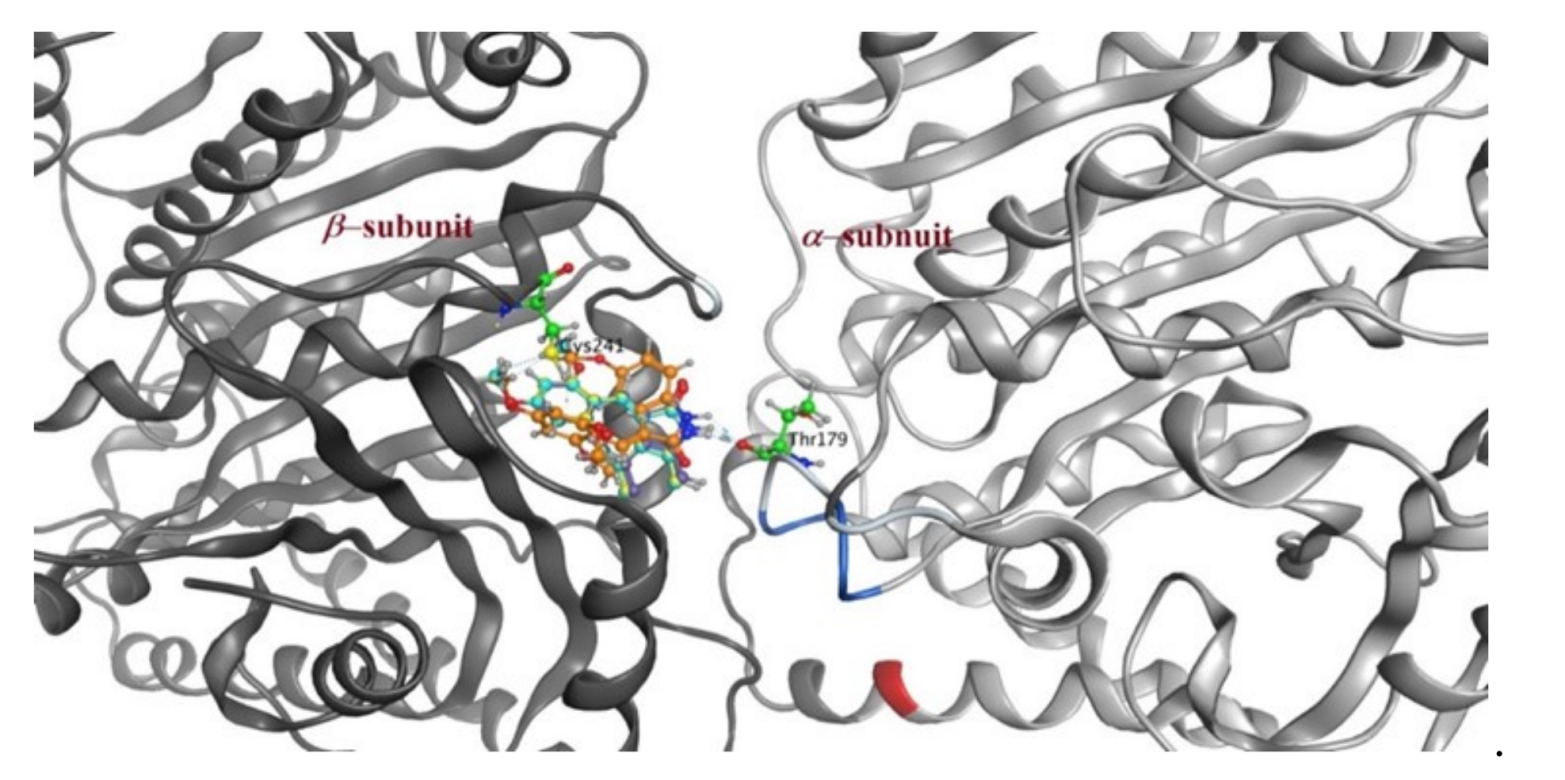
Presumptive binding mode of compound **4a** (yellow), compound **4b** (cyan), compound **4d** (magenta), and compound **4e** (brown). Cys241 is shown on the left and Thr179 is shown on the right. All the docked compounds, key amino acids Cys241 and Thr179 are in stick representation.

Furthermore, the current study was fulfilled to determine the activity of the recently synthesized compounds and to derive structural perceptions into their binding manners and probable interactions within the colchicine binding site of tubulin cocrystallized with CA-4. Accordingly, the most active derivative, **4b,** was docked into the active site of tubulin and the 3D crystal structure of tubulin (PDB code: 5LYJ) was used for this docking simulation study. The binding mode of **4b** (Figure 10), which possessed potent antitubulin activity (IC_50_ = 1.66 ± 0.08 µM) compared to CA-4 (IC_50_ = 0.42 ± 0.02 µM), showed several noncovalent interactions. The p-methoxy group of ring A showed a hydrogen bond with the key amino acid Cys241 in the β-subunit, and the NH group of the indolin-2-one moiety exhibited a hydrogen bond with the key amino acid Thr179 in the α-subunit. Moreover, the role of the indolin-2-one moiety was observed to restrict the cis-orientation of the ethylene bond and prevent rotation of rings A and B. All the docked 3-benzylideneindolin-2-one derivatives (**4a, 4b, 4d and 4e**) showed hydrogen bonding interaction between the p-methoxy group of ring A and the key amino acid Cys241 (Table 5). Compounds **4b** (cyan), **4d** (magenta), and **4e** (brown) were oriented almost the same as CA-4 inside the colchicine binding site (Figure 11). Compounds **4d** and **4e** showed a hydrogen bond with the key Cys241 amino acid. The NH group of indolin-2-one exhibited a hydrogen bond with the key amino acid Thr179 in the α-subunit. Unexpectedly, the energy scores of the docked 3-benzylideneindolin-2-one derivatives were slightly higher than those of CA-4, indicating their slightly reduced interactions with β-tubulin compared with combretastatin A-4, which may be attributed to their bulkier nature.

### 2.4. Physicochemical, ADME and Pharmacokinetic Properties Prediction

**Figure 12:**
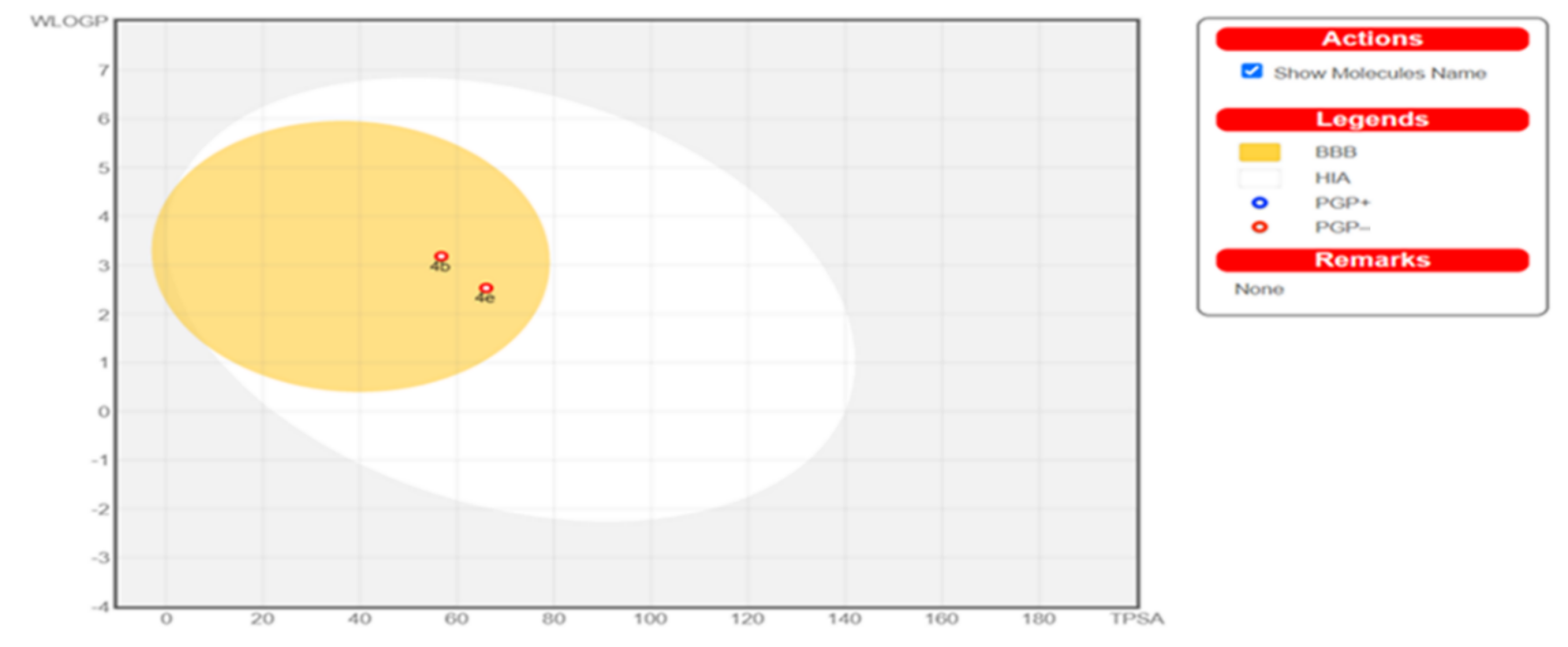
Predicted Boiled-Egg plot from SwissADME online web tool for compounds (**4b** and **4e**).

The freely accessible SwissADME web tool provided by the Swiss Institute of Bioinformatics (SIB, Lausanne, Switzerland) assembles the most relevant computational methods to provide a global appraisal of the pharmacokinetics profile and expectations of the drug-like nature of small molecules [47]. This was performed to assure that the newly synthesized compounds are promising candidates in terms of biological efficacy, and their pharmacokinetic characteristics. The most active 3-benzylideneindolin-2-one derivatives (**4b** and **4e**) exhibited a predicted logP_o__/__w_ value of 3.18 and 2.53, respectively, good water solubility, high GIT absorption and blood brain barrier (BBB) permeability. Figure 12 illustrates a BOILED-Egg graph of the WLOGP vs. TPSA (Topological Polar Surface Area) for the submitted compounds **4b and 4e** [48]. The submitted compounds are located in the zone of human intestinal absorption (HIA) with BBB permeability. Moreover, this graph shows that the submitted compounds were not P-glycoprotein substrates (PGP-), and not amenable to the efflux system effected by this transporter, which is utilized by numerous tumor cells lines as a drug-resistance mechanism.

**Figure 13:**
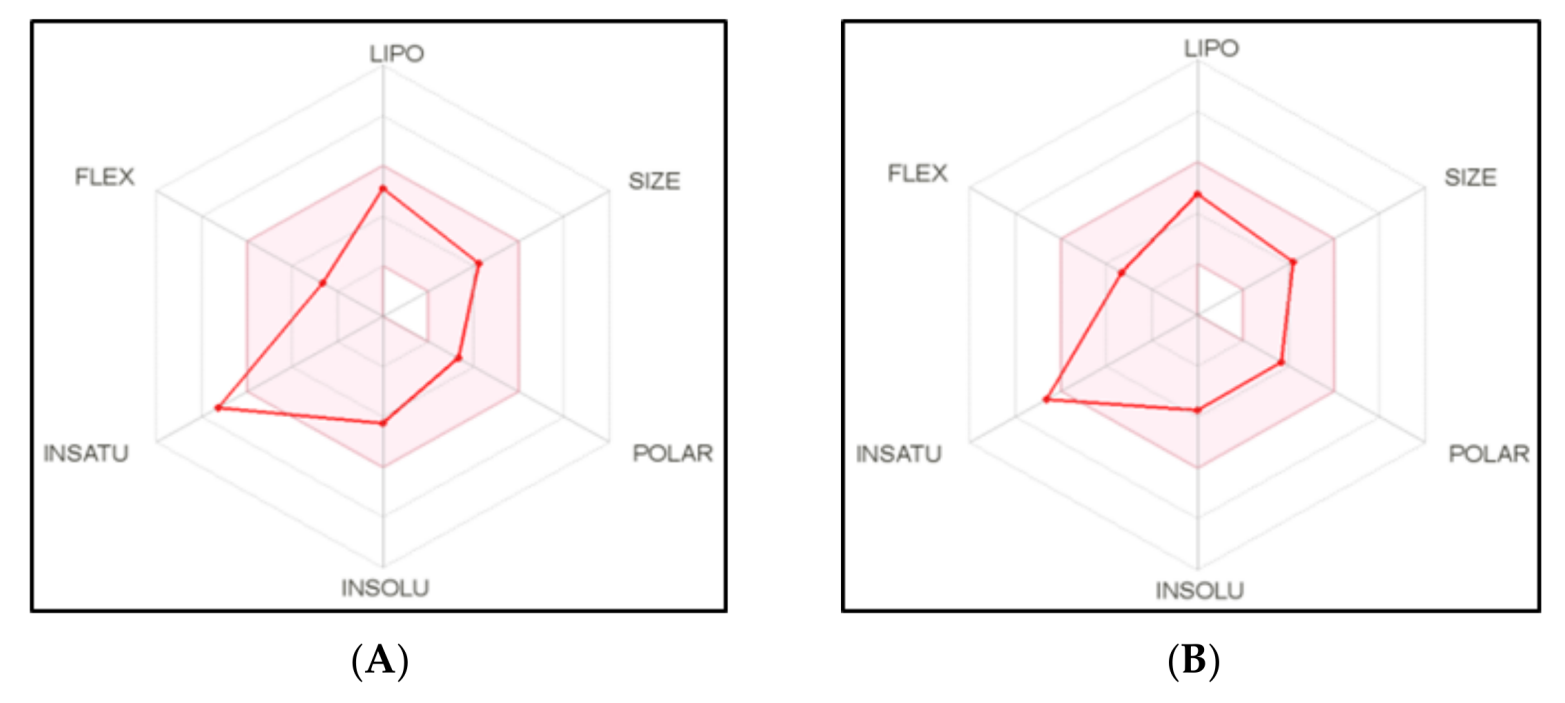
Bioavailability radar chart from Swiss ADME web tool for compounds **4b** (**A**) and **4e** (**B**). The pink area represents the range of the optimal property values for oral bioavailability and the red lines are the predicted properties of **4b** and **4e**.

The predicted high GIT absorption of the tested compounds **4b and 4e** is due to their optimum physicochemical properties located in the suitable physicochemical properties range for oral bioavailability. Figure 13 shows the bioavailability radar chart for compounds **4b** and **4e**. The radar plot bears six axes for six important properties for oral bioavailability: saturation (INSATU), flexibility (FLEX), lipophilicity (LIPO), size (SIZE), polarity (POLAR) and solubility (INSOLU) [48]. The range of the optimal property values is shown as a pink area and the red line represents compounds **4b** (Figure 13A) and **4e** (Figure 13B) whose predicted properties are nearly fully included in the pink area indicating their good predicted oral bioavailability. The SwissADME online web tool showed that the 3-benzylideneindolin-2-one derivatives **4b** and **4e** satisfy drug-likeness characteristics defined by the major pharmaceutical companies: Lipinski’s (Pfizer, New York, NY, USA) [49], Ghose’s (Amgen, Thousand Oaks, CA, USA) [50], and Veber’s (GSK, Brentford, UK) [51] filters. This indicates that the newly synthesized compounds possess promising pharmacokinetic properties and good drug-likeness characteristics.

### 2.5. Pgp Permeability Assay

P glycoprotein (Pgp/Abcb1a) is a membrane-bound efflux transporter that is found in a variety of tissues such as the blood-brain barrier and the blood-brain barrier (CSF) in the brain, intestinal epithelial cells, the bile duct membrane, and the tubules. These transporters identify a variety of xenobiotics as substrates and reduce the systemic exposure of the substrate by minimising absorption through intestinal membranes and potentiating excretion through both bile ducts and the kidney. The ability of Pgp to induce efflux of compound **4b** was assessed using ELISA and the results showed around 35% of Pgp occupied by compound **4b** compared to more than 50% occupied by a standard ketoconazole, Table 6, indicating a higher availability as predicted in the previous section.

## 3. Discussion

The essential role for microtubules in cell mobility, cell shape, cellular transport and cell division infers a major rule for their regulation in diverse pathological ailments, including cancer [52]. The ability of microtubule poisons to restrict tumor cell growth at low doses renders them efficient tools in cancer therapy. However, it is important such drugs should have limited side effects and enhanced physicochemical parameters [53].

The use of indole-2-ones as a potential chemotherapeutics was considered because of their ability to block microtubular polymerization [42,54,55]. Thus, the current research used indole-2one as a basic motif for the design of two series of potential anticancer agents having either a 3,4,5-trimethoxy (compounds 3, 6 and 9) or 2,4,6-trimethoxy phenyl groups (Compounds 4 and 7). Cytotoxicity assays indicated that the observed antiproliferative activity in such compounds is dependent upon the nature of the substitution on the terminal phenyl group. Surprisingly, the 3,4,5-trimethoxy motif essential for **CA4** and colchicine activities, lacks antiproliferative activity in the current setting, with the slight exception of compound **3e**. The presence of 2,4,6-trimethoxy in compound 4 appears crucial for the activity as favored by the elevated activity identified on the three cell lines (MCF-7, PC-3, and COLO-205) used (Table 1). The activity of 3,4,5-trimethoxy-containing derivatives was regained after N-substitution with small aromatic or aliphatic groups as in compounds 6 and 8, while compound 4 lost its activity upon the introduction of N-substitution. The measured cytotoxic outcomes implied that most compounds were more active on COLO-205 cell lines. On human Caucasian colon adenocarcinoma COLO-205 cell lines, compound **4b** had similar activity (IC_50_ = 0.2 μM) to the CK inhibitor **4a** (IC_50_ = 0.2 μM) suggesting a potential enhancement role for a halogen atom (Cl) substitution at position 6 of the indole-2-one ring. Introduction of halogen at position 5 actually decreased the observed activity, as in compounds **4c** and **4d** (GI at 10 μM dose = 58%, 64%). while the presence of a methoxy group in 4e retained the activity with an IC_50_ of 0.3 μM.

Although the literature mentions compound **4a**, **IC261** as a selective CK inhibitor [56], recent studies attributed its apoptotic ability to microtubule polymerization inhibition [38,57], and recently the crystal structure of **4a, IC261** in colchicine binding site was identified [58]. Thus, a reasonable mechanism for the action of the tested compounds was tubulin polymerization inhibition. The ability of 4b to modulate tubulin polymerization was confirmed with an IC_50_ of 1.66 μM compared to CA4 (IC_50_ = 0.42 μM). Theoretically, the docking study reflected the ability of the indolin-2-one moiety to restrict the cis-orientation of the ethylene bond and prevent rotation of rings A and B. A hydrogen bonding interaction between the p-methoxy group of ring A and the key amino acid Cys241 was observed confirming the essential role of a methoxy substitution at ring A (Table 5). Docking also illustrated that designed compounds were oriented almost the same as CA-4 inside the colchicine binding site (Figure 11).

Additionally, a mutual connection between kinase inhibitors and tubulin was established in the literature. Several kinase inhibitors have shown an ability to regulate microtubular function, suggesting that tubulin-targeting medicines could be enhanced. Tivantinib is a c-MET inhibitor that binds to tubulin directly through the colchicine binding site. Microtubule polymerization has also been influenced by kinase inhibitors such as PI3-Akt, FLT3, and JAK-2 inhibitors [59,60]. When compared to single-targeted inhibitors, the use of multitargeted medicines usually results in increased apoptosis and less resistance. Another encouraging aspect is the ability to employ lower doses, resulting in lower toxicity levels [61]. These data show that creating hybrids that include both tubulin and kinase modulators can be effective in cancer treatment.

For example, microtubule inhibition caused inactivation of the EGFR receptor in specific cancer types [62] and caused resensitization of resistant lung cancer cell lines to EGFR inhibitors, suggesting a synergistic effect for both drugs [63]. The discovery of a single compound that has both epidermal growth factor receptor (EGFR) kinase and tubulin polymerization inhibitory capabilities was spurred by clinical evidence for the efficacy of tyrosine kinase inhibitors in combination with microtubule-targeting medicines in cancer therapy [62]. Several researches report this principle in designing dual EGFR/tubulin polymerization inhibitors [64,65,66,67].

The design of the current compounds under investigation as potential binders of EGFR was validated, as compound **4b** induced in vitro EGFR-TK inhibition with an IC_50_ of 0.19 μM compared to Gefitinib (IC_50_ = 0.05 μg/mL). In addition, the suspected ability of the designed compounds to inhibit CK 1, as reported for **4a**, **IC261** (reference compound), was also verified. Compound **4b** induced an inhibition of CK 1 with an IC_50_ of 1.92 μM. This activity was slightly higher than that observed with the standard **4a, IC261** with an IC_50_ of 2.39 μM. The higher potency of **4b** against CK1 possibly clarified **4b**’s higher antiproliferative activity against COLO-205 cell lines. The docking study also favored the obtained data. Compound **4b** had a binding pattern similar to that of gefitinib in the active site of EGFR (Figure 9). Since most protein kinase inhibitors were approved for lung cancer, the obtained kinase inhibitory activity was supported by the cytotoxic activity of **4b** against the lung A549 cancer cell line.

For a deeper understanding of the cellular mechanisms initiated by compound **4b**, its ability to induce cell cycle arrest and apoptosis was explored using flow cytometry. Cell accumulation at the G0-G1 phase suggests that compound **4b** initiates cell cycle arrest at that stage. Additionally, flow cytometry data suggest extensive induction of apoptosis upon treatment of the COLO-205 cell line with compound **4b**. This was evidenced by the rise in the level of accumulated cells in pre G1 phase to 41.28% from 2.43% in untreated cells. Pre-G1 cell accumulation is closely connected to apoptosis. The ability of **4b** to initiate apoptosis was also supported by annexitin flow cytometry, as shown in Figure 5 and Figure 6, and Table 4.

Bcl-2 proteins are important regulators in apoptotic cell death. Bcl-2 is considered anti-apoptotic as it is proficient suppressor of apoptosis [68] and its elevated levels are usually associated with tumor cell resistance to apoptosis [69]. Microtubule poisons such as vincristine, taxol, and colchicine are reported to phosphorylate the Bcl-2 protein at serine 70 and serine 87 residues in tumor cells [70,71]. Bcl-2 phosphorylation at these residues results in inactivation of antiapoptotic function, and the apoptotic pathway prevails [68]. Other mechanisms might be involved in tubulin-induced Bcl-2 deactivation. These pathways involve JAK and other BH3-only proteins such as Bim and Bmf [72]. Compound 4b, in agreement with other tubulin polymerization inhibitors, provoked a reduced level of Bcl-2 in COLO-205 cells to 0.24-fold less than control cells.

Apoptosis was also proved by an increased level of cleaved caspase 3. Caspase activation is one of the most important factors in the initiation and continuance of apoptosis [73], with caspase 3 being the chief player among all caspases. [74]. The ability of 4b to increase levels of caspase 3 up to 4-fold suggests that apoptosis induction was linked to a malfunction of cell cycle control proteins. These data are in accordance with previous reports associating the ability of tubulin inhibitors with upregulating caspase-3 expression level [70,75]. Additionally, cytochrome C has been found to have a fundamental role in governing apoptosis, mainly through interaction with apoptotic protease activating factors (Apaf). It is also involved in activation of cascades of caspases [76]. The ability of 4b to induce levels of cytochrome C was also measured and found to increase cytochrome C levels by 2.5-fold. These data collectively corroborate the ability of 4b to provoke antiproliferative activity by exploiting apoptosis and deregulating levels of cell cycle regulating proteins through their impact on both tubulin and certain kinases such as EGFR and CK1. Besides, the in-silico study of the physicochemical and pharmacokinetic properties confirmed that the newly synthesized compounds are not only have promising antiproliferative activity but also possess promising pharmacokinetic properties and good drug-likeness characteristics.

## 4. Materials and Methods

### 4.1. Chemistry

All chemical compounds and organic solvents used to synthesize the desired compounds were of commercial grade and bought from Alfa Aesar (Haverhill, MA, USA), Sigma Aldrich (St. Louis, MI, USA), Merck (Kenilworth, NJ, USA), and El-gomhoria (Cairo, Egypt) companies for pharmaceutical and chemical compounds and used without further purification. Reaction progress was monitored using precoated TLC plates (Kiesslgel 60 F254, Merck, Kenilworth, NJ, USA), and detecting spots was done via UV lamb at a wavelength of 254 nm. Melting points were measured on Stuart Electro-Thermal apparatus and recorded without correction. ^1^HNMR was carried out on a JOEL JNM-ECA400 (^1^H: 400 MHz) spectrophotometer at Zagazig University, Egypt. ^13^CNMR was carried out on a JOEL JNM-ECA400 (^13^C: 100 MHz) spectrophotometer at Zagazig University, Egypt.

Chemical shifts were recorded in parts per million (ppm) compared to standard TMS using deuterated DMSO (δ:2.5) as a solvent, and the coupling constant (J) was measured in Hertz (Hz). Multiplicity was designated as s singlet; d doublet; t triplet; q quartet; p pentet; dd doublet of doublet and m for multiplet. Elemental analysis was performed at the regional centre for mycology and biotechnology, Al-Azhar University, Assiut, Egypt. LC/MS data were recorded on the LC/MS/MS (Agilent 1260 Infinity II (Santa Clara, CA, USA) with 6420 Triple Quad LC/MS detector) at Nawah, Mokatam, Egypt.

#### 4.1.1. General Procedure for the Synthesis of Compounds **3a–f** and **4a–f**

A mixture of the appropriate oxindole **1** (1 mmol), **2a** or **2b** (1 mmol), and a catalytic amount of piperidine in ethanol (10 mL) was heated at reflux for 3–6 h. Reaction progress was monitored via TLC to confirm product formation, then the reaction was left to cool, and the formed precipitate was filtered and washed with methanol. The obtained residue was crystallised from ethanol to yield compounds **3a–f** and **4a–f** [54].

Synthesis of (E)-3-(3,4,5-trimethoxybenzylidene)indolin-2-one (**3a**) [54].

Yellow solid; 85% yield (170 mg); m.p. = 231–233 °C, reported m.p (232–234 °C); R_F_ = 0.20 (mobile phase is pet.ether:EtOAc, 3:1).

Synthesis of (E)-6-chloro-3-(3,4,5-trimethoxybenzylidene)indolin-2-one (**3b**) [42].

Yellow solid; 86% yield(175 mg); m.p. = 262–264 °C; R_F_ = 0.26 (mobile phase is pet.ether:EtOAc, 3:1). ESI/MS: *m*/*z* Calcd. For [2M + Na]^+^: 712.82, Found: 713.40.

Synthesis of (E)-5-chloro-3-(3,4,5-trimethoxybenzylidene)indolin-2-one (**3c**) [54].

Orange solid; 55% yield(110 mg); m.p. = 263–268 °C, reported m.p. (265–270 °C); R_F_ = 0.30 (mobile phase is pet.ether:EtOAc, 3:1).

Synthesis of (E)-5-fluoro-3-(3,4,5-trimethoxybenzylidene)indolin-2-one (**3d**) [54].

Yellow solid; 50% yield(97 mg); m.p. = 192–194 °C, reported m.p. (193–195 °C); R_F_ = 0.45 (mobile phase is pet.ether:EtOAc, 3:1).

Synthesis of (E)-5-methoxy-3-(3,4,5-trimethoxybenzylidene)indolin-2-one (**3e**) [54].

Orange solid; 50% yield(95 mg); m.p. = 215–218 °C, reported m.p. (217–219 °C); R_F_ = 0.60 (mobile phase is pet.ether:EtOAc, 1:1).

Synthesis of (E)-3-(3,4,5-trimethoxybenzylidene)-1,3-dihydro-2H-pyrrolo[2,3-b] pyridin-2-one (**3f**) [54].

Yellow solid; 50% yield(100 mg); m.p. = 200–202 °C; R_F_ = 0.30 (mobile phase is pet.ether:EtOAc, 1:1).

Synthesis of (E)-3-(2,4,6-trimethoxybenzylidene)indolin-2-one (**4a**, **IC261**).

Yellow solid; 75% yield(158 mg); m.p. = 186–189 °C, reported m.p. 187–188 °C; R_F_ = 0.34 (mobile phase is pet.ether:EtOAc, 1:1).

Synthesis of (E)-6-chloro-3-(2,4,6-trimethoxybenzylidene)indolin-2-one (**4b**).

Yellow solid; 80% yield (163 mg); m.p. = 210–213 °C; R_F_ = 0.22 (mobile phase is pet.ether:EtOAc, 3:1). ^1^H NMR (400 MHz, DMSO-d_6_) δ 10.51 (s, 1H, NH), 7.38 (s, 1H, CH), 6.83 (dd, *J* = 8.2, 2.0 Hz, 1H, ind-5), 6.76 (d, *J* = 2.0 Hz, 1H, ind-7), 6.70 (d, *J* = 8.2 Hz, 1H, ind-4), 6.28 (s, 2H, Ar-H), 3.80 (s, 3H, OCH_3_), 3.69 (s, 6H, 2OCH_3_).^13^C NMR (100 MHz, DMSO-d_6_) δ 169.4, 163.53, 159.58, 143.60, 133.17, 128.34, 126.47, 125.54, 121.84,121.06, 109.70, 104.94, 91.26, 56.09, 55.99. Anal. Calcd. for C_18_H_16_ClNO_4_ (345.08): C, 62.52; H, 4.66; N, 4.05, Found: C, 62.54; H, 4.67; N, 4.06. ESI/MS: *m*/*z* Calcd. for [M-1]^−^: 344.08, Found: 344.20.

Synthesis of (E)-5-chloro-3-(2,4,6-trimethoxybenzylidene)indolin-2-one (**4c**).

Reddish brown solid; 55% yield(110 mg); m.p. = 244–247 °C; R_F_ = 0.40 (mobile phase is pet.ether: EtOAc, 3:1). ESI/MS: *m*/*z* Calcd. For [M + Na]^+^: 458.10, Found: 458.20.

Synthesis of (E)-5-fluoro-3-(2,4,6-trimethoxybenzylidene)indolin-2-one (**4d**).

Yellow solid; 50% yield(95 mg); m.p. = 212–215 °C; R_F_ = 0.48 (mobile phase is pet.ether:EtOAc, 3:1). ^1^H NMR (400 MHz, DMSO-d_6_) δ 10.45 (s, 1H, NH), 7.47 (s, 1H, CH), 6.99 (dd, *J* = 8.5, 2.5 Hz, 1H, ind-6), 6.79 (dd, *J* = 2.5, 0.8 Hz, 1H, ind-4), 6.51 (dd, *J* = 8.5, 0.8 Hz, 1H, ind-7), 6.38 (s, 2H, Ar-H), 3.88 (s, 3H, OCH_3_), 3.77 (s, 6H, 2OCH_3_). ^13^C NMR (100 MHz, DMSO-d_6_) δ 169.43, 163.64, 159.57, 156.74, 138.62, 129.08, 127.26, 124.01, 115.13, 110.93, 110.25, 104.77, 91.32, 56.14, 56.02. Anal. Calcd. for C_18_H_16_FNO_4_ (329.11): C, 65.65; H, 4.90; N, 4.25, Found: C, 65.67; H, 4.91; N, 4.26. ESI/MS: *m*/*z* Calcd. For [M + Na]^+^: 352.10, Found: 452.20.

Synthesis of (E)-5-methoxy-3-(2,4,6-trimethoxybenzylidene)indolin-2-one (**4e**).

Brick red solid; 65% yield(120 mg); m.p. = 221–223 °C; R_F_ = 0.46 (mobile phase is pet.ether:EtOAc, 1:1). ^1^H NMR (400 MHz, DMSO-d_6_) δ 10.24 (s, 1H, NH), 7.39 (s, 1H, CH), 6.76 (dd, *J* = 8.4, 2.4 Hz, 1H, ind-6), 6.71 (d, *J* = 8.4 Hz, 1H, ind-7), 6.40 (d, *J* = 2.4 Hz, 1H, ind-4), 6.37 (s, 2H, Ar-H), 3.87 (s, 3H, OCH_3_), 3.76 (s, 6H, 2OCH_3_), 3.59 (s, 3H, OCH_3_). ^13^C NMR (100 MHz, DMSO-d_6_) δ 169.24, 163.08, 159.25, 154.37, 135.95, 128.11, 127.62, 123.60, 114.07, 110.57, 109.63, 104.79, 90.96, 55.91, 55.76, 55.59. Anal. Calcd. for C_19_H_19_NO_5_ (341.13): C, 66.85; H, 5.61; N, 4.10, Found: C, 66.87; H, 5.62; N, 4.11. ESI/MS: *m*/*z* Calcd. For [M-1]^−^: 340.13, Found [M-1]^−^: 340.30.

Synthesis of (E)-3-(2,4,6-trimethoxybenzylidene)-1,3-dihydro-2H-pyrrolo[2,3-b] pyridin-2-one (**4f**) [77].

Yellow solid; 50% yield(95 mg); m.p. = 174–176 °C, reported m.p. (176–178 °C); R_F_ = 0.3 (mobile phase is pet.ether:EtOAc, 1:1).

#### 4.1.2. General Procedures for the Synthesis of Compounds **6a–f**, **7a–f**

A mixture of the appropriate oxindole (**3a, 3b, 4a or 4b**,1 mmol) was stirred with K_2_CO_3_ (690 mg, 5 mmol) in acetonitrile (10 mL) for 30 min. The corresponding benzyl bromide derivative **5a–c** (1 mmol) was then added portion-wise with KI (830 mg, 5 mmol). The mixture was then refluxed for 3 h. Product formation was followed up by TLC, then reaction mixture was concentrated under reduced pressure and the remaining mixture was extracted using ethyl acetate (3 × 30 mL) and washed with water (3 × 30 mL). The organic layers were collected and dried over anhydrous sodium sulphate, solvent was evaporated, and the obtained product [42] was purified by silica gel column chromatography using (petroleum ether: ethyl acetate, 7:3) as mobile phase.

Synthesis of (E)-1-Benzyl-3-(3,4,5-trimethoxybenzylidene)indolin-2-one (**6a**).

Yellow liquid; 83% yield (165 mg); R_F_ = 0.42 (mobile phase is pet.ether:EtOAc, 3:1). ^1^H NMR (400 MHz, DMSO-d_6_) δ 8.08 (s, 1H, CH), 7.75 (s, 2H, Ar-H), 7.36–7.30 (m, 5H, Ar-H), 7.13–6.90 (m, 4H, ind-4-7), 5.02 (s, 2H, CH_2_), 3.87 (s, 6H, 2OCH_3_), 3.76 (s, 3H, OCH_3_). ^13^C NMR (100 MHz, DMSO-d_6_) δ 167.55, 165.60, 152.91, 142.98, 138.33, 137.32, 136.59, 129.90, 128.67, 127.30, 125.54, 124.10, 121.90, 120.45, 119.32, 110.41, 107.23, 60.22, 56.03, 42.69. Anal. Calcd. for C_25_H_23_NO_4_ (401.16): C, 74.80; H, 5.77; N, 3.49, Found: C, 74.82; H, 5.78; N, 3.50. ESI/MS: *m*/*z* Calcd. For [M + Na]^+^: 424.15, Found: 424.20.

Synthesis of (E)-1-(4-Chlorobenzyl)-3-(3,4,5-trimethoxybenzylidene)indolin-2-one (**6b**) [78].

Yellow liquid; 87% yield(173 mg); R_F_ = 0.35 (mobile phase is pet.ether: EtOAc, 3:1). ESI/MS: *m*/*z* Calcd. For [M + Na]^+^: 458.10, Found: 458.20.

Synthesis of (E)-1-(4-Fluorobenzyl)-3-(3,4,5-trimethoxybenzylidene)indolin-2-one (**6c**).

Yellow liquid; 55% yield(112 mg); R_F_ = 0.26 (mobile phase is pet.ether:EtOAc, 3:1). ^1^H NMR (400 MHz, DMSO-d_6_) δ 7.55 (s, 1H, CH), 7.19–7.13 (m, 4H, ind-4-7), 6.95 (d, *J* = 8.5 Hz, 2H, Ar-H), 6.87 (d, *J* = 8.5 Hz, 2H, Ar-H), 6.36 (s, 2H, Ar-H), 4.93 (s, 2H, CH_2_), 3.86 (s, 3H, OCH_3_), 3.75 (s, 6H, 2OCH_3_). ^13^C NMR (100 MHz, DMSO-d_6_) δ 167.64, 163.08, 159.18, 141.92, 129.84, 129.77, 129.61, 129.53, 128.68, 125.99, 123.77, 121.65, 115.58, 115.37, 108.61, 104.58, 90.84, 55.68, 55.58, 41.92. Anal. Calcd. for C_25_H_22_FNO_4_ (419.15): C, 71.59; H, 5.29; N, 3.34, Found: C, 71.57; H, 5.30; N, 3.35. ESI/MS: *m*/*z* Calcd. For [M + Na]^+^: 442.14, Found: 442.20.

Synthesis of (E)-1-benzyl-6-chloro-3-(3,4,5-trimethoxybenzylidene)indolin-2-one (**6d**).

Yellow liquid; 65% yield(127 mg); R_F_ = 0.34 (mobile phase is pet.ether:EtOAc, 1:1). ^1^H NMR (400 MHz, DMSO-d_6_) δ 8.06 (s, 1H, CH), 7.78 (d, *J* = 1.8 Hz, 1H, ind-7), 7.38 (dd, *J* = 7.8, 1.7 Hz, 2H, Ar-H), 7.36 (s, 2H, Ar-H), 7.32 (dd, *J* = 7.8, 1.7 Hz, 2H, Ar-H), 7.30–7.27 (m, 1H, Ar-H), 7.13 (d, *J* = 7.5 Hz, 1H, ind-4), 7.07 (dd, *J* = 7.5, 1.8 Hz, 1H, ind-5), 5.02 (s, 2H, CH_2_), 3.82 (s, 6H, 2OCH_3_), 3.76 (s, 3H, OCH_3_). ^13^C NMR (100 MHz, DMSO-d_6_) δ 167.78, 163.38, 159.28, 143.32, 136.58, 132.91, 129.05, 128.74, 127.49, 127.38, 125.04, 124.67, 121.35, 120.72, 108.86, 104.43, 90.90, 55.72, 55.62, 42.64. Anal. Calcd. for C_25_H_22_ClNO_4_ (435.12): C, 68.89; H, 5.09; N, 3.21, Found: C, 68.91; H, 5.10; N, 3.22. ESI/MS: *m*/*z* Calcd. For [M + Na]^+^: 458.11, Found: 458.20.

Synthesis of (E)-6-chloro-1-(4-chlorobenzyl)-3-(3,4,5-trimethoxybenzylidene) indolin-2-one (**6e**).

Yellow liquid; 85% yield(169 mg); R_F_ = 0.32 (mobile phase is pet.ether:EtOAc, 3:1). ^1^H NMR (400 MHz, DMSO-d_6_) δ 8.05 (s, 1H, CH), 7.79 (d, *J* = 2.0 Hz, 1H, ind-7), 7.44 (d, *J* = 8.7 Hz, 2H, Ar-H), 7.40 (d, *J* = 8.7 Hz, 2H, Ar-H), 7.17 (d, *J* = 8.3 Hz, 1H, ind-4), 7.11 (s, 2H, Ar-H), 7.06 (dd, *J* = 8.3, 2.0 Hz, 1H, ind-5), 5.02 (s, 2H, CH_2_), 3.83 (s, 6H, 2OCH_3_), 3.77 (s, 3H, OCH_3_). ^13^C NMR (100 MHz, DMSO-d_6_) δ 168.09, 153.41, 152.80, 144.57, 138.75, 134.57, 132.62, 129.69, 129.56, 129.18, 128.65, 124.83, 123.34, 122.27, 111.00, 110.07, 107.76, 60.69, 56.51, 42.52. Anal. Calcd. for C_25_H_21_Cl_2_NO_4_ (469.08): C, 63.84; H, 4.50; N, 2.98, Found: C, 63.86; H, 4.51; N, 2.99. ESI/MS: *m*/*z* Calcd. For [M + Na]^+^: 492.07, Found: 492.20.

Synthesis of (E)-6-chloro-1-(4-fluorobenzyl)-3-(3,4,5-trimethoxybenzylidene) indolin-2-one (**6f**).

Yellow liquid; 85% yield(175 mg); R_F_ = 0.4 (mobile phase is pet.ether:EtOAc, 3:1). 1H NMR (400 MHz, DMSO-d_6_) δ 8.04 (s, 1H, CH), 7.77 (d, *J* = 2.0 Hz, 1H, ind-7), 7.18 (d, *J* = 8.2 Hz, 2H, Ar-H), 7.17 (d, *J* = 8.2 Hz, 2H, Ar-H), 7.11 (d, *J* = 8.3 Hz, 1H, ind-4), 7.09 (s, 2H, Ar-H), 7.04 (dd, *J* = 8.3, 2.0 Hz, 1H, ind-5), 4.99 (s, 2H, CH_2_), 3.81 (s, 6H, 2OCH_3_), 3.75–3.73 (s, 3H, OCH_3_). ^13^C NMR (100 MHz, DMSO-d_6_) δ 167.61, 165.58, 152.95, 152.33, 144.14, 139.14, 138.22, 134.10, 129.51, 129.43, 124.41, 123.83, 121.74, 119.40, 115.42, 110.53, 107.29, 60.21, 56.03, 41.98. Anal. Calcd. for C_25_H_21_ClFNO_4_ (453.11): C, 66.16; H, 4.66; N, 3.09, Found: C, 66.18; H, 4.67; N, 3.10. ESI/MS: *m*/*z* Calcd. For [M + Na]^+^: 476.10, Found: 476.30.

Synthesis of (E)-1-benzyl-3-(2,4,6-trimethoxybenzylidene)indolin-2-one (**7a**).

Yellow liquid; 83% yield(165 mg); R_F_ = 0.42 (mobile phase is pet.ether:EtOAc, 3:1). ^1^H NMR (400 MHz, DMSO-d_6_) δ 7.65 (s, 1H, CH), 7.37–7.33 (m, 5H, Ar-H), 6.95–6.87 (m, 4H, ind-4-7), 6.38 (s, 2H, Ar-H), 4.97 (s, 2H, CH_2_), 3.83 (s, 3H, OCH_3_), 3.77 (s, 6H, 2OCH_3_). ^13^C NMR (100 MHz, DMSO-d_6_) δ 167.55, 165.60, 152.91, 142.98, 138.33, 137.32, 136.59, 129.90, 128.67, 127.30, 125.54, 124.10, 121.90, 120.45, 119.32, 110.41, 107.23, 60.22, 56.03, 42.69. Anal. Calcd. for C_25_H_23_NO_4_ (401.16): C, 74.80; H, 5.77; N, 3.49, Found: C, 74.82; H, 5.78; N, 3.50. ESI/MS: *m*/*z* Calcd. For [M + Na]^+^: 424.15, Found: 424.20.

Synthesis of (E)-1-(4-chlorobenzyl)-3-(2,4,6-trimethoxybenzylidene)indolin-2-one (**7b**).

Yellow liquid; 80% yield(155 mg); R_F_ = 0.36 (mobile phase is pet.ether:EtOAc, 3:1). ^1^H NMR (400 MHz, DMSO-d_6_) δ 7.57 (s, 1H, CH), 7.41 (d, *J* = 8.7 Hz, 2H, Ar-H), 7.37 (d, *J* = 8.7 Hz, 2H, Ar-H), 6.99–6.84 (m, 4H, ind-4-7), 6.37 (s, 2H, Ar-H), 4.96 (s, 2H, CH_2_), 3.88 (s, 3H, OCH_3_), 3.76 (s, 6H, 2OCH_3_). ^13^C NMR (100 MHz, DMSO-d_6_) δ 167.56, 152.91, 152.32, 142.77, 138.97, 137.45, 135.65, 132.05, 129.21, 129.09, 128.67, 128.19, 125.43, 122.01, 120.46, 110.41, 107.23, 60.22, 56.03, 42.03. Anal. Calcd. for C_25_H_22_ClNO_4_ (435.12): C, 68.89; H, 5.09; N, 3.49, Found: C, 68.91; H, 5.10; N, 3.48. ESI/MS: *m*/*z* Calcd. For [M + Na]^+^: 458.10, Found: 458.20.

Synthesis of (E)-1-(4-fluorobenzyl)-3-(2,4,6-trimethoxybenzylidene)indolin-2-one (**7c**).

Yellow liquid; 65% yield(150 mg); R_F_ = 0.26 (mobile phase is pet.ether:EtOAc, 3:1). ^1^H NMR (400 MHz, DMSO-d_6_) δ 7.55 (s, 1H, CH), 7.19–7.13 (m, 4H, ind-4-7), 6.95 (d, *J* = 7.4 Hz, 2H, Ar-H), 6.87 (d, *J* = 7.4 Hz, 2H, Ar-H), 6.36 (s, 2H, Ar-H), 4.93 (s, 2H, CH_2_), 3.86 (s, 3H, OCH_3_), 3.75 (s, 6H, 2OCH_3_). ^13^C NMR (100 MHz, DMSO-d_6_) δ 167.64, 163.08, 159.18, 141.92, 129.84, 129.77, 129.61, 129.53, 128.68, 125.99, 123.77, 121.65, 115.58, 115.37, 108.61, 104.58, 90.84, 55.68, 55.58, 41.92. Anal. Calcd. for C_25_H_22_FNO_4_ (419.15): C, 71.59; H, 5.29; N, 3.34, Found: C, 71.61; H, 5.30; N, 3.35. ESI/MS: *m*/*z* Calcd. For [M + Na]^+^: 442.14, Found: 442.20.

Synthesis of (E)-1-benzyl-6-chloro-3-(2,4,6-trimethoxybenzylidene)indolin-2-one (**7d**).

Yellow liquid; 62% yield(123 mg); R_F_ = 0.34 (mobile phase is pet.ether:EtOAc, 1:1). ^1^H NMR (400 MHz, DMSO-d_6_) δ 7.61 (s, 1H, CH), 7.32–7.23 (m, 5H, Ar-H), 7.05 (d, *J* = 1.4 Hz, 1H, ind-7), 6.96 (dd, *J* = 8.2, 1.4 Hz, 1H, ind-5), 6.83 (d, *J* = 8.2 Hz, 1H, ind-4), 6.37 (s, 2H, Ar-H), 4.98 (s, 2H, CH_2_), 3.87 (s, 3H, OCH_3_), 3.77 (s, 6H, 2OCH_3_). ^13^C NMR (100 MHz, DMSO-d_6_) δ 167.78, 163.38, 159.28, 143.32, 136.58, 132.91, 129.05, 128.74, 127.49, 127.38, 125.04, 124.67, 121.35, 120.72, 108.86, 104.43, 90.90, 55.72, 55.62, 42.64. Anal. Calcd. for C_25_H_22_ClNO_4_ (435.12): C, 68.89; H, 5.09; N, 3.21, Found: C, 68.91; H, 5.10; N, 3.22. ESI/MS: *m*/*z* Calcd. For [M + Na]^+^: 458.11, Found: 458.20.

Synthesis of (E)-6-chloro-1-(4-chlorobenzyl)-3-(2,4,6-trimethoxybenzylidene) indolin-2-one (**7e**).

Yellow liquid; 80% yield(158 mg); R_F_ = 0.32 (mobile phase is pet.ether: EtOAc, 3:1). ^1^H NMR (400 MHz, DMSO-d_6_) δ 7.61 (s, 1H, CH), 7.42 (d, *J* = 8.6 Hz, 2H, Ar-H), 7.37 (d, *J* = 8.6 Hz, 2H, Ar-H), 7.08 (d, *J* = 1.9 Hz, 1H, ind-7), 6.97 (dd, *J* = 8.2, 1.9 Hz, 1H, ind-5), 6.83 (d, *J* = 8.2 Hz, 1H, ind-4), 6.37 (s, 2H, Ar-H), 4.97 (s, 2H, CH_2_), 3.87 (s, 3H, OCH_3_), 3.76 (s, 6H, 2OCH_3_). ^13^C NMR (100 MHz, DMSO-d_6_) δ 168.09, 153.41 (2C), 152.80, 144.57, 138.75, 134.57, 132.62, 129.69, 129.56, 129.18, 128.65, 124.83, 123.34, 122.27, 111.00, 110.07, 107.76, 60.69, 56.51, 42.52. Anal. Calcd. for C_25_H_21_Cl_2_NO_4_ (469.08): C, 63.84; H, 4.50; N, 2.98, Found: C, 63.86; H, 4.51; N, 2.99. ESI/MS: *m*/*z* Calcd. For [M + Na]^+^: 492.07, Found: 492.10.

Synthesis of (E)-6-chloro-1-(4-fluorobenzyl)-3-(2,4,6-trimethoxybenzylidene) indolin-2-one (**7f**).

Yellow liquid; 85% yield(160 mg); R_F_ = 0.4 (mobile phase is pet.ether:EtOAc, 3:1). ^1^H NMR (400 MHz, DMSO-d_6_) δ 7.68 (s, 1H, CH), 7.40 (s, 2H, Ar-H), 7.21 (d, *J* = 6.8 Hz, 2H, Ar-H), 7.17 (d, *J* = 6.8 Hz, 2H, Ar-H), 7.08 (d, *J* = 2.2 Hz, 1H, ind-7), 6.81 (dd, *J* = 8.2, 2.2 Hz, 1H, ind-5), 6.75 (d, *J* = 8.2 Hz, 1H, ind-4), 4.97 (s, 2H, CH_2_), 3.92 (s, 6H, 2OCH_3_), 3.77 (s, 3H, OCH_3_). ^13^C NMR (100 MHz, DMSO-d_6_) δ 167.61, 165.58, 152.95, 152.33, 144.14, 139.14, 138.22, 134.10, 129.51, 129.43, 124.41, 123.83, 121.74, 119.40, 115.42, 110.53, 107.29, 60.21, 56.03, 41.98. Anal. Calcd. for C_25_H_21_ClFNO_4_ (453.11): C, 66.16; H, 4.66; N, 3.09, Found: C, 66.17; H, 4.67; N, 3.10. ESI/MS: *m*/*z* Calcd. For [M-(4-Fluorbenzyl and 3 OCH_3_ groups)]^−^: 255.05, Found: 255.40.

#### 4.1.3. General Procedures for Synthesizing Compounds **9a,b**

A mixture of oxindole 3b (344 mg, 1 mmol) was stirred with K_2_CO_3_ (690 mg, 5 mmol) in acetonitrile (10 mL) for 30 min, the corresponding ester of 2-bromoacetate 9a or b (2 mmol) was then added portion-wise with KI (830 mg, 5 mmol). The mixture was then refluxed overnight. The product formation was traced by TLC. After reaction completion, the solvent was evaporated, the residue was extracted using ethyl acetate (3 × 30 mL (and washed with water (3 × 30 mL). The organic layer was collected and dried over anhydrous sodium sulphate, solvent was evaporated, and the obtained product [54] was purified by silica gel column using (petroleum ether, ethyl acetate, 7:3) as mobile phase.

Synthesis of t-Butyl (E)-2-(6-chloro-2-oxo-3-(3,4,5-trimethoxybenzylidene) indolin-1-yl)acetate (**9a**).

Yellow solid; 70% yield(143 mg); m.p. = 82–85 °C; R_F_ = 0.32 (mobile phase is pet.ether:EtOAc, 3:1). ^1^H NMR (400 MHz, DMSO-d_6_) δ 7.99 (s, 2H, Ar-H), 7.94 (s, 1H, CH), 7.78 (d, *J* = 8.3 Hz, 1H, ind-4), 7.20 (d, *J* = 2.0 Hz, 1H, ind-7), 7.14 (dd, *J* = 8.1, 2.0 Hz, 1H, ind-5), 4.57 (s, 2H, CH_2_), 3.85 (s, 6H, 2OCH_3_), 3.76 (s, 3H, OCH_3_), 1.42 (s, 9H, t-Bu). ^13^C NMR (100 MHz, DMSO-d_6_) δ 167.74, 167.01, 153.07, 152.45, 144.62, 138.16, 134.26, 129.23, 124.33, 123.83, 121.81, 119.27, 109.75, 107.41, 81.98, 60.33, 56.17, 42.04, 27.78. Calcd. for C_24_H_26_ClNO_6_ (459.14): C, 62.68; H, 5.70; N, 3.05, Found: C, 62.70; H, 5.71; N, 3.06. ESI/MS: *m*/*z* Calcd. For [M]^+^: 459.14, Found: 459.20.

Synthesis of Ethyl (E)-2-(6-chloro-2-oxo-3-(3,4,5-trimethoxybenzylidene) indolin-1-yl)acetate (**9b**).

Yellow solid; 70% yield(145 mg); m.p. = 60–62 °C; R_F_ = 0.3 (mobile phase is pet.ether:EtOAc, 3:1). ^1^H NMR (400 MHz, DMSO-d_6_) δ 7.98 (s, 2H, Ar-H), 7.92 (s, 1H, CH), 7.76 (d, *J* = 8.1 Hz, 1H, ind-4), 7.24 (d, *J* = 1.9 Hz, 1H, ind-7), 7.13 (dd, *J* = 8.1, 1.9 Hz, 1H, ind-5), 4.67 (s, 2H, CH_2_), 4.15 (q, *J* = 7.1 Hz, 2H, CH_2_-CH_3_), 3.84 (s, 6H, 2OCH_3_), 3.75 (s, 3H, OCH_3_), 1.21 (t, *J* = 7.1 Hz, 3H, CH_3_-CH_2_). ^13^C NMR (100 MHz, DMSO-d_6_) δ 168.09, 165.69, 152.45, 141.88, 140.55, 139.56, 133.05, 129.26, 123.13, 122.71, 121.80, 120.75, 110.59, 109.28, 61.27, 60.32, 56.07, 41.37, 14.13. Calcd. for C_22_H_22_ClNO_6_ (431.11): C, 61.19; H, 5.13; N, 3.24, Found: C, 61.21; H, 5.14; N, 3.25. ESI/MS: *m*/*z* Calcd. For [M]^+^: 431.11, Found: 431.10.

### 4.2. Biological Evaluation

#### 4.2.1. Two Dose Antiproliferative Activity Screening

a. Cell culture [79]

MCF-7(breast adenocarcinoma), PC-3 (prostate cancer) and COLO-205 (colorectal cancer) were obtained from Nawah Scientific inc., (Mokatam, Cairo, Egypt). Cells were maintained in Dulbecco’s Modified Eagle’s media (DMEM media) supplemented with 100 mg/mL of streptomycin, 100 units/mL of penicillin and 10% of heat-inactivated fetal bovine serum in humidified, 5% (*v*/*v*) CO_2_ atmosphere at 37 °C.

b. Cytotoxicity assay [79]

Cell viability was assessed by the sulforhodamine B (SRB) assay. Aliquots of cell suspension (5 × 10^3^ cells, 100 μL) were transferred into 96 well plates and incubated in complete media for 24 h. Cells were treated with another aliquot media (100 μL) containing compounds **3a–f, 4a–f, 6a–f, 7a–f, 9a–b** at concentrations of 1 and 10 μM). After 72 h of drug exposure, cells were fixed by replacing media with trichloroacetic acid (TCA, 10%, 150 μL) and incubated at 4 °C for 1 h. The TCA solution was removed, and the cells were washed five times with distilled water. Aliquots of SRB solution (0.4%w/v, 70 μL) were added and incubated in a dark place at room temperature for 10 min. Plates were washed three times with 1% acetic acid and allowed to air dry overnight. Then, a solution of TRIS base and TRIS HCl (TRIS buffer, 10 mM, 150 μL) was added to dissolve protein bound SRB stain. The absorbance was measured at 540 nm using BMGLABTECH^®^–FLUOstar omega micro-plate reader (Ortenberg, Germany).

#### 4.2.2. Antiproliferative Activity against COLO-205 and A549 Cell Lines

The same procedures used to screen two-dose anticancer activities were applied in determining IC_50_ values against COLO-205 using SRB assay. Cells were maintained at Roswell Park Memorial Institute media (RPMI media). The test was performed using 0.001–100 μM of compounds **4a** (**IC261**), **4b**, **4d, 4e**, **6e and 6f**. IC_50_ values were calculated using the equation for Boltzman sigmoidal concentration–response curve using the nonlinear regression fitting models by GraphPad, Prism version 5 (GraphPad Software Inc., La Jolla, CA, USA).

MTT assay for A549 cell line. Cell Line cells were obtained from American Type Culture Collection. Cells were cultured using DMEM (Invitrogen/Life Technologies, Carlsbad, CA, USA) supplemented with 10% FBS (Hyclone, Logan, UT, USA), 10 μg/mL of insulin (Sigma, St. Louis, MI, USA), and 1% penicillin-streptomycin. All the other chemicals and reagents were from Sigma (St. Louis, MI, USA) or Invitrogen (Carlsbad, CA, USA). Cells were plated (cells density 1.2–1.8 × 10,000 cells/well) in a volume of 100 µL complete growth medium + 100 μL of the tested compound per well in a 96-well plate for 24 h before the application of MTT assay protocol as reported, Supplementary Materials.

#### 4.2.3. Tubulin Polymerization Inhibitory Activity

The ability of compound **4b** to inhibit tubulin polymerization was detected using a fluorescent kit (cytoskeleton, Catalog number BK011P) and Tecan-spark reader according to prescribed manufacturer protocol (Danvers, MA, USA) [53]. A solution of compound **4b** in DMSO (10%, 5 μL) was added (in triplicate) to the wells of a 96-well plate. Then, 45 μL of a reaction mixture containing 2 mg/mL tubulin (>99% pure), PEM buffer (pH 6.9), piperazine-N,N’-bis(2-ethansulfonic acid) sequisodium salt (80 mM), EGTA (0.5 mM), MgCl_2_ (2 mM), GTP (1 mM), glycerol (10.2%), and 10 μM of fluorescent reporter, DAPI (4′,6-Diamidino-2 phenylindole) was warmed for 1 min at 37 °C before the addition of tubulin. Tubulin polymerization was determined by measuring the fluorescence emission at ƛ = 450 nm (excitation ƛ = 360 nm) for 60 min at min intervals. From resulting data IC_50_ for each compound were calculated using GraphPad Prism software (GraphPad Software Inc., La Jolla, CA, USA).

#### 4.2.4. Inhibition of EGFR Activity

The EGFR Kinase Assay Kit (Sigma Aldrich, St. Louis, MI, USA) is designed to measure EGFR Kinase activity for screening and profiling applications using Kinase-Glo^®^ MAX as a detection reagent [80]. The EGFR Kinase Assay Kit (Sigma Aldrich, St. Louis, MI, USA) comes in a convenient 96-well format, with enough purified recombinant EGFR enzyme, EGFR substrate, ATP, and kinase assay buffer for 100 enzyme reactions. The master mixture (25 μL per well) was prepared out of Kinase assay buffer (6 μL), ATP (500 μM, 1 μL), PTK substrate (50×, 1 μL) and water (17 μL) and added to each well. Inhibitor solution was made by dissolving 4b in a liquid labelled” inhibitor buffer”. An inhibitor solution (5 μL) was then added to each well, labelled as “Test Inhibitor”. For “Positive Control” and “Blank”, add 5 μL inhibitor buffer). EGFR was thawed on ice. Its tube was spun briefly to recover the full content of the tube. The enzyme was diluted with 1× Kinase assay buffer enzyme to 1 ng/μL. The reaction was initiated by adding 20 μL of diluted EGFR enzyme to the wells designated “Positive Control” and “Test Inhibitor Control”. The plate was incubated at 30 °C for 40 min. After the 40-min reaction, the Kinase-Glo Max (50 μL) reagent was added to each well. The plate was covered with aluminum foil, incubated at room temperature for 15 min and the luminescence was measured using the microplate reader.

#### 4.2.5. Inhibition of Casein Kinase (CK) Activity

The active CK1, kinase assay buffer, substrate solution and kinase dilution buffer were thawed on ice (^33^P-ATP assay Cocktail). In a precooled microcentrifuge tube, 10 μL of kinase solution, 5 μL of substrate solution and 5 μL of cold water (4 °C) were added, then a blank control was set up. Each reaction was initiated by adding 5 μL of ^33^P-ATP assay cocktail then incubated for 15 min in a water bath (30 °C), then 20 μL of the reaction mixture were spotted onto an individually precut strip of phosphocellulose P81 paper and allowed to air dry followed by washing using 1% H_2_SO_4_ with constant gentle stirring. A radioactive control was set up then 5 μL of γ-^33^P-ATP assay cocktail was spotted on P81 paper, the sample was allowed to air dry for 2 min then the radioactivity count was recorded.

#### 4.2.6. Cell Cycle Analysis

COLO-205 Cells were placed in a 6-well plate at 1 × 10^5^ concentration of cells/well then incubated for 24 h. COLO-205 Cells were treated with compound **4b** (2.34 μM) for 24 h. Cells were then collected and fixed for 12 h using ice cold 70% ethanol at 4 °C. Ethanol was then removed and cells were washed with cold phosphate buffer saline (PBS, 0.5 mL) and incubated for 30 min at 37 °C. The cells were stained for 30 min with propidium iodide in the dark. A flow cytometer was used to detect DNA content [81].

#### 4.2.7. Annexin V-FITC Assay

COLO-205 Cells were placed in a 6-well plate at 1 × 10^5^ concentration of cells/well then incubated for 24 h. COLO-205 Cells were treated with compound **4b** (2.34 μM) for 24 h. The cells were then harvested, washed with PBS and stained with Annexin V-FITC and PI in binding buffer (10 μM HEPES, 140 μM NaCl and 2.5 μM CaCl_2_ at pH 7.4) for 15 min at room temperature in the dark then analyzed by the flow cytometer [45,82].

#### 4.2.8. Bax, Bcl-2, Caspase 3 and Cytochrome C level Assay

a. Caspase 3 activation assay

The COLO-205 cell line was obtained from Nawah Scientific Inc., (Mokatam, Cairo, Egypt). RPMI 1640 containing 10% FBS was used to allow cells to grow at 37 °C, stimulated with compound **4b** (2.34 μM) to be tested for caspase 3 and lysed with cell extraction buffer. A standard diluent buffer was used to dilute the lysate over the range of the assay and measure human active caspase 3 content. Cells were plated at a density of 1.2–1.8 × 10,000 cells/well in a volume of 100 μL complete growth medium+ 100 μL of the tested compound (**4b**)/well in a 96-well plate for 24/48 h before the enzyme assay [73].

b. Bax and Bcl-2 assay

qRT-PCR was used to assess the protein expression of Bax and Bcl-2. The High Pure RNA Isolation Kit (RNeasy extraction kit, up to 1 × 10^7^ cells) was used to isolate total RNA from COLO-205 cells according to the manufacturer’s instructions. A Transcriptor First Strand c-DNA Synthesis Kit was used to produce c-DNA from RNA according to the manufacturer’s instructions; 10 μL total RNA was used for amplification Specific primers for Bax, Bcl-2, and P53 were used in PCR experiments. A LightCycler 480 PCR Master Mix was used to set up amplification reactions with a reaction volume of 20 uL. TaqMan probes and PCR primers were created and tuned. PCR Primer sequences were as following (Bax F 5′-ATGTTTTCTGACGGCAACTTC-3′, Bax R 5′-AGTCCAATGTCCAGCCCAT-3′. Bcl-2 F 5′-CCTGTGGATGACTGAGTACC-3′, and Bcl-2 R 5′-GAGACAGCCAGGAGAAATCA-3′) [70].

A dissociation assay was performed to evaluate any problem related to primer unspecific annealing or any secondary structure formation. Data were analyzed using SDS analysis software.

c. Cytochrome c assay

Cytochrome expression level was assessed using a Cytochrome c human ELISA kit-ab119521, Abcam^®^ (Cambridge, UK). The assay was done according to company protocols. The test started with precoating with biotin labelled cytochrome c specific antibodies. Standards and test samples were prepared according to the manual and added to individual wells of the pre-coated micro-plate. The microplate was then covered with adhesive film and incubated at room temperature for 2 h. Wells were then washed with wash buffer and a Streptavidin-HRP conjugate was added to each well and incubated at room temperature for one more hour. Unbound conjugates were then washed away using wash buffer. TMB substrate was then added and catalysed by HRP to produce a blue-coloured product. The enzyme reaction was stopped by the addition of a phosphoric acid stop solution, transforming the blue colour into yellow. The density of yellow coloration was measured spectrophotometrically at 450 nm and was directly proportional to the amount of Cytochrome c captured in the plate [83].

### 4.3. Docking Study

All the molecular modeling and visualization processes were performed within EGFR and tubulin active sites using Molecular Operating Environment (MOE) 2010.10 software. (Chemical Computing Group, Montreal, QC, Canada). The cocrystal structures were retrieved from the RCSB Protein Data Bank (PDB ID; 2ITO and 5LYJ).

First, the compounds were built with the standard tools designated in MOE 2010.10. The energy of the docked structures was minimized using an MMF94FX forcefield with a RMSD gradient of 0.05 kcal/mol, then the protein structure was prepared by using the MOE LigX protocol. To validate the docking study at the binding site, the native ligand gefitinib was self-docked into the binding site using the same set of parameters as described above. The RMSD of the best-docked pose was 1.3 Å and the energy score was −8.1787 kcal/mol, thus validating the docking using MOE. The ligands were then docked in the binding site using the Alpha triangle placement method. Refinement was carried out using Forcefield and scored using the Affinity dG scoring system. The resulting docking poses were visually inspected, and the pose of the lowest binding free energy value was considered.

### 4.4. Physicochemical, ADME and Pharmacokinetic Properties Prediction

The free SwissADME web tool available from the Swiss Institute of Bioinformatics (SIB, Lausanne, Switzerland) was used for the calculation of the physicochemical descriptors, as well as to predict the pharmacokinetic properties, ADME parameters, and drug-like nature of the most potent newly synthesized compounds (**4b** and **4e**) [47,48]. The structure of submitted compounds was converted to SMILES notations then submitted to the online server for calculation.

### 4.5. PgP Permeability Assay

PgP concentration was measured using ELISA kit Cat No: MBS722065) for human PgP assay. The Pgp ELISA kit employs a competitive enzyme immunoassay technique using a polyclonal anti-Pgp antibody and a PgpHRP conjugate. The test sample and buffer were incubated together with the PgpHRP conjugate on a precoated plate for one hour. After the incubation period, the wells were decanted and washed five times. The wells were then incubated with a substrate for the HRP enzyme. The enzyme-substrate reaction product formed a blue complex. Finally, a stop solution was added to stop the reaction, which then turned the solution yellow. Color intensity was measured spectrophotometrically at 450 nm in a microplate reader. The intensity of the color was inversely proportional to the Pgp concentration, since the Pgp of the samples and the PgpHRP conjugate competed for the binding site of the anti-Pgp antibody. Because the number of sites is limited, there were fewer sites left to bind to the PgpHRP conjugate as the sample Pgp occupies more sites. A standard curve was drawn that relates the intensity of the color to the concentration of the standards. The Pgp concentration in each sample was interpolated from this standard curve.

## 5. Conclusions

The current study sheds light upon 3-benzylideneindolin-2-one as a promising antiproliferative agent aiming at ambushing multiple cellular targets concomitantly. Compound **4b** induces cytotoxicity against the COLO-205 cell line. This cytotoxicity is associated with its ability to inhibit tubulin polymerization, causing a mitotic catastrophe. In addition, it can potentially inhibit multiple kinases, including EGFR and CK1. Collective data upheld its ability to prompt cell cycle arrest and subsequent apoptosis with a disruption in certain regulating proteins including Bax, Bcl-2, Caspase 3 and cytochrome C. These biological outcomes, along with the favorable physicochemical and pharmacokinetic properties, suggest **4b** as a potential lead in the design and development of new therapeutic agents for colon cancer.

## Figures and Tables

**Table 1:** Growth inhibition (%) of MCF-7, PC-3 and COLO-205 cancer cell lines treated with compounds **3a–f, 4a–f, 6a–f, 7a–f and 9a–b** at 1 and 10 μM doses using the SRB assay.

Compound Number	Growth Inhibition (%) ^a^											
	MCF-7				PC-3				COLO-205			
	1 μM		10 μM		1 μM		10 μM		1 μM		10 μM	
	Average	SEM	Average	SEM	Average	SEM	Average	SEM	Average	SEM	Average	SEM
**3a**	1.17	0.17	3.44	0.18	1.92	0.92	20.44	1.16	1.18	0.38	4.71	1.35
**3b**	1.14	0.3	41.17	0.75	7.69	1.33	38.99	0.44	5.70	0.80	32.58	1.32
**3c**	1.06	0.46	8.56	0.81	1.42	0.24	14.31	1.39	2.86	0.28	6.41	0.79
**3d**	0.98	0.31	3.60	1.00	2.02	0.30	1.91	0.07	7.01	1.00	11.85	0.18
**3e**	20.98	0.76	65.3 ^b^	0.67	32.94	0.07	61.38	0.69	2.34	0.33	18.61	1.52
**3f**	0.36	0.13	4.53	0.42	1.29	0.31	3.57	0.41	1.52	0.38	9.13	0.40
**4a**	43.49 ^c^	1.11	58.93	0.99	59.19	0.35	67.87	0.52	63.60	0.80	77.5	0.15
**4b**	45.3	1.23	59.15	0.95	64.05	1.19	71.66	1.54	73.32	1.07	82.5	1.23
**4c**	17.88	0.31	65.1	1.65	28.93	0.24	62.25	0.61	2.67	0.38	58.44	1.00
**4d**	10.02	0.60	66.11	0.33	23.81	0.14	57.98	0.89	9.69	0.51	64.01	1.07
**4e**	34.48	0.19	68.91	0.57	42.36	0.71	63.27	0.16	8.05	0.86	31.89	1.44
**4f**	11.49	0.13	65.85	1.13	23.02	0.25	69.12	1.14	7.64	0.66	63.79	0.66
**6a**	1.74	0.23	48.54	0.57	4.30	1.40	5.03	1.72	2.46	1.33	4.97	0.77
**6b**	1.13	0.89	32.21	0.47	1.04	0.14	28.88	0.67	3.90	0.90	45.84	0.87
**6c**	5.68	0.58	25.03	1.71	0.69	0.22	8.22	0.57	4.66	0.57	13.63	0.57
**6d**	0.68	0.29	48.38	1.08	4.24	0.30	7.34	0.34	2.38	0.92	8.68	0.21
**6e**	3.69	1.01	6.00	0.20	3.22	0.33	5.65	0.13	6.49	0.66	72.57	1.37
**6f**	3.56	0.88	29.87	0.61	0.40	0.17	23.37	0.99	3.67	1.14	63.77	0.87
**7a**	0.60	0.38	15.31	0.48	0.65	0.26	8.59	0.58	11.91	0.26	20.02	1.03
**7b**	3.81	0.98	5.48	0.48	1.05	0.11	3.15	1.21	3.00	1.19	3.33	0.33
**7c**	4.08	0.11	25.95	0.88	0.89	0.30	13.82	0.45	4.66	0.57	18.28	1.19
**7d**	0.49	0.23	40.55	0.90	1.47	0.25	19.9	0.99	2.38	1.06	15.56	1.19
**7e**	1.59	0.30	46.25	0.32	1.77	0.21	51.68	1.17	3.82	1.06	22.15	1.37
**7f**	10.27	0.58	15.96	0.71	0.75	0.05	7.08	0.17	3.33	0.33	3.97	0.65
**9a**	2.11	1.07	7.11	0.64	1.19	0.15	5.56	0.75	3.05	1.32	12.98	1.66
**9b**	2.30	1.11	6.82	0.18	1.66	0.41	13.20	0.49	35.14	1.75	40.87	0.66

^a^ Measured as triplicates and listed as average ± standard error of mean (SEM). ^b^ GI inhibition of 40–60% is marked in grey ^c^ GI inhibition more than 60% is marked in green.

**Table 2:** IC_50_ of compounds **4a** (**IC261**), **4b**, **4d**, **4e**, **6e** and **6f** against COLO-205 using SRB assay and IC_50_ of compounds **4a** (**IC261**), **4b** against cell line using MTT assay.

Compound	Cell Line	4a (IC261)	4b	4d	4e	6e	6f
IC_50_ (μM)	COLO-205	0.20	0.20	1.00	0.30	15.30	10.60
	A549	28.4	9.5	-	-	-	-

**Table 3:** Tubulin polymerization inhibitory activity (IC_50_, μM) for compound **4b** compared to CA-4, CK1 inhibitory activity (IC_50_, μM) of compounds **4b** and **4e** compared to compound **4a (**IC261**),** and the EGFR inhibitory activity (IC_50_, μg/mL) of compounds **4b** and **4e** compared to Geftinib.

Compound Number	Tubulin Polymerization Inhibition (IC_50_, μM)		Casein Kinase I Inhibition (IC_50_, μg/mL)		EGFR Inhibition (IC_50_, μg/mL)	
	Average	±SD	Average	±SD	Average	±SD
**4a**	5.045	0.31	2.39	0.12	0.106	0.002
**4b**	1.66	0.08	1.92	0.09	0.19	0.004
**4e**	-	-	13.08	0.66	0.401	0.008
Standard	0.42 (CA-4)	0.02	2.39 (4a, **IC261**)	0.12	0.057 (Gefitinib)	0.001

**Table 4:** DNA content in the cell cycle of the COLO-205 cancer cell line after treatment with **4b** and DMSO control, and the effect of compound **4b** on the percentage accumulation of cells at phases of cell cycle and on distribution of apoptotic cells in the annexin V-FITC experiment of the COLO-205 cancer cell line.

Sample	Results DNA Content			
	%G0-G1	%S	%G2/M	%Pre-G1
4b/Colo-205	47.15	49.91	2.94 *	41.28 *
DMSO/Colo 205(control)	41.64	51.39	6.97	2.43
**Sample**	**Apoptosis**			**Necrosis**
	Total	Early	Late	
4b/Colo-205	41.28	2.61	21.31	17.36

* Data obtained from this group are significantly different from the control with two-tailed *p* value less than 0.0001.

**Table 5:** Docking energy scores (S) in kcal/mol, interacting amino acid, distance in Å of the tested compound (**4a, 4b, 4d, 4e** and **CA-4**), and RMSD_Refine assessed upon docking compounds **4a–e** and **CA-4** at the colchicine binding site of tubulin (PDB code: 5LYJ).

Compound	S Score ^a^ Kcal/mol	Amino Acid/Bond	Distance (Å)	RMSD_Refine ^b^
**4a**	−7.9046	Cys241/H-donor	3.62	1.6019
		Thr179/H-donor	2.91	
**4b**	−8.2658	Cys241/H-donor	3.62	1.22
		Thr179/H-donor	2.91	
**4d**	−7.9163	Cys241/H-donor	3.63	1.1067
		Thr179/H-donor	2.88	
**4e**	−8.0707	Cys241/H-donor	2.75	1.5655
		Thr179/H-donor	3.56	
CA-4	−8.8842	Cys241/H-donor	3.72	1.3880
		Thr179/H-donor	3.00	
		Met259/H-donor	3.55	

^a^ S: The score of placements of a compound into the binding pocket of protein using the London dG scoring function; ^b^ RMSD_Refine: the root-mean-squared-deviation (RMSD) between the heavy atoms of the predicted pose (after refinement) and those of the crystal structure (before refinement).

**Table 6:** Concentration of Pgp measured in ng/mL in MCF-7 breast cancer cells treated with **4b** or ketokonazole.

Compound No	Concentration (ng/mL)	
	Average	SEM
**Control**	387.1	5.87
**4b**	242.0	3.08
**Ketokonazole**	189.8	2.34

## Data Availability

Data is contained within the article and Supplementary Materials.

## Supplementary Materials

The following are available online at https://www.mdpi.com/article/10.3390/ph14111114/s1.
