# Supplementary material for: New Multi-Targeted Antiproliferative Agents: Design and Synthesis of IC261-Based Oxindoles as Potential Tubulin, CK1 and EGFR Inhibitors
Source: Pharmaceuticals (Basel). 2021 Oct 30;14(11):1114. doi: 10.3390/ph14111114 (PMC8620390; doi:10.3390/ph14111114)
Supplement: Supplementary file 1 [file pharmaceuticals-14-01114-s001.zip › pharmaceuticals-1389599-supplementary.pdf]

List of contents

Ser.	Content	Page no.
1	**Figure S1**. LC/MS spectrum of compound **3b**.	5
2	**Figure S2**. ^{1}H-NMR spectrum of compound **4b** in DMSO-*d6* at 400MHz.	6
3	**Figure S3**. ^{13}C-NMR spectrum of compound **4b** in DMSO-*d6* at 100MHz.	6
4	**Figure S4**. LC/MS spectrum of compound **4b**	7
5	**Figure S5**. LC/MS spectrum of compound **4c.**	7
6	**Figure S6**. ^{1}H-NMR spectrum of compound **4d** in DMSO-*d6* at 400MHz.	8
7	**Figure S7**. ^{13}C-NMR spectrum of compound **4d** in DMSO-*d6* at 100MHz.	9
8	**Figure S8**. LC/MS spectrum of compound **4d**	10
9	**Figure S9**. ^{1}H-NMR spectrum of compound **4e** in DMSO-*d6* at 400MHz.	10
10	**Figure S10**. ^{13}C-NMR spectrum of compound **4e** in DMSO-*d6* at 100MHz.	11
11	**Figure S11**. LC/MS spectrum of compound **4e**	12
12	**Figure S12**. ^{1}H-NMR spectrum of compound **6a** in DMSO-*d6* at 400MHz.	12
13	**Figure S13**. ^{13}C-NMR spectrum of compound **6a** in DMSO-*d6* at 100MHz.	13
14	**Figure S14**. LC/MS spectrum of compound **6a**	14
15	**Figure S15**. LC/MS spectrum of compound **6b**	14
16	**Figure S16**. ^{1}H-NMR spectrum of compound **6c** in DMSO-*d6* at 400MHz.	15
17	**Figure S17**. ^{13}C-NMR spectrum of compound **6c** in DMSO-*d6* at 100MHz.	16
18	**Figure S18**. LC/MS spectrum of compound **6c**	17
19	**Figure S19**. ^{1}H-NMR spectrum of compound **6d** in DMSO-*d6* at 400MHz.	18
20	**Figure S20**. ^{13}C-NMR spectrum of compound **6d** in DMSO-*d6* at 100MHz.	19

21	**Figure S21**. LC/MS spectrum of compound **6d.**	19
22	**Figure S22**. ^{1}H-NMR spectrum of compound **6e** in DMSO-*d6* at 400MHz.	20
23	**Figure S23**. ^{13}C-NMR spectrum of compound **6e** in DMSO-*d6* at 100MHz.	20
24	**Figure S24**. LC/MS spectrum of compound **6e.**	21
25	**Figure S25**. ^{1}H-NMR spectrum of compound **6f** in DMSO-*d6* at 400MHz.	22
26	**Figure S26**. ^{13}C-NMR spectrum of compound **6f** in DMSO-*d6* at 100MHz.	23
27	**Figure S27**. LC/MS spectrum of compound **6f.**	23
28	**Figure S28**. ^{1}H-NMR spectrum of compound **7a** in DMSO-*d6* at 400MHz.	24
29	**Figure S29**. ^{13}C-NMR spectrum of compound **7a** in DMSO-*d6* at 100MHz.	24
30	**Figure S30**. LC/MS spectrum of compound **7a.**	25
31	**Figure S31**. ^{1}H-NMR spectrum of compound **7b** in DMSO-*d6* at 400MHz.	25
32	**Figure S32**. ^{13}C-NMR spectrum of compound **7b** in DMSO-*d6* at 100MHz.	26
33	**Figure S33**. LC/MS spectrum of compound **7b.**	27
34	**Figure S34**. ^{1}H-NMR spectrum of compound **7c** in DMSO-*d6* at 400MHz.	28
35	**Figure S35**. ^{13}C-NMR spectrum of compound **7c** in DMSO-*d6* at 100MHz.	28
36	**Figure S36**. LC/MS spectrum of compound **7c.**	29
37	**Figure S37**. ^{1}H-NMR spectrum of compound **7d** in DMSO-*d6* at 400MHz.	29
38	**Figure S38**. ^{13}C-NMR spectrum of compound **7d** in DMSO-*d6* at 100MHz.	30
39	**Figure S39**. LC/MS spectrum of compound **7d.**	30

40	**Figure S40**. ^{1}H-NMR spectrum of compound **7e** in DMSO-*d6* at 400MHz.	31
41	**Figure S41**. ^{13}C-NMR spectrum of compound **7e** in DMSO-*d6* at 100MHz.	31
42	**Figure S42**. LC/MS spectrum of compound **7e.**	32
43	**Figure S43**. ^{1}H-NMR spectrum of compound **7f** in DMSO-*d6* at 400MHz.	32
44	**Figure S44**. ^{13}C-NMR spectrum of compound **7f** in DMSO-*d6* at 100MHz.	33
45	**Figure S45**. LC/MS spectrum of compound **7f.**	34
46	**Figure S46**. ^{1}H-NMR spectrum of compound **9a** in DMSO-*d6* at 400MHz.	34
47	**Figure S47**. ^{13}C-NMR spectrum of compound **9a** in DMSO-*d6* at 100MHz.	35
48	**Figure S48**. LC/MS spectrum of compound **9a.**	35
49	**Figure S49**. ^{1}H-NMR spectrum of compound **9b** in DMSO-*d6* at 400MHz.	36
50	**Figure S50**. ^{13}C-NMR spectrum of compound **9b** in DMSO-*d6* at 100MHz.	36
51	**Figure S51**. LC/MS spectrum of compound **9b.**	37
52	**Figure S52**. Tubulin polymerization assay for compound **4b**	37
53	**Figure S53**. Tubulin polymerization assay for compound **4a**	38
54	**Figure S54**. Epidermal growth factor receptor (EGFR) inhibition assay for compound **4b**	38
55	**Figure S55**. Epidermal growth factor receptor (EGFR) inhibition assay for compound **4a**	39
56	**Figure S56**. Casein kinase I (CK1) inhibition assay	40
57	**Figure S57**. P-gp substrate assay.	41
58	**Figure S58**. IC_{50} of compounds **4a (IC261), 4b, 4d, 4e, 6e** and **6f** ag**ainst COLO-205 using SRB assay.**	42
59	**Figure S59**. Cytotoxicity assay and IC_{50} of compounds **4a, 4b** and **5-FU** ag**ainst A549 cell line**	43
60	**Cell culture protocol**	44
61	**Figure S60**. Raw data of cytotoxicity assay and IC_{50} of compounds **4a, 4b** and **5-FU** ag**ainst A549 cell line.**	46
62	**Figure S61**. Elemental analysis data**.**	47

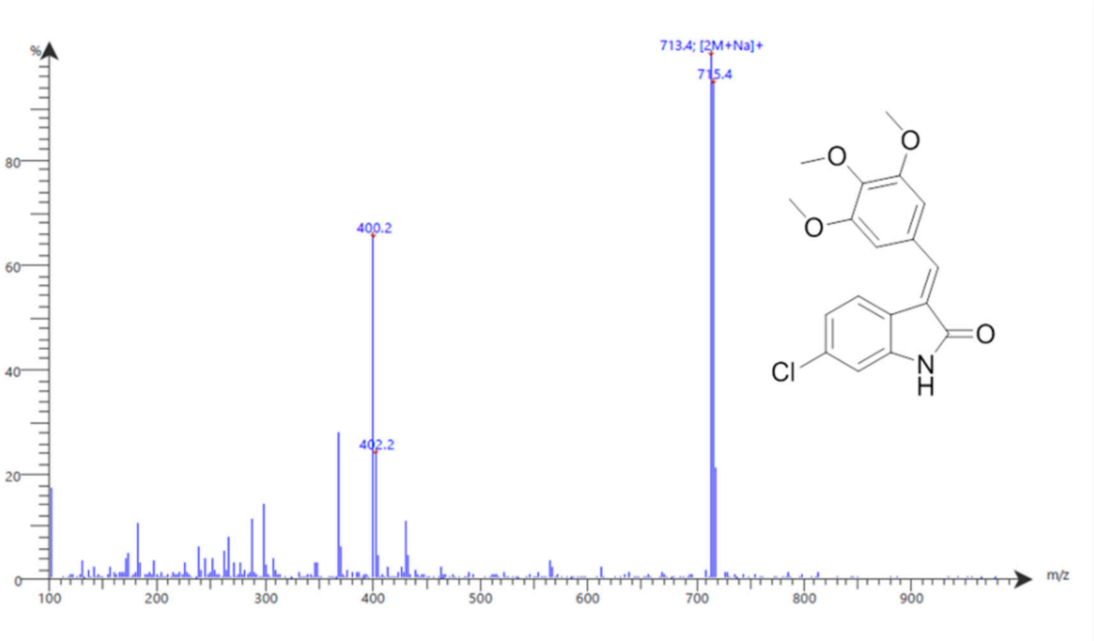

Figure S1. LC/MS spectrum of compound **3b**.

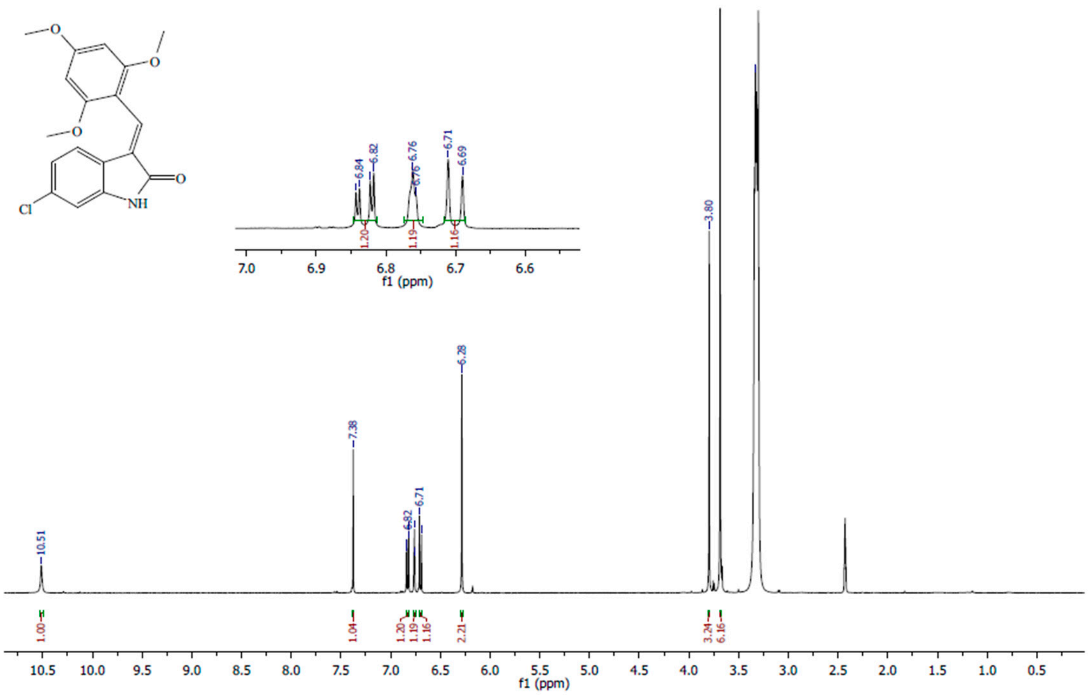

Figure S2. ^{1}H-NMR spectrum of compound **4b** in DMSO-*d6* at 400MHz.

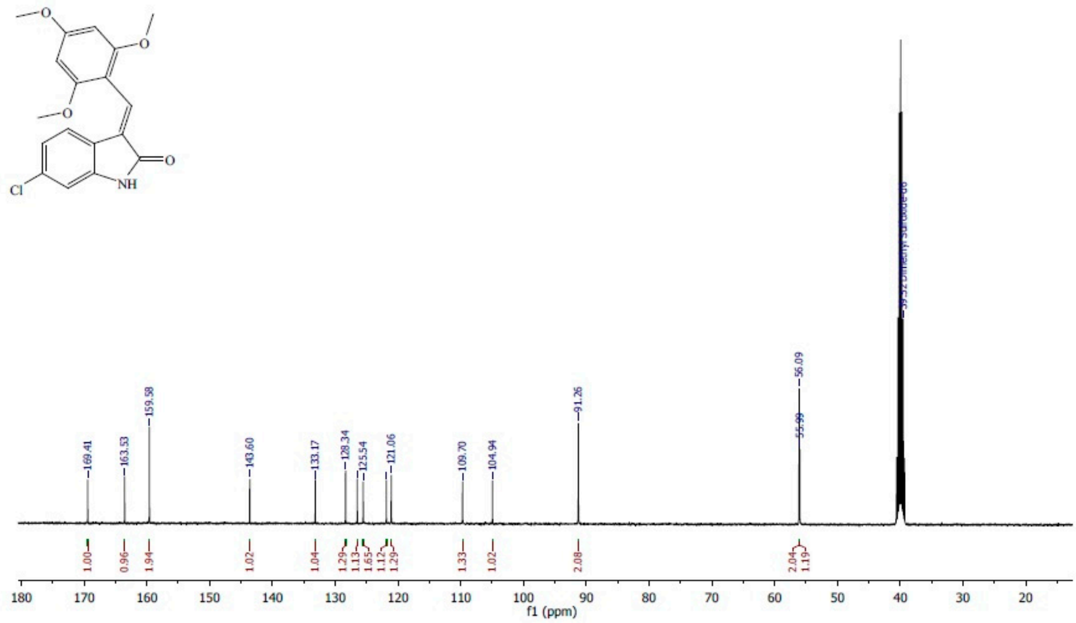

Figure S3. ^{13}C-NMR spectrum of compound **4b** in DMSO-*d6* at 100MHz.

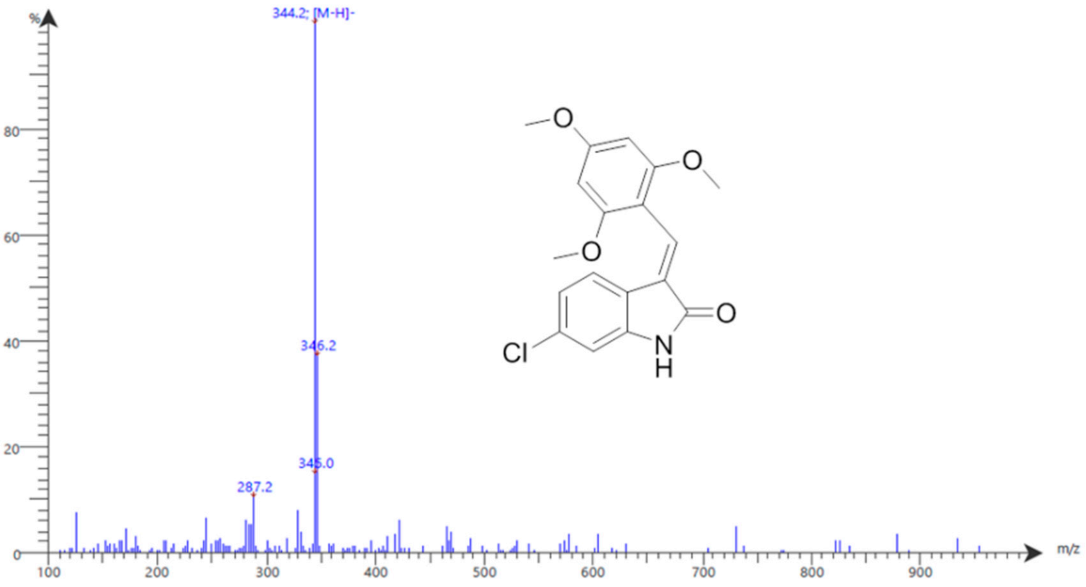

Figure S4. LC/MS spectrum of compound **4b**.

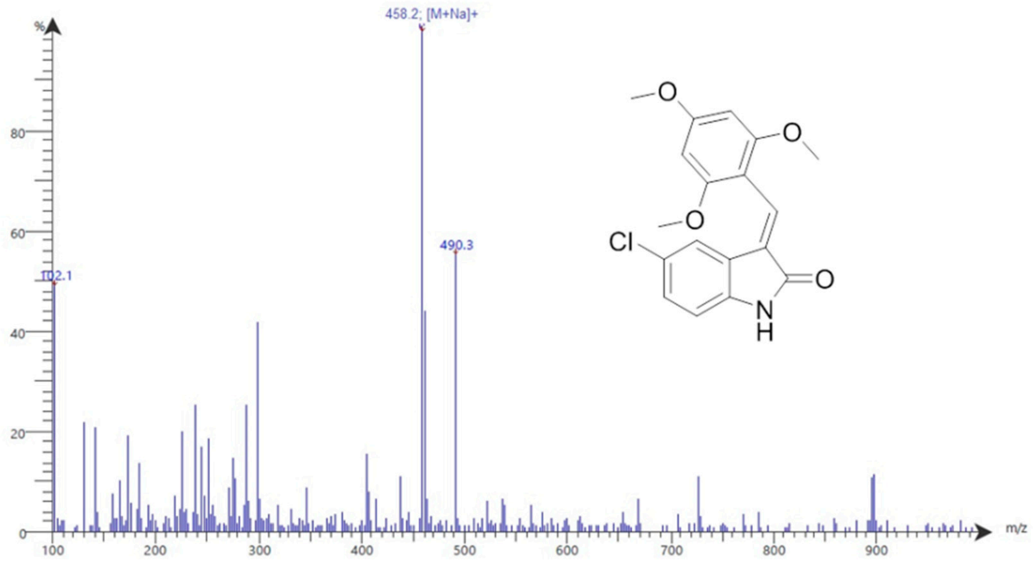

Figure S5. LC/MS spectrum of compound **4c.**

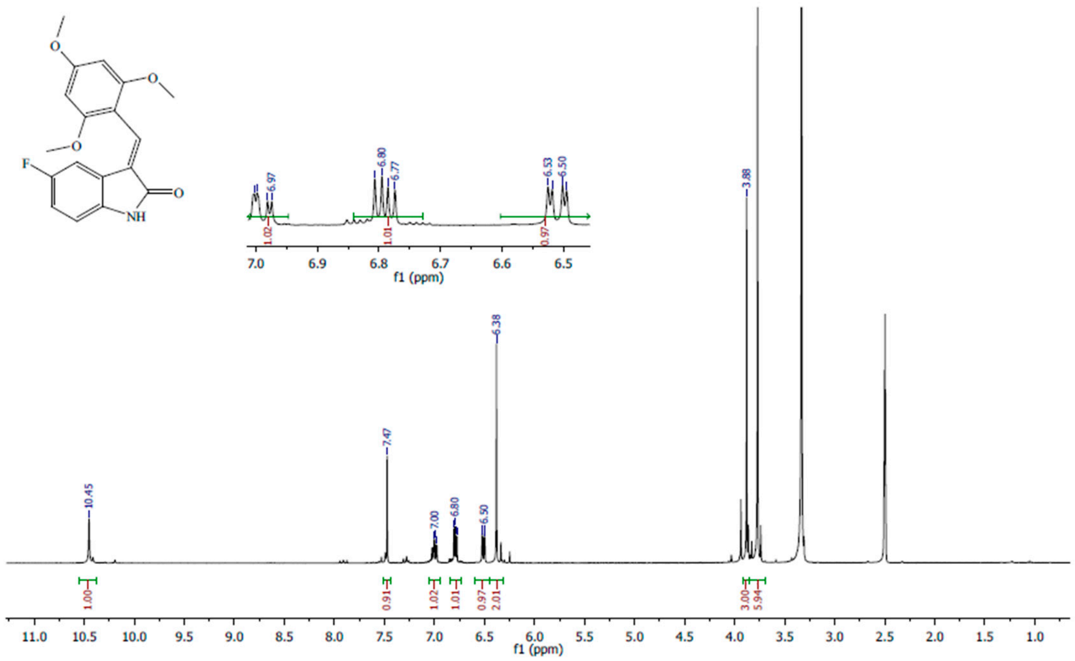

Figure S6. ^{1}H-NMR spectrum of compound **4d** in DMSO-*d6* at 500MHz.

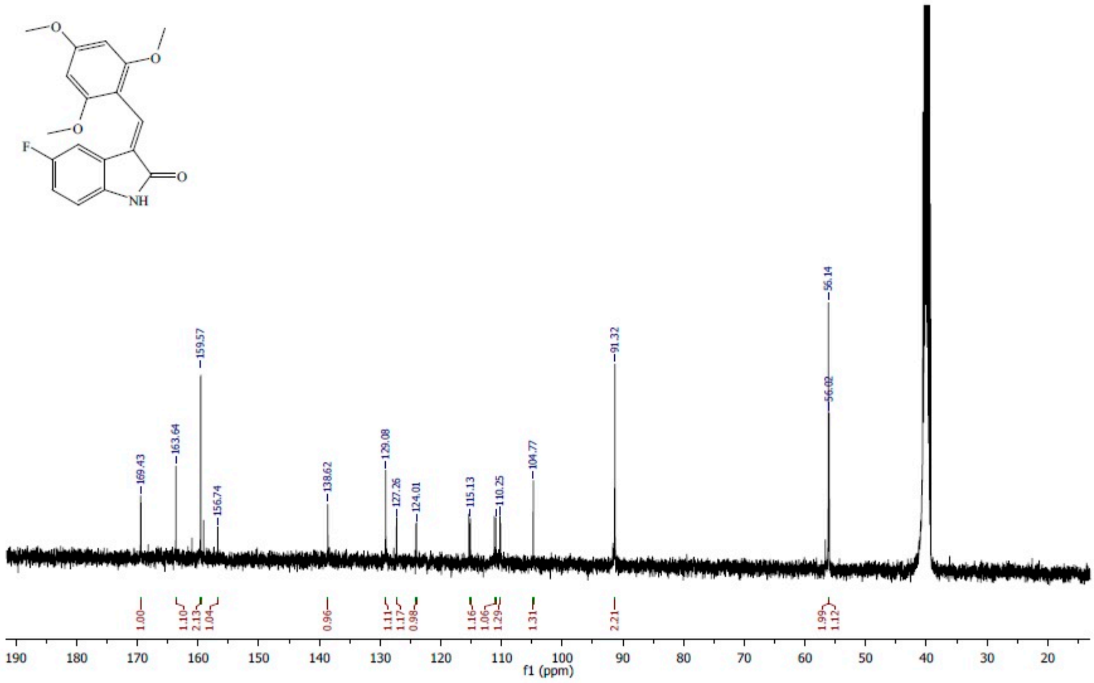

Figure S7. ^{13}C-NMR spectrum of compound **4d** in DMSO-*d6* at 100MHz.

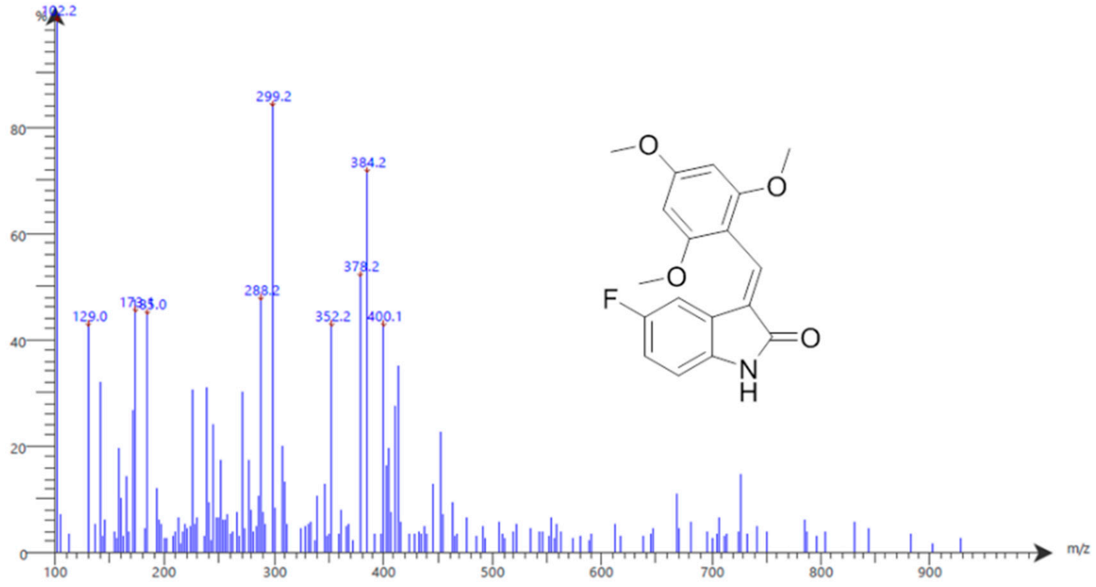

Figure S8. LC/MS spectrum of compound **4d**.

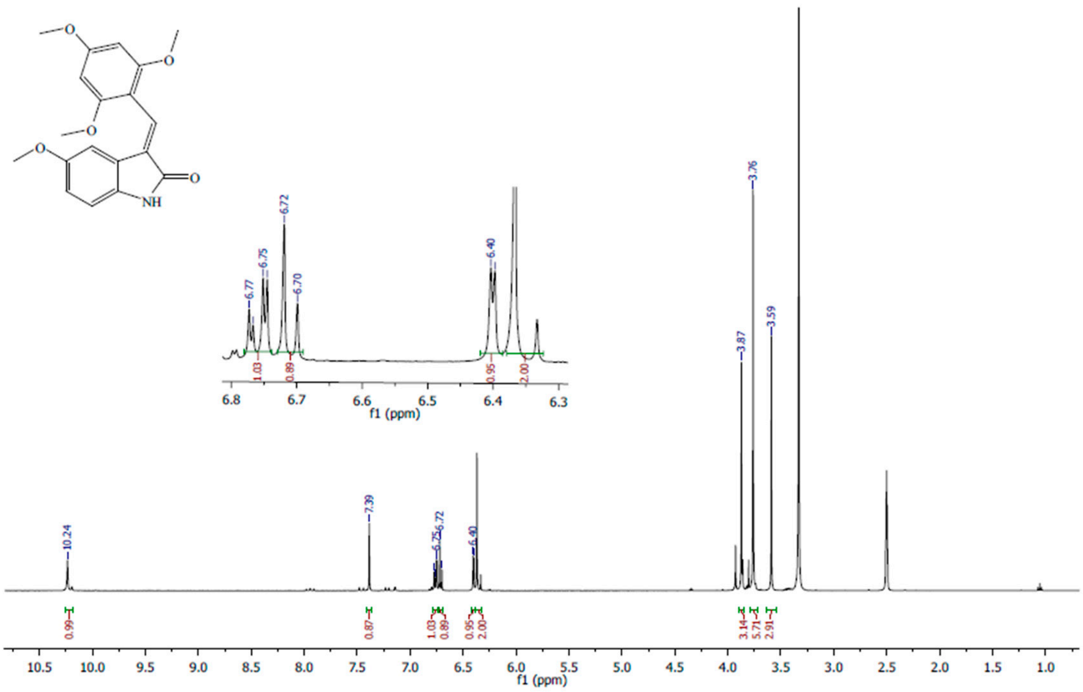

Figure S9. ^{1}H-NMR spectrum of compound **4e** in DMSO-*d6* at 400MHz.

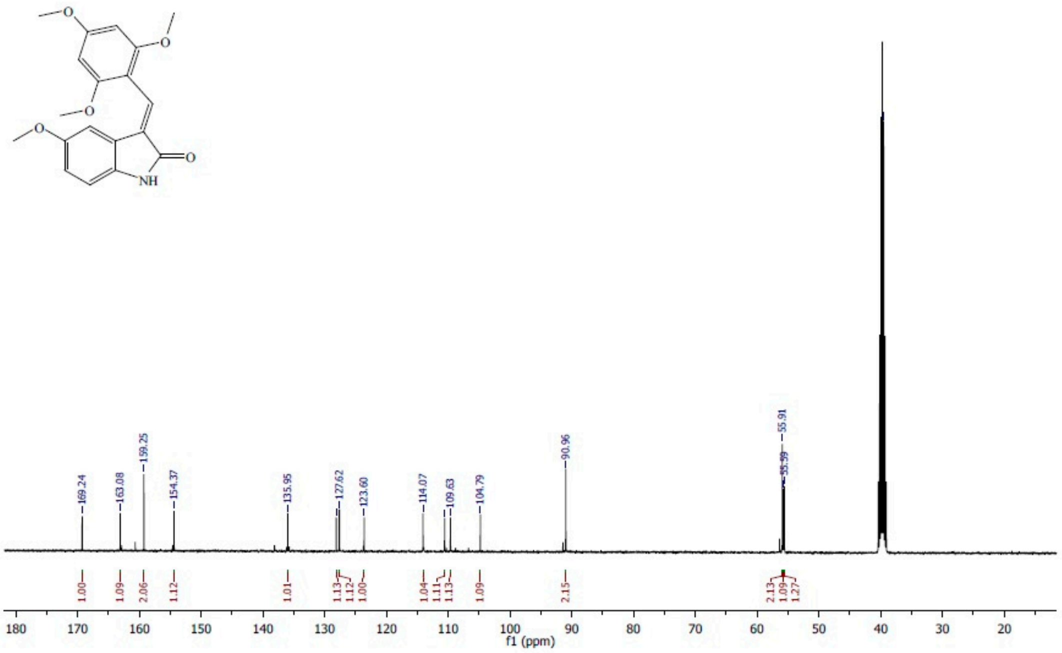

Figure S10. ^{13}C-NMR spectrum of compound **4e** in DMSO-*d6* at 100MHz.

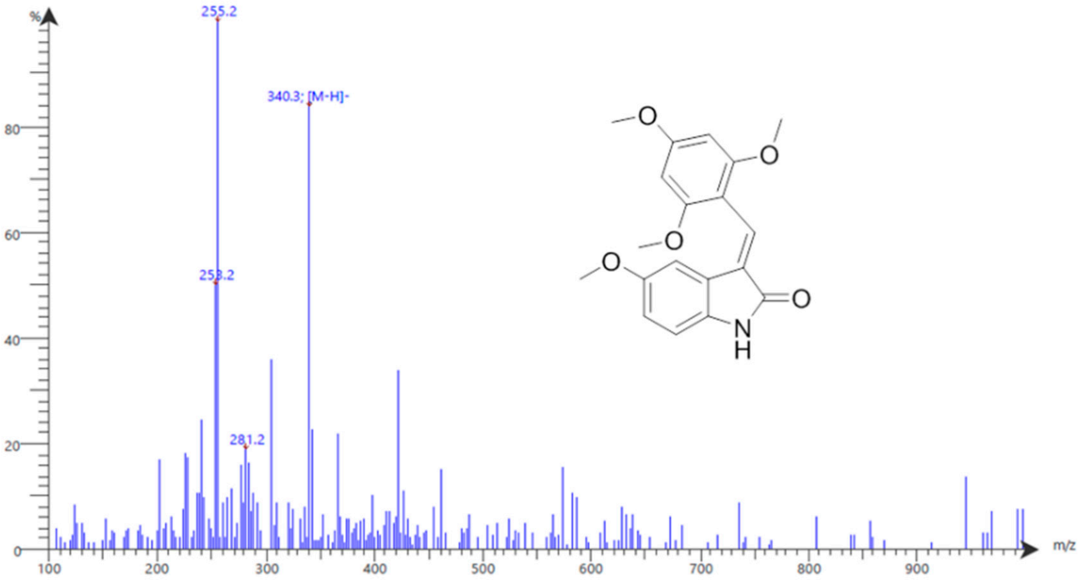

Figure S11. LC/MS spectrum of compound **4e.**

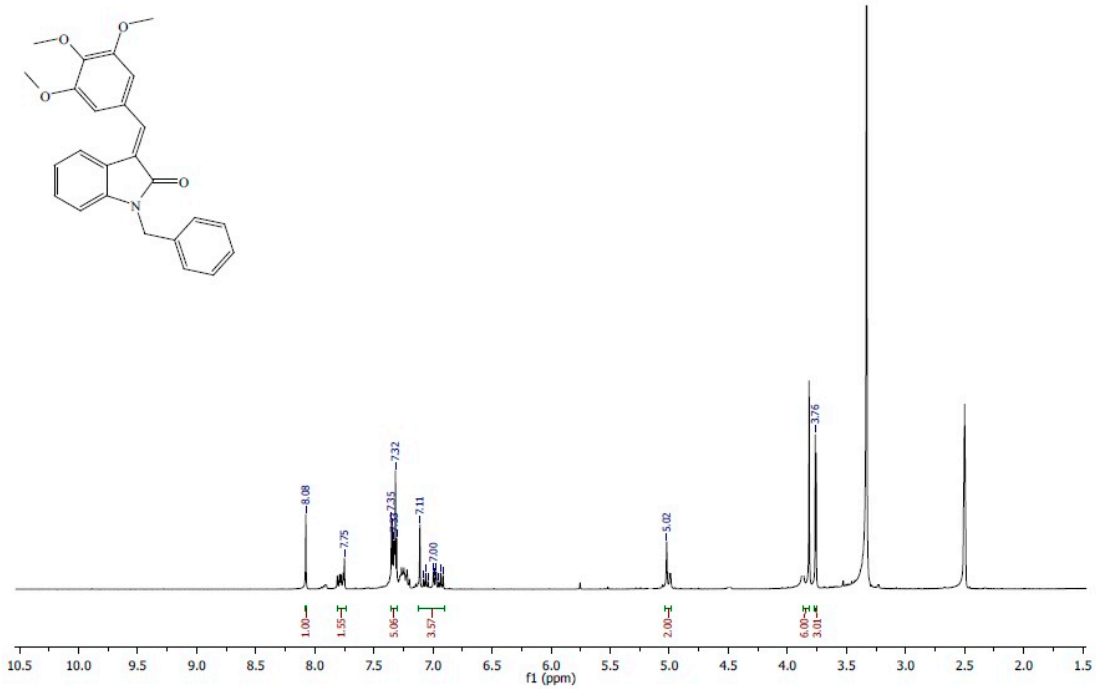

Figure S12. ^{1}H-NMR spectrum of compound **6a** in DMSO-*d6* at 400MHz.

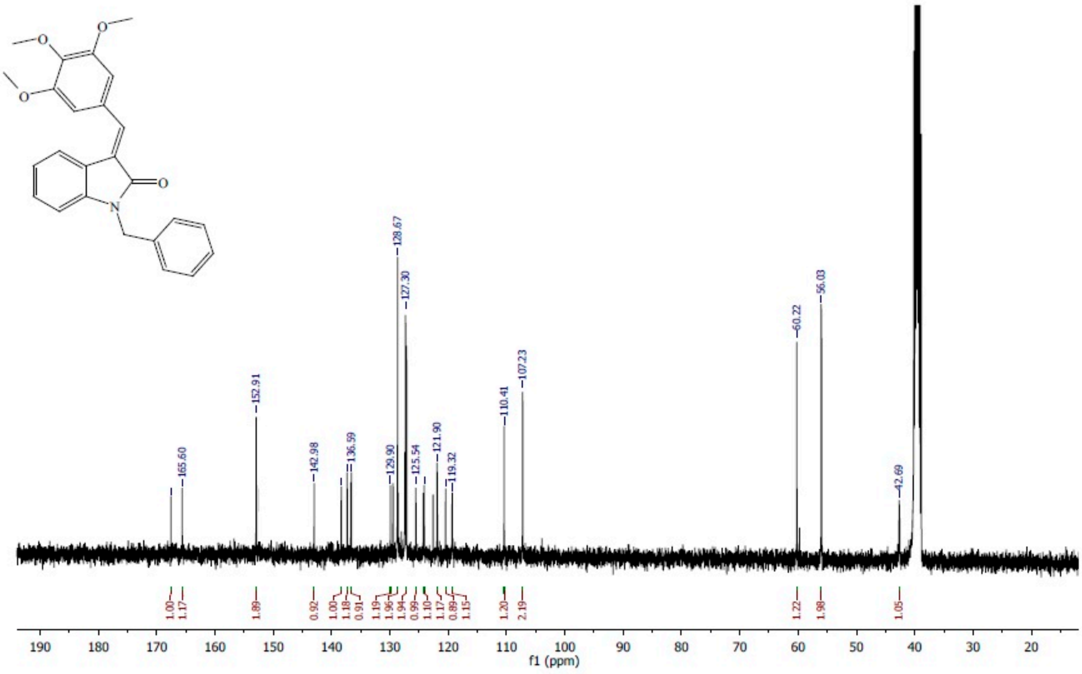

Figure S13. ^{13}C-NMR spectrum of compound **6a** in DMSO-*d6* at 100MHz.

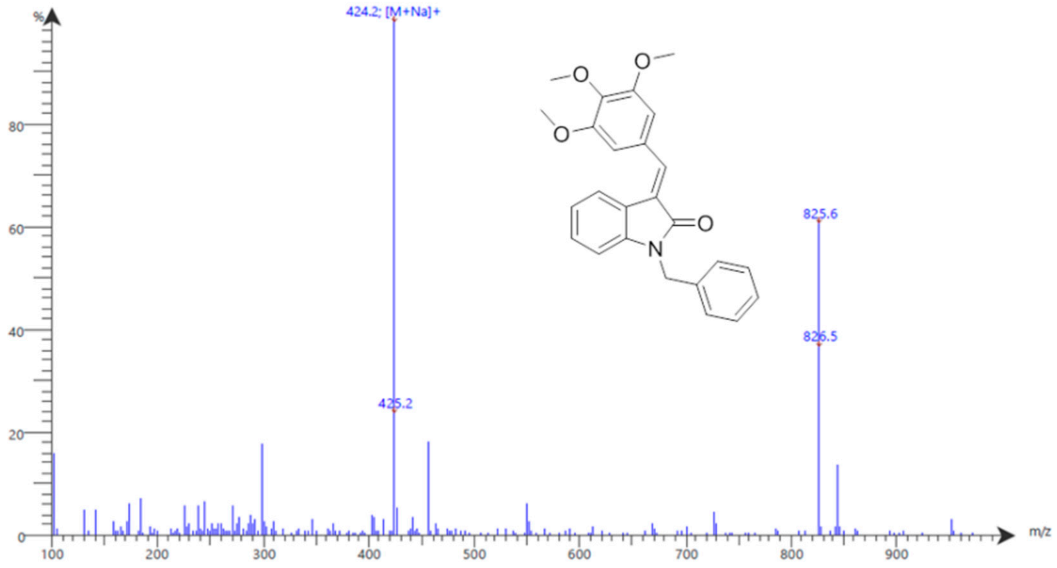

Figure S14. LC/MS spectrum of compound **6a.**

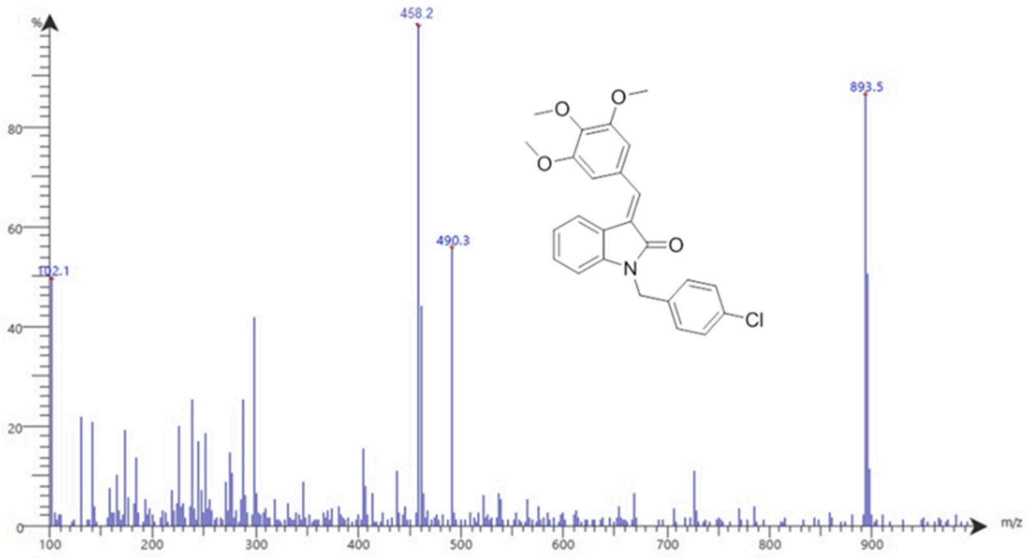

Figure S15. LC/MS spectrum of compound **6b**.

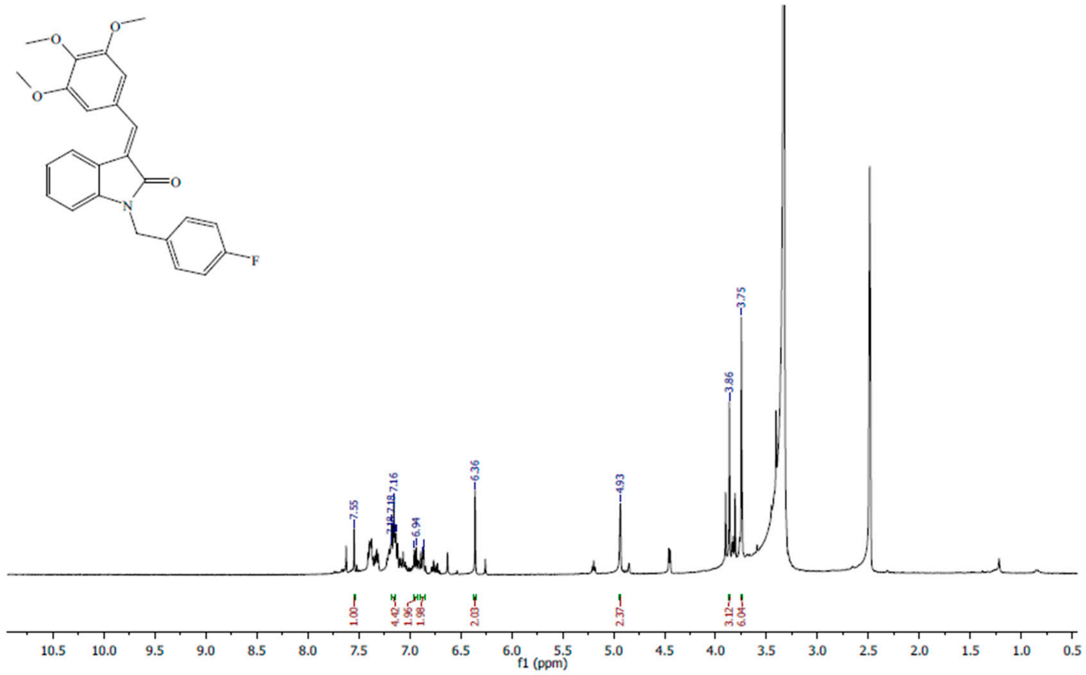

Figure S16. ^{1}H-NMR spectrum of compound **6c** in DMSO-*d6* at 400MHz.

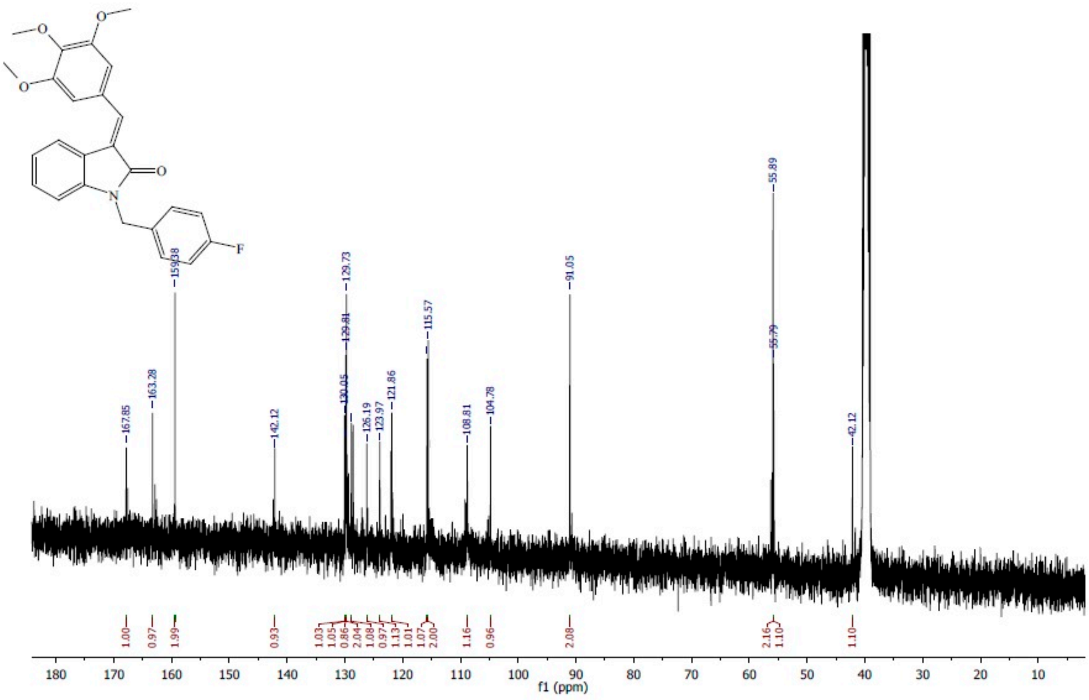

Figure S17. ^{13}C-NMR spectrum of compound **6c** in DMSO-*d6* at 100MHz.

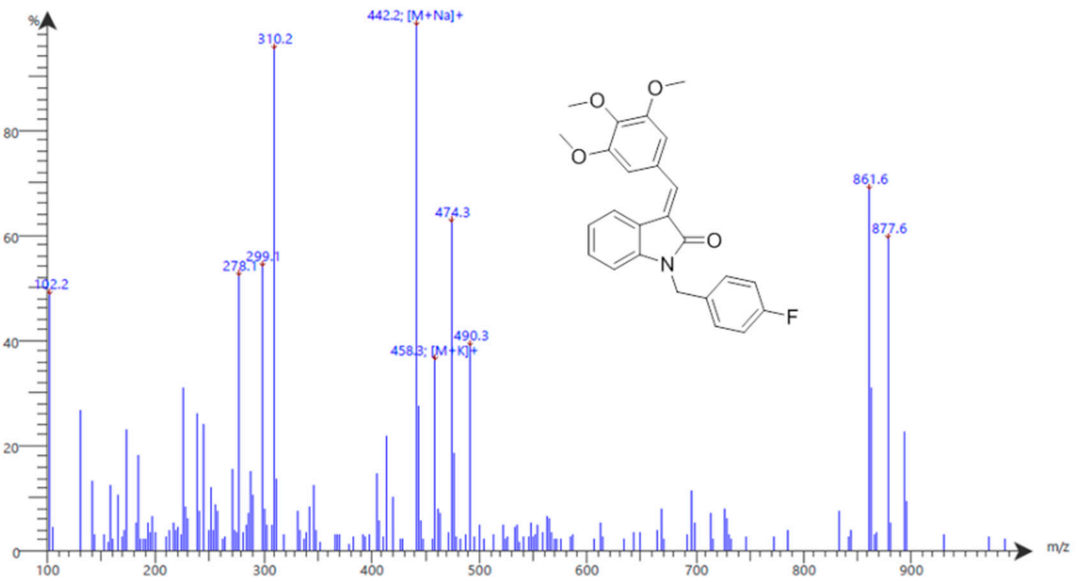

Figure S18. LC/MS spectrum of compound **6c**.

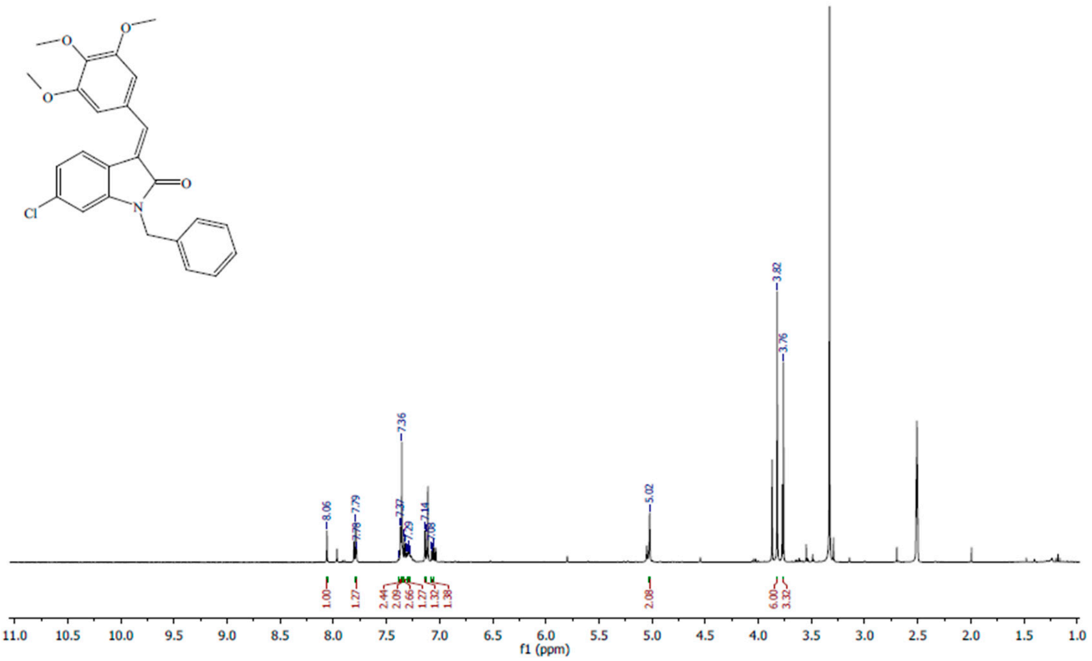

Figure S19. ^{1}H-NMR spectrum of compound **6d** in DMSO-*d6* at 400MHz.

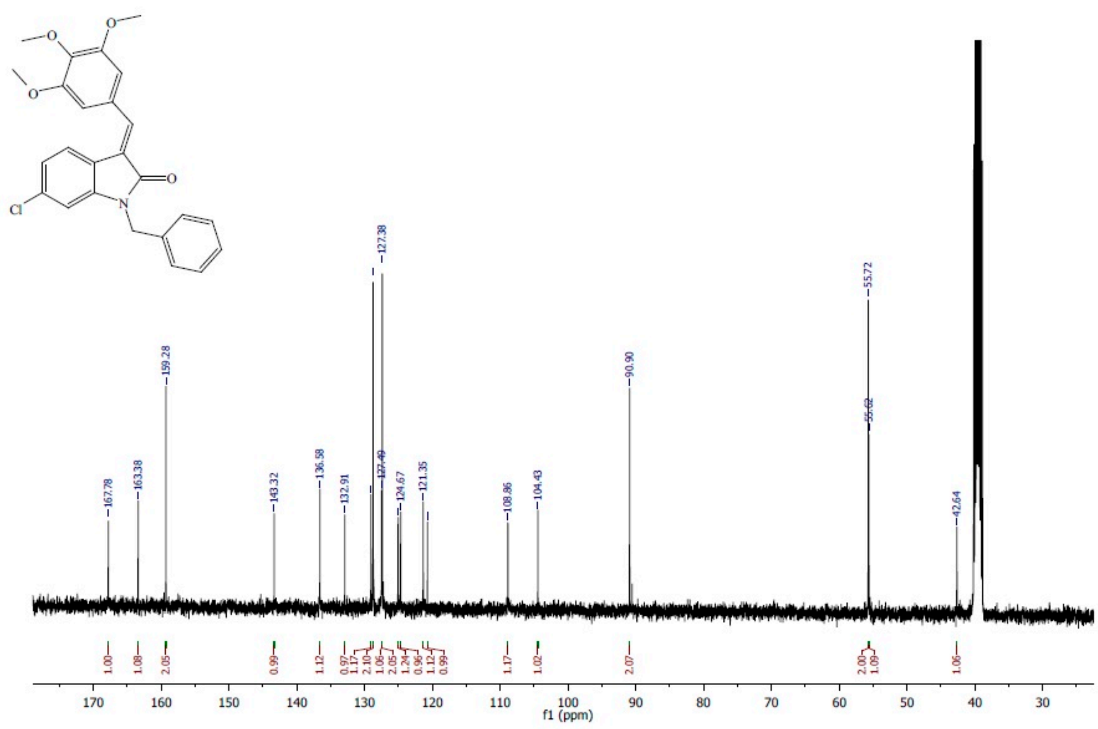

Figure S20. ^{13}C-NMR spectrum of compound **6d** in DMSO-*d6* at 100MHz.

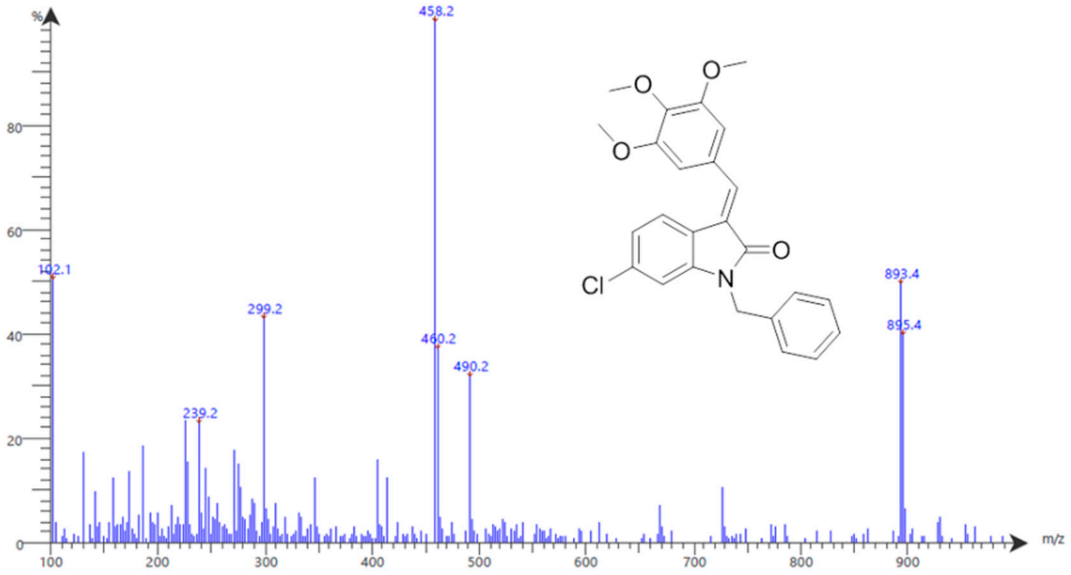

Figure S21. LC/MS spectrum of compound **6d.**

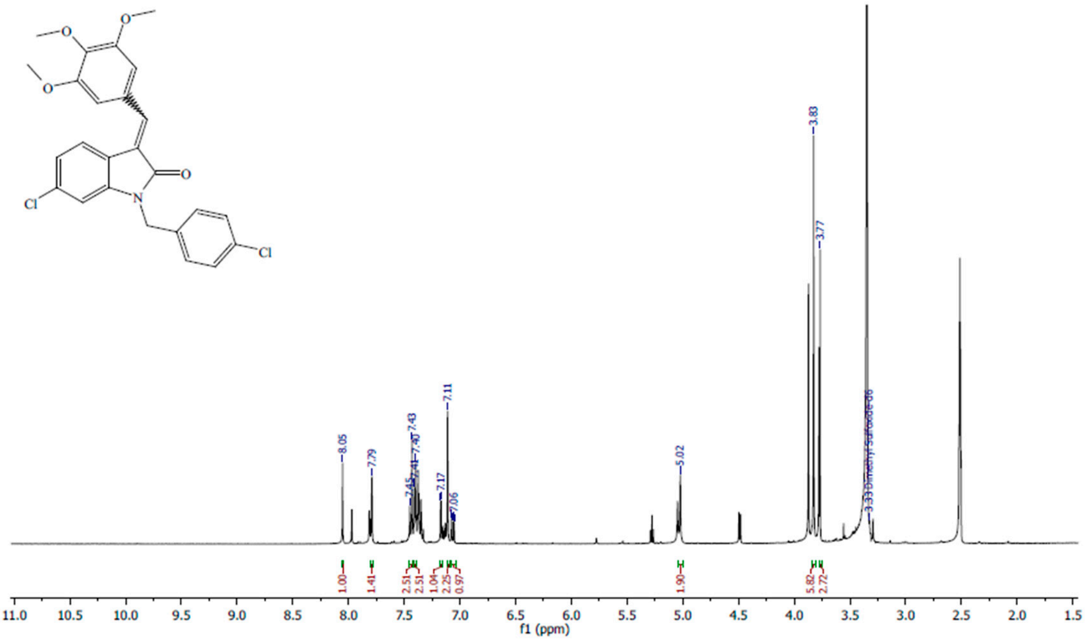

Figure S22. ^{1}H-NMR spectrum of compound **6e** in DMSO-*d6* at 400MHz.

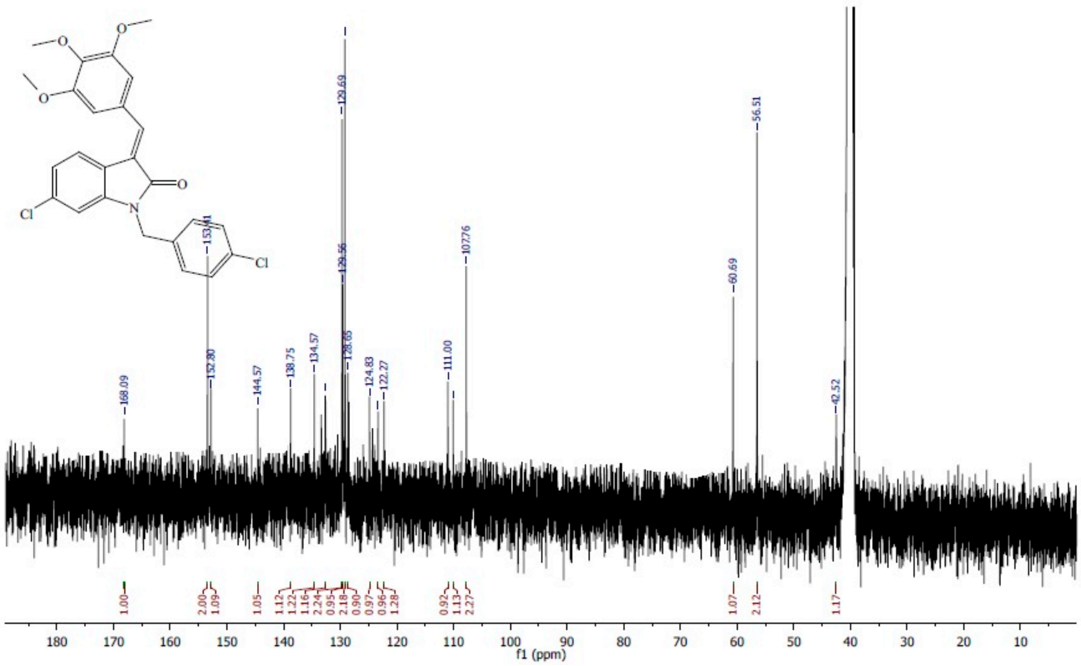

Figure S23. ^{13}C-NMR spectrum of compound **6e** in DMSO-*d6* at 100MHz.

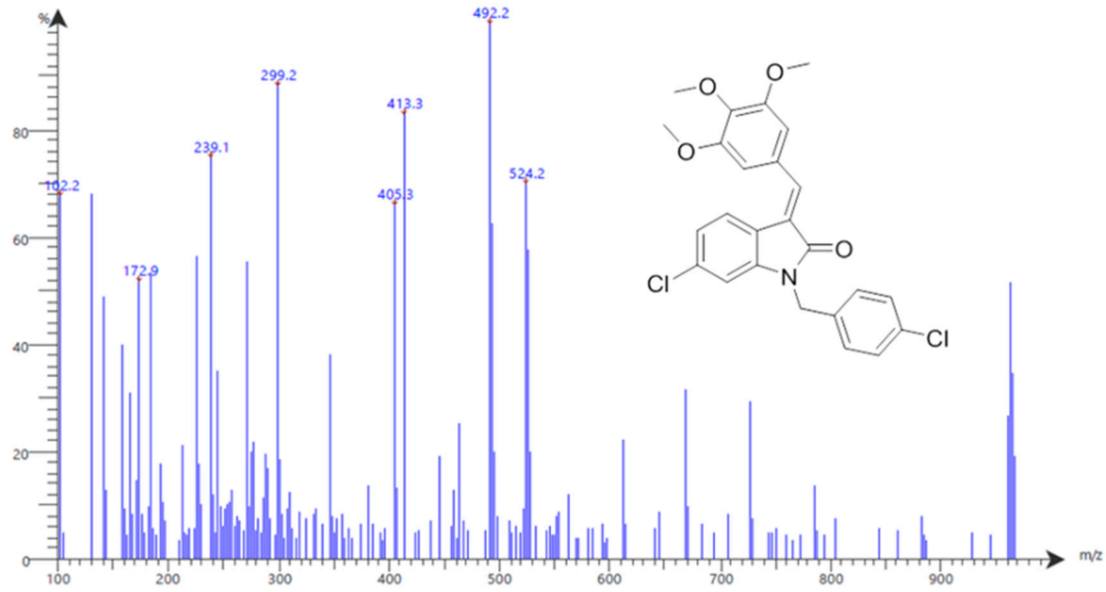

Figure S24. LC/MS spectrum of compound **6e.**

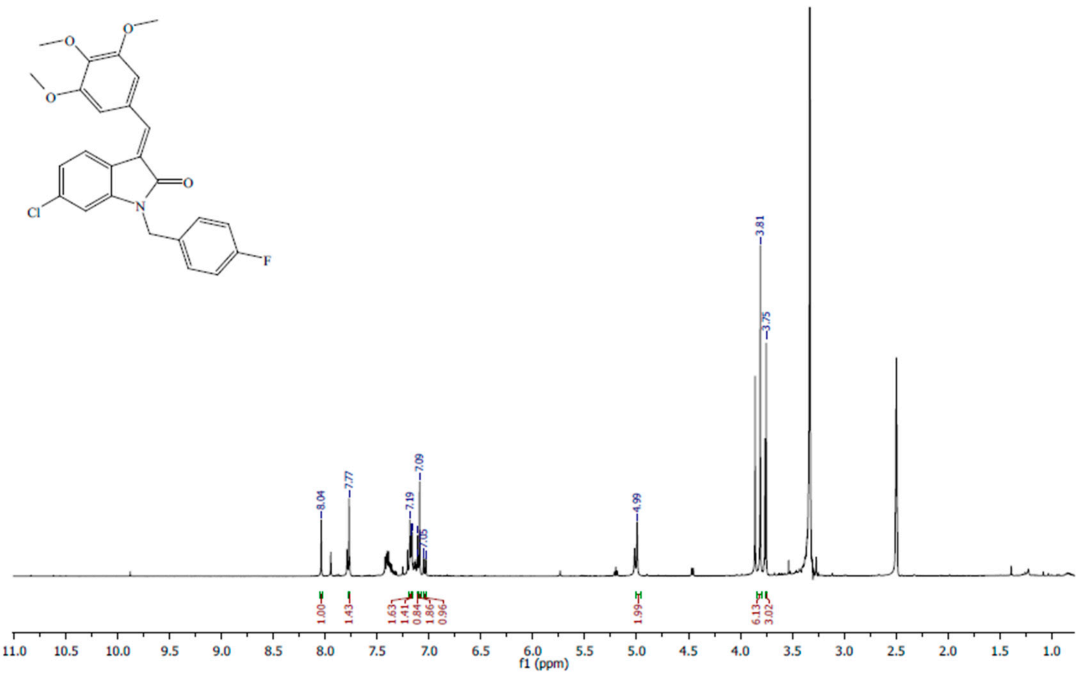

Figure S25. ^{1}H-NMR spectrum of compound **6f** in DMSO-*d6* at 400MHz.

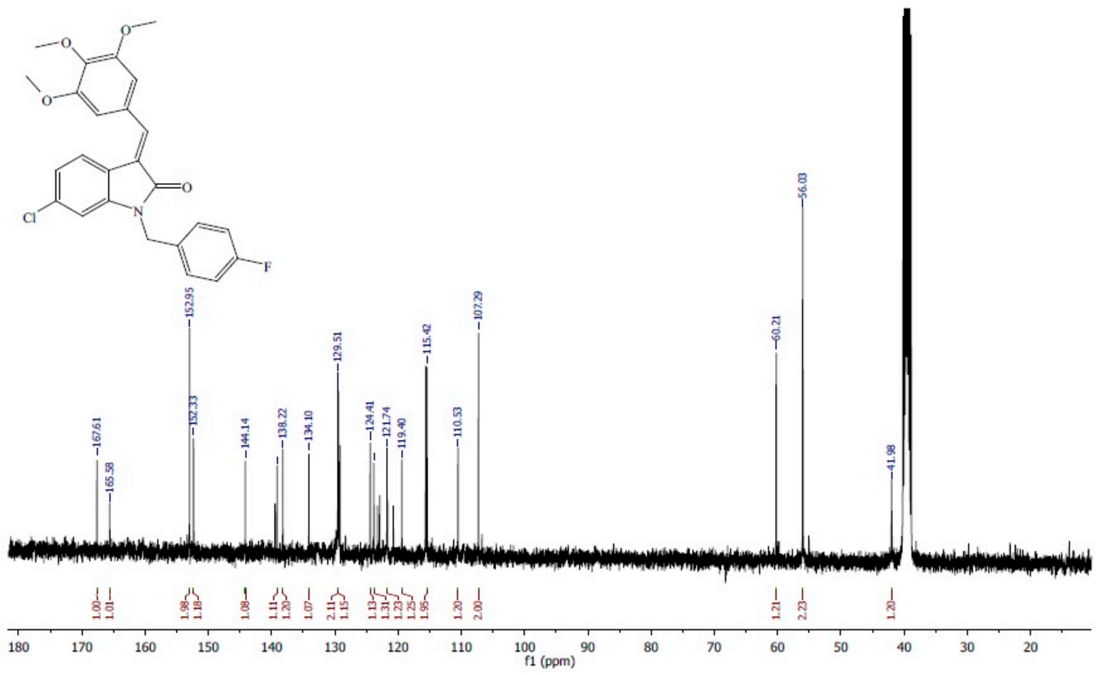

Figure S26. ^{13}C-NMR spectrum of compound **6f** in DMSO-*d6* at 100MHz.

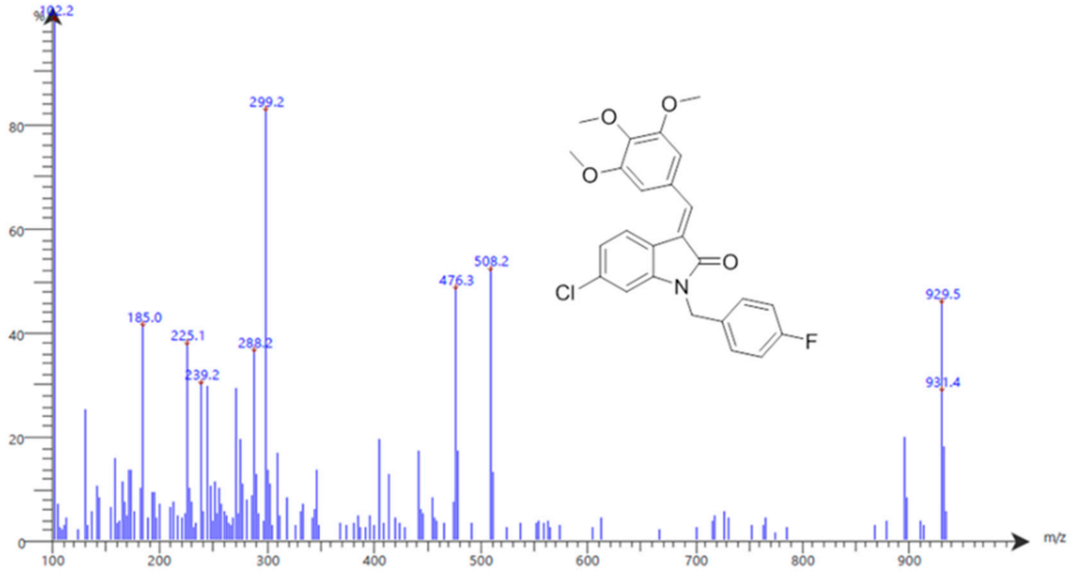

Figure S27. LC/MS spectrum of compound **6f.**

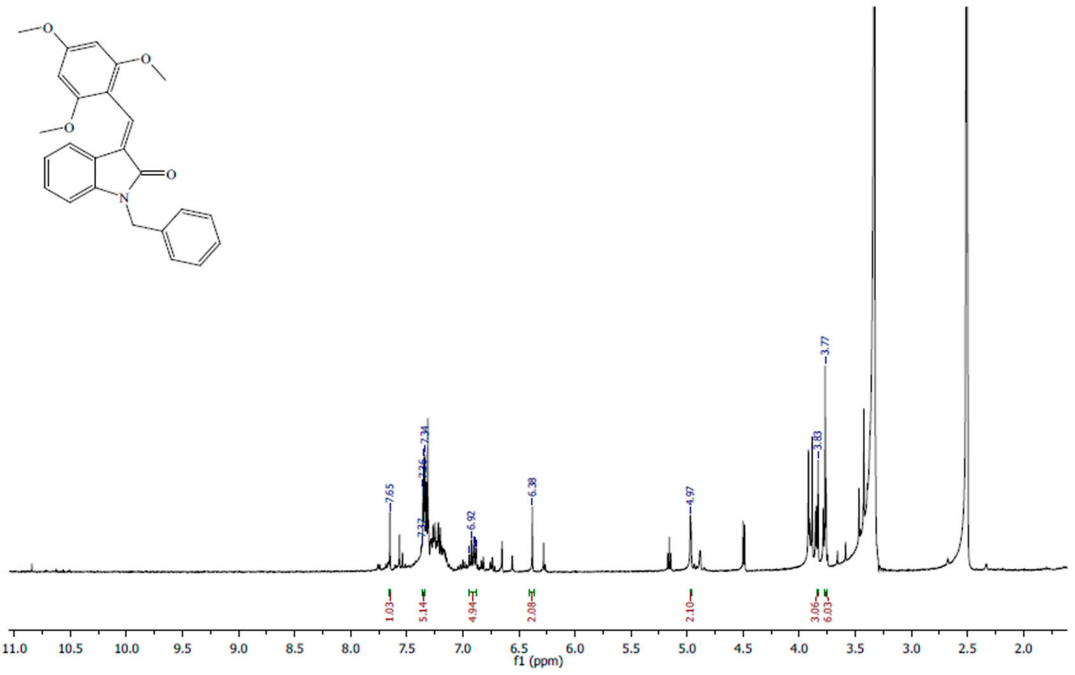

Figure S28. ^{1}H-NMR spectrum of compound **7a** in DMSO-*d6* at 400MHz.

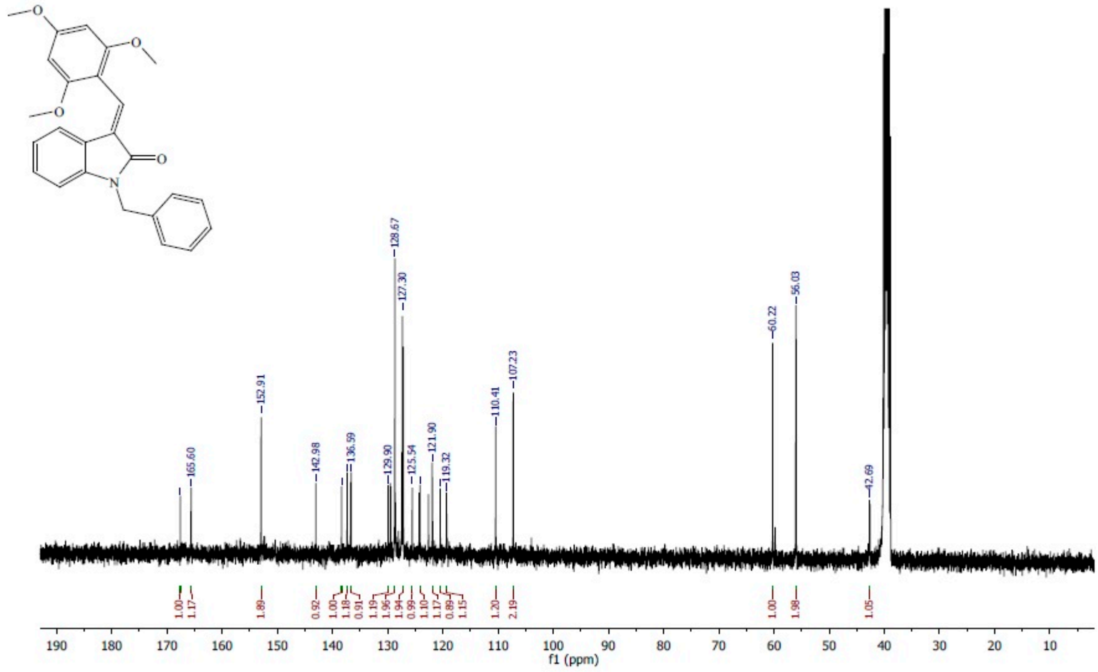

Figure S29. ^{13}C-NMR spectrum of compound **7a** in DMSO-*d6* at 100MHz.

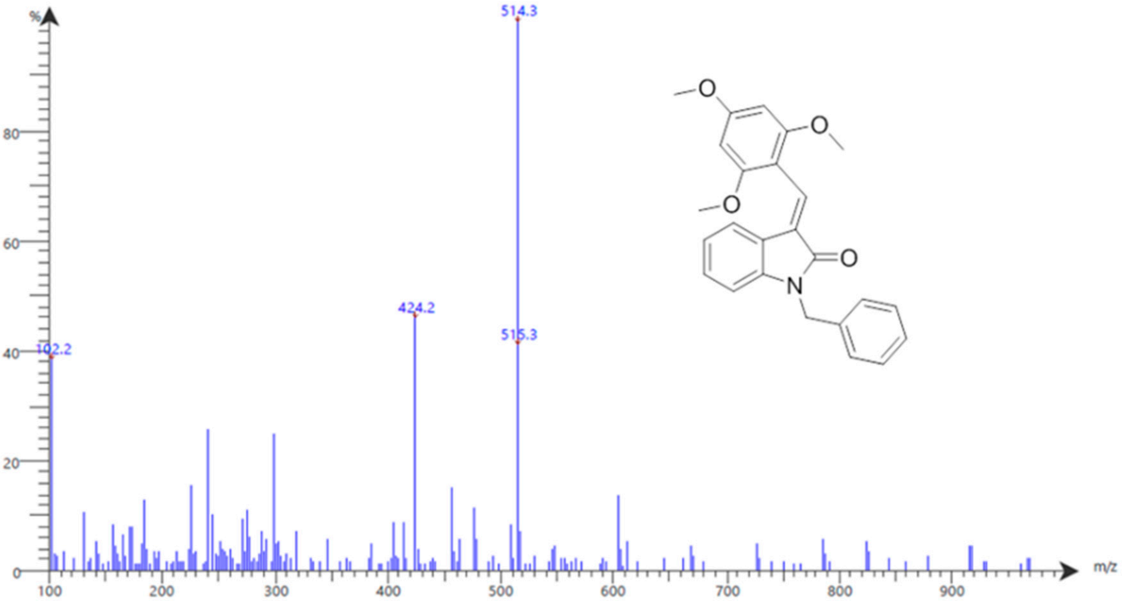

Figure S30. LC/MS spectrum of compound **7a.**

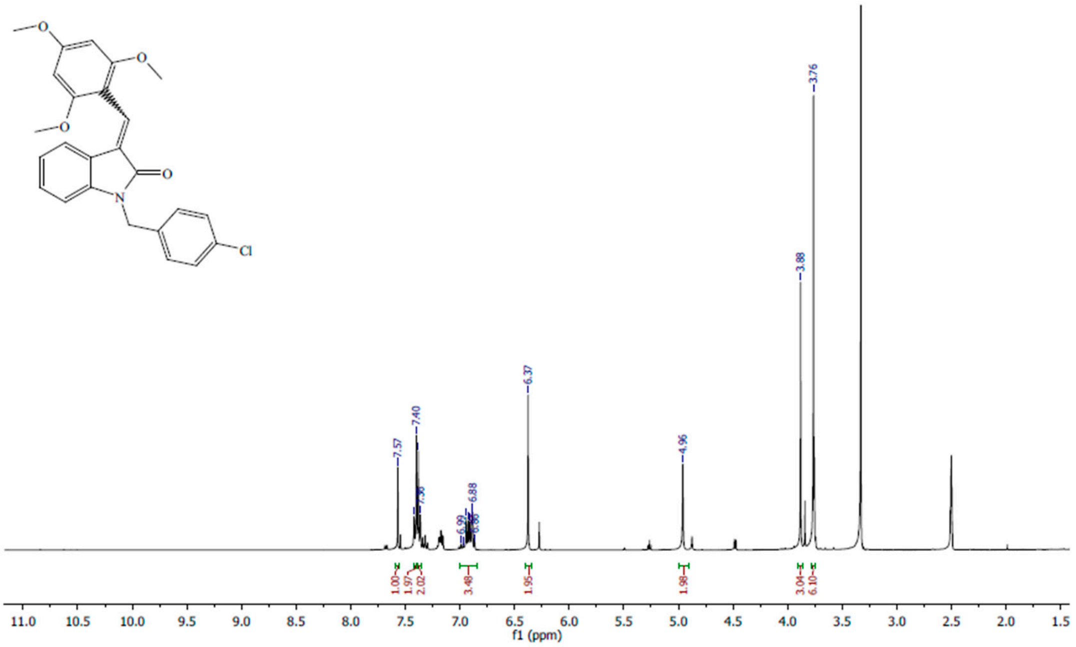

Figure S31. ^{1}H-NMR spectrum of compound **7b** in DMSO-*d6* at 400MHz.

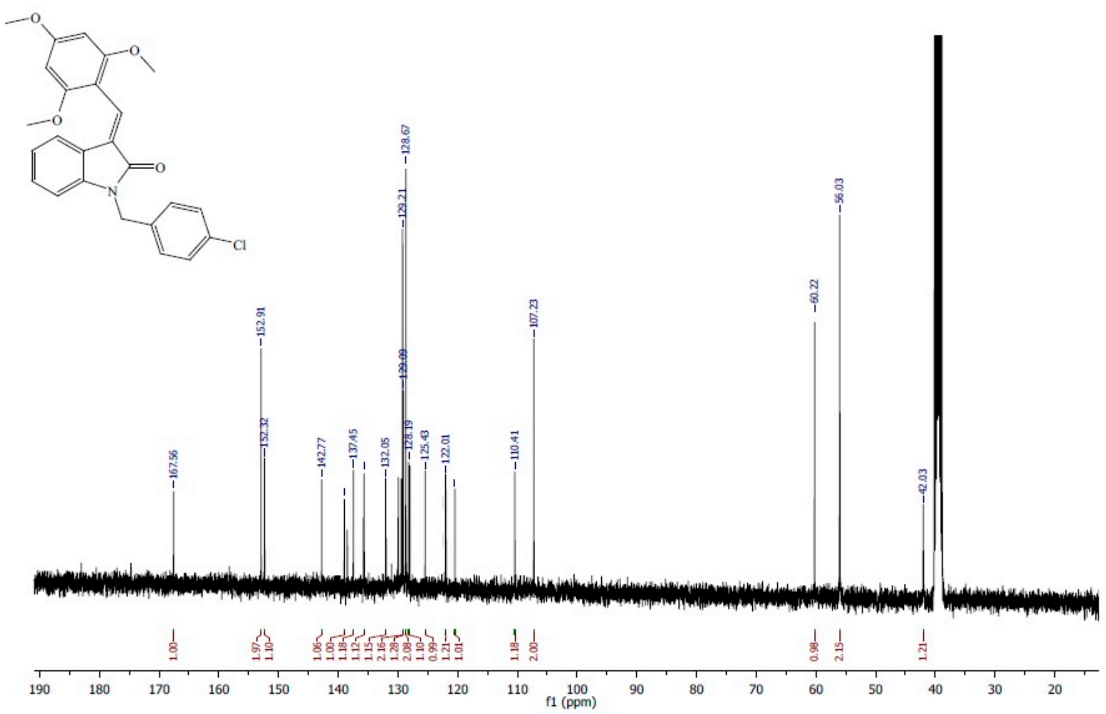

Figure S32. ^{13}C-NMR spectrum of compound **7b** in DMSO-*d6* at 100MHz.

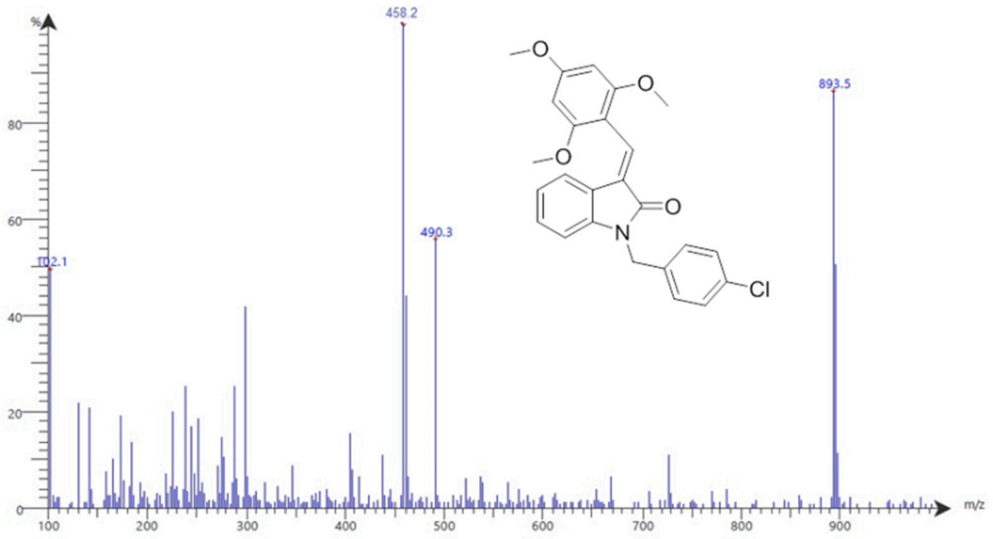

Figure S33. LC/MS spectrum of compound **7b.**

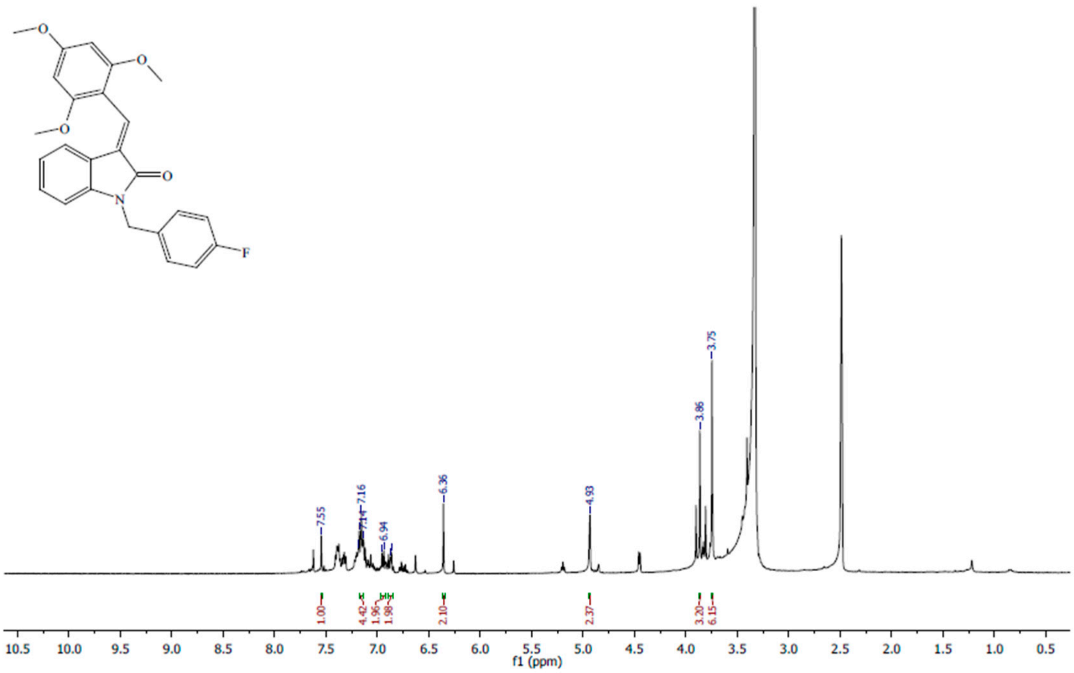

Figure S34. ^{1}H-NMR spectrum of compound **7c** in DMSO-*d6* at 400MHz.

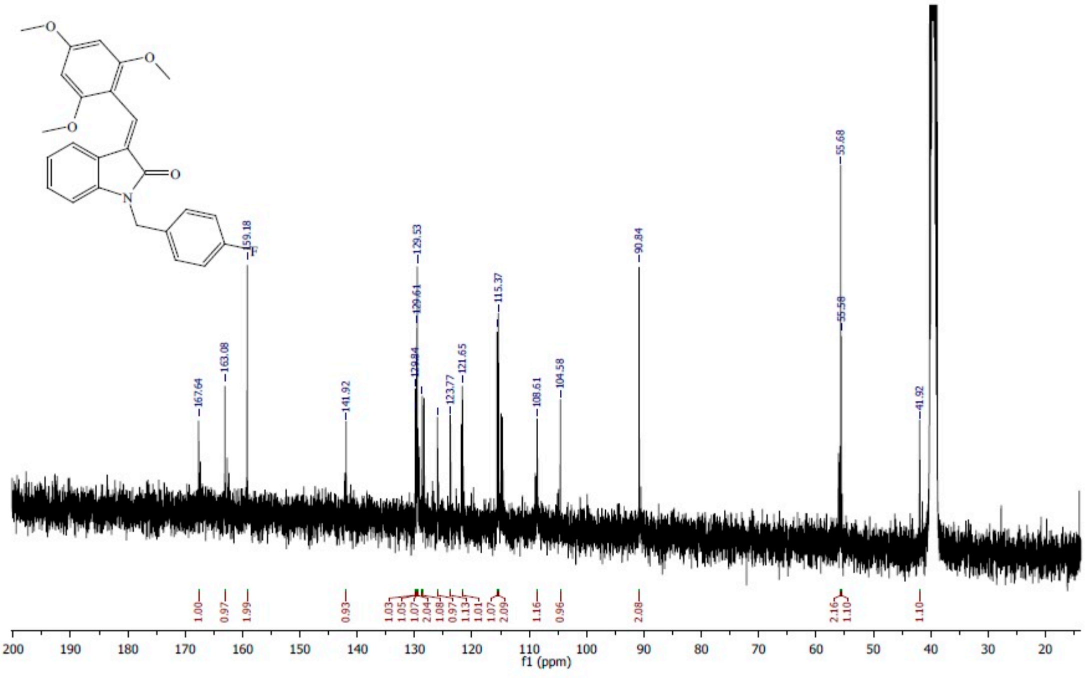

Figure S35. ^{13}C-NMR spectrum of compound **7c** in DMSO-*d6* at 100MHz.

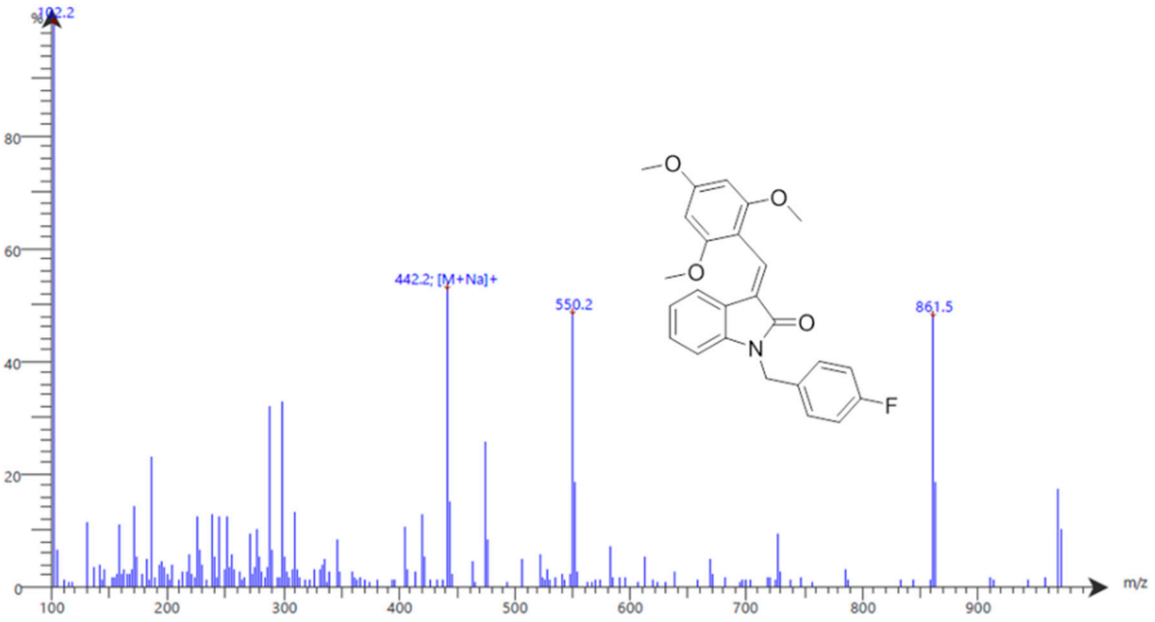

Figure S36. LC/MS spectrum of compound **7c.**

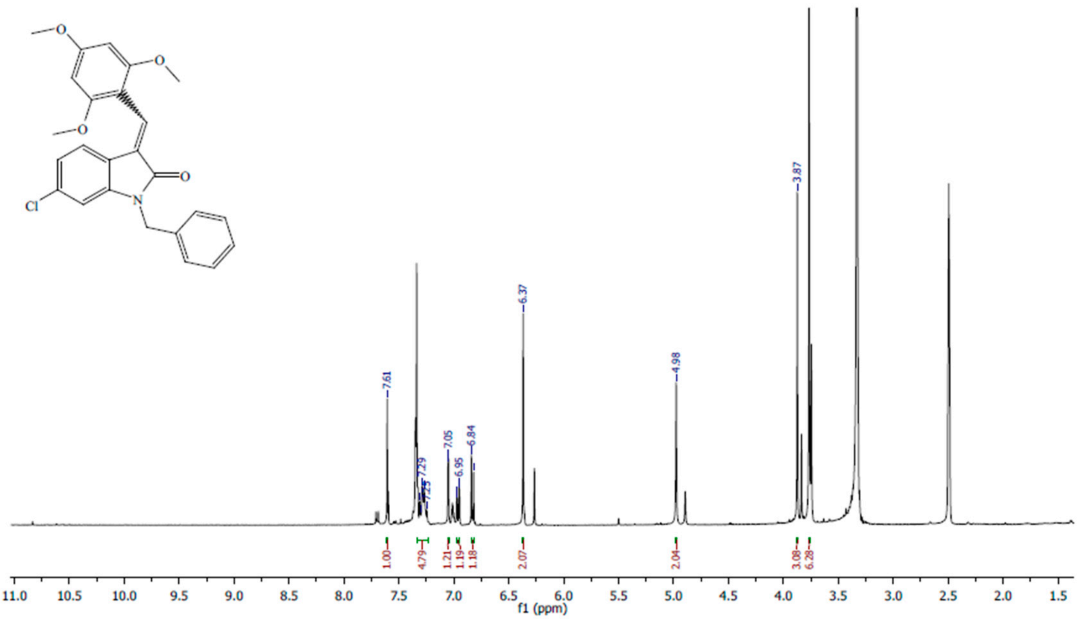

Figure S37. ¹H-NMR spectrum of compound **7d** in DMSO-*d6* at 400MHz.

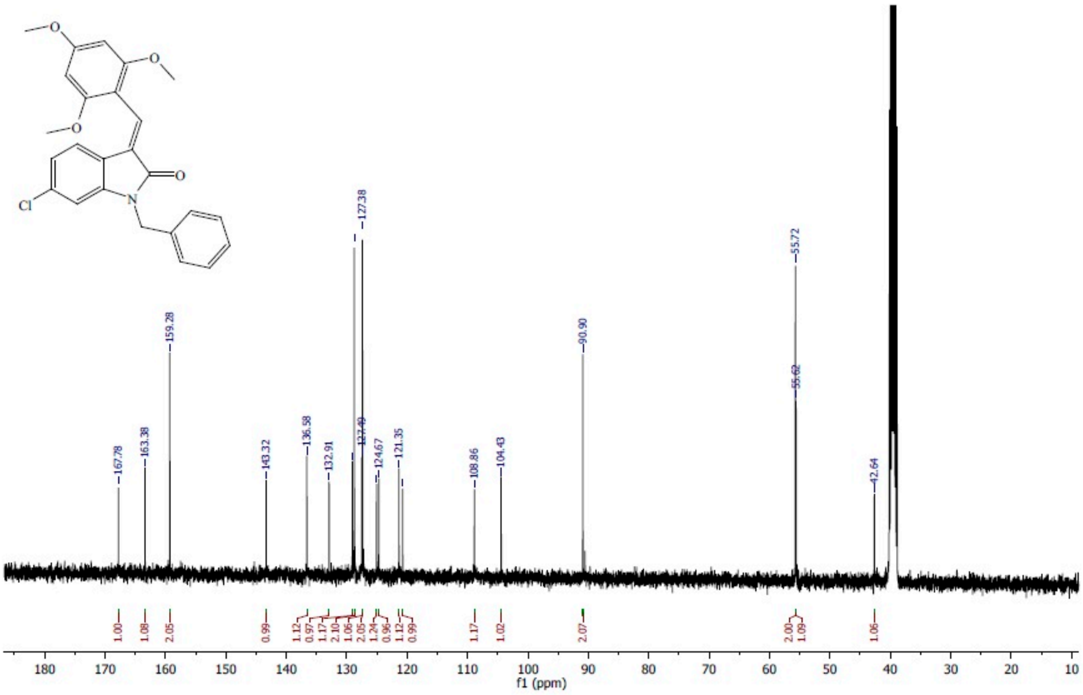

Figure S38. ^{13}C-NMR spectrum of compound **7d** in DMSO-*d6* at 100MHz.

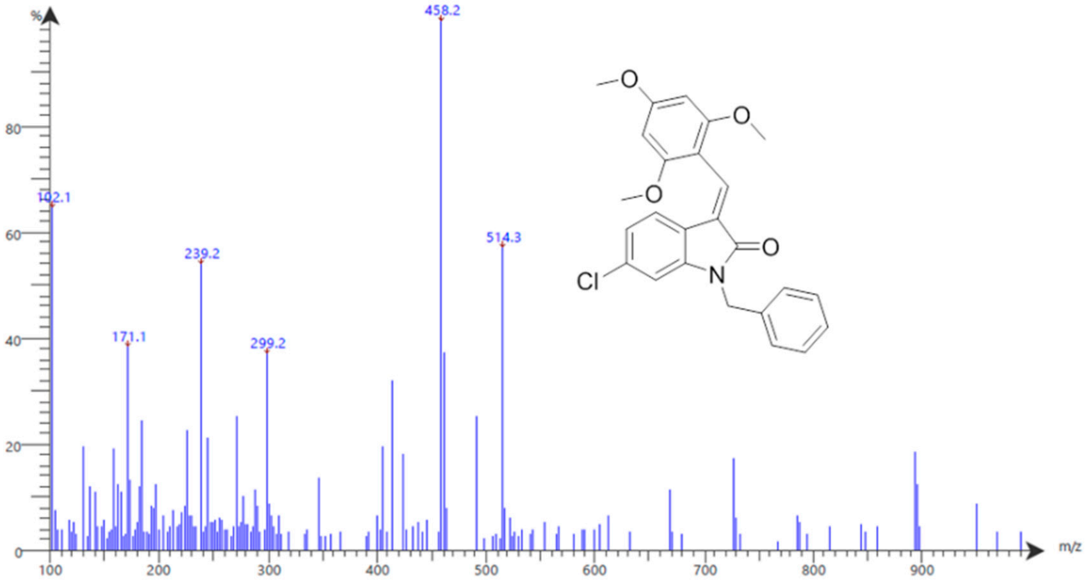

Figure S39. LC/MS spectrum of compound **7d.**

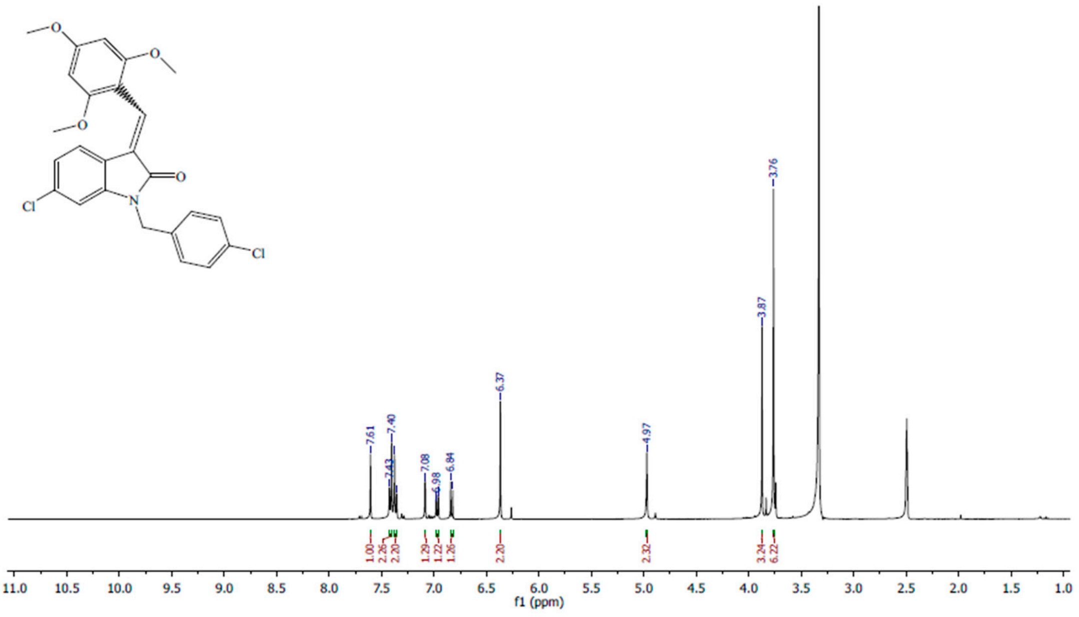

Figure S40. ^{1}H-NMR spectrum of compound **7e** in DMSO-*d6* at 400MHz.

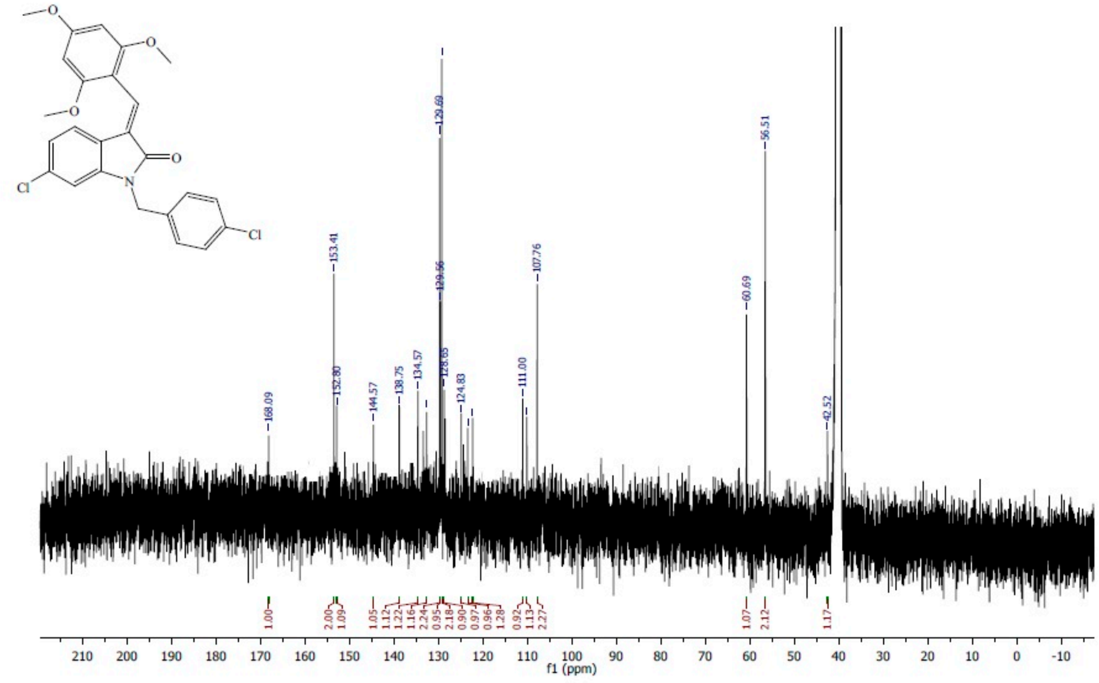

Figure S41. ^{13}C-NMR spectrum of compound **7e** in DMSO-*d6* at 100MHz.

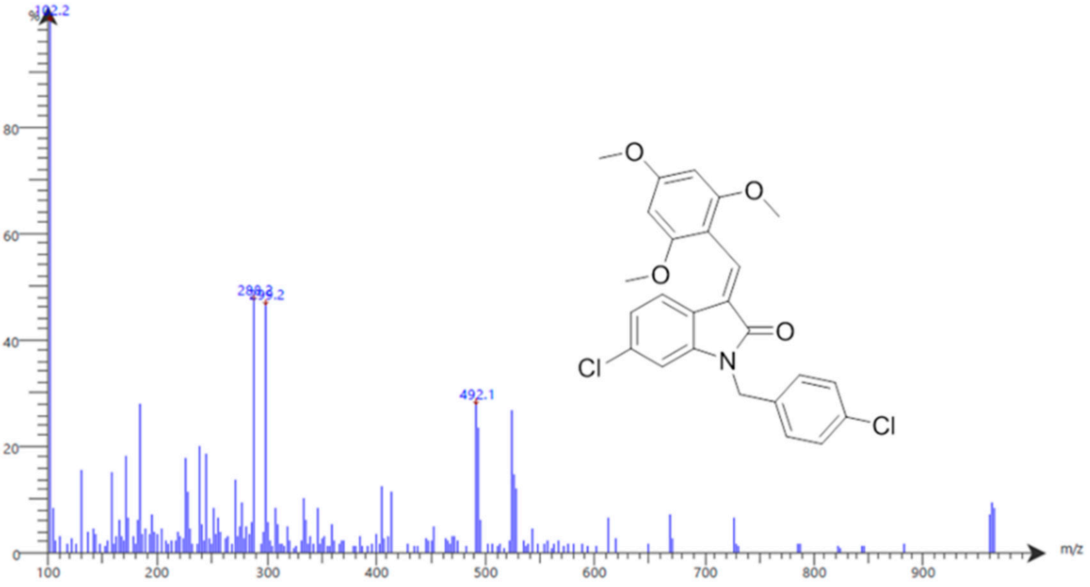

Figure S42. LC/MS spectrum of compound **7e.**

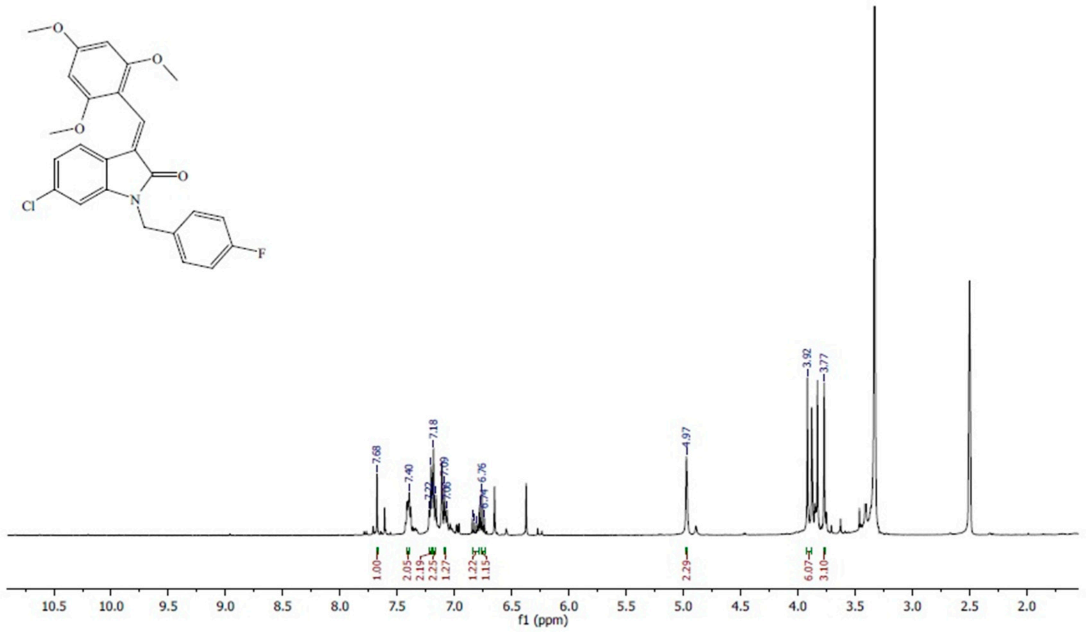

Figure S43. ¹H-NMR spectrum of compound **7f** in DMSO-*d6* at 400MHz.

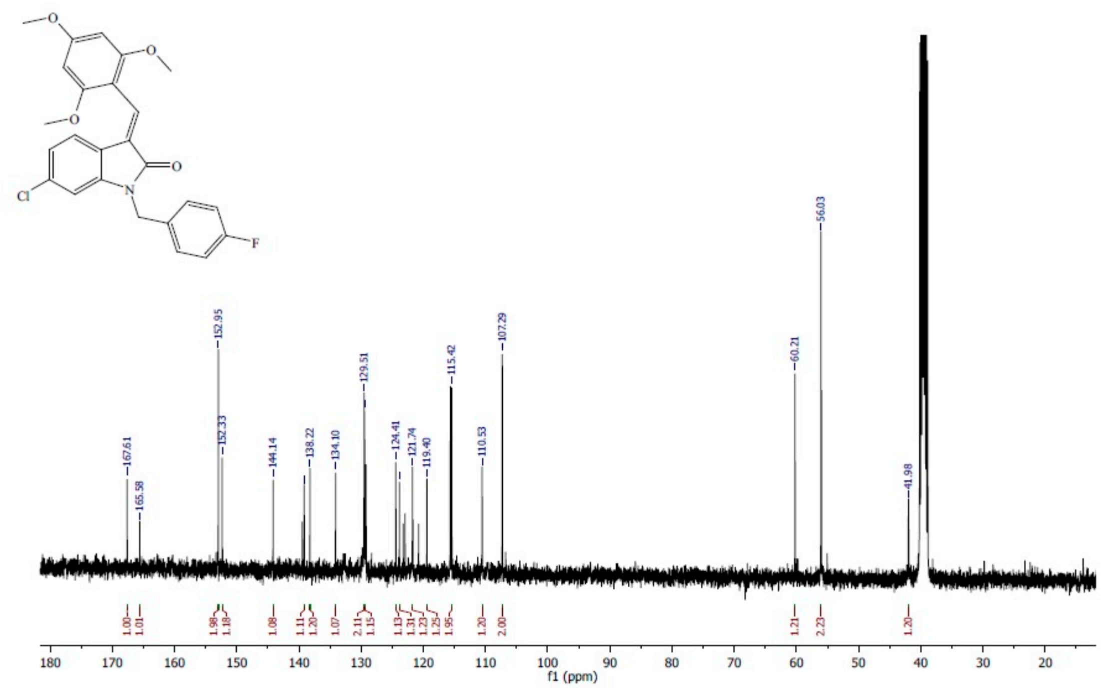

Figure S44. ^{13}C-NMR spectrum of compound **7f** in DMSO-*d6* at 100MHz.

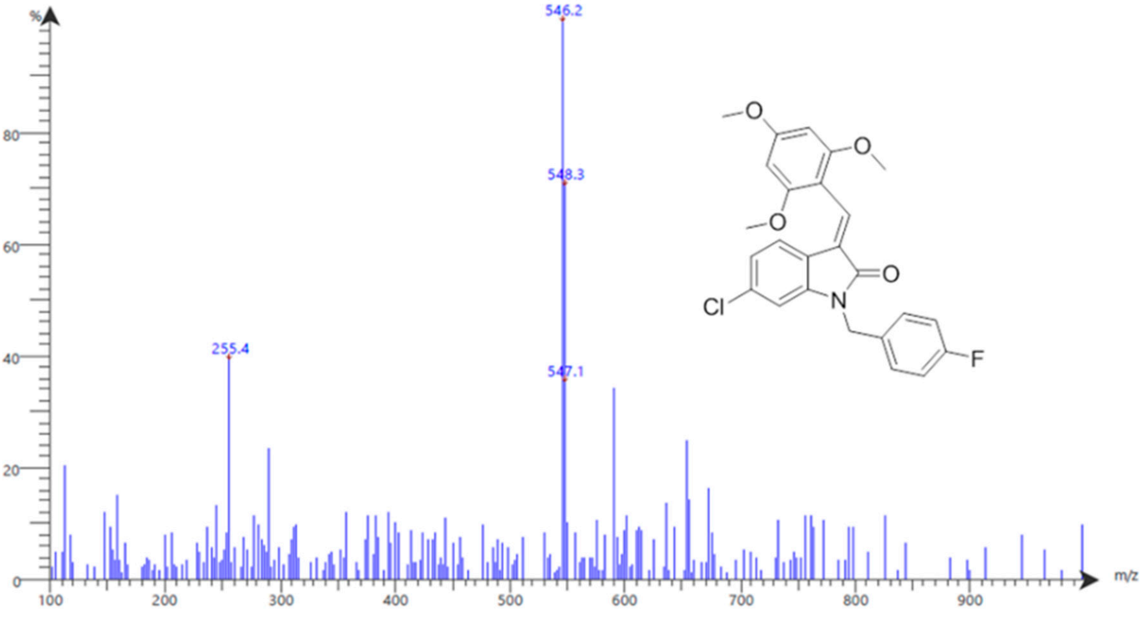

Figure S45. LC/MS spectrum of compound **7f.**

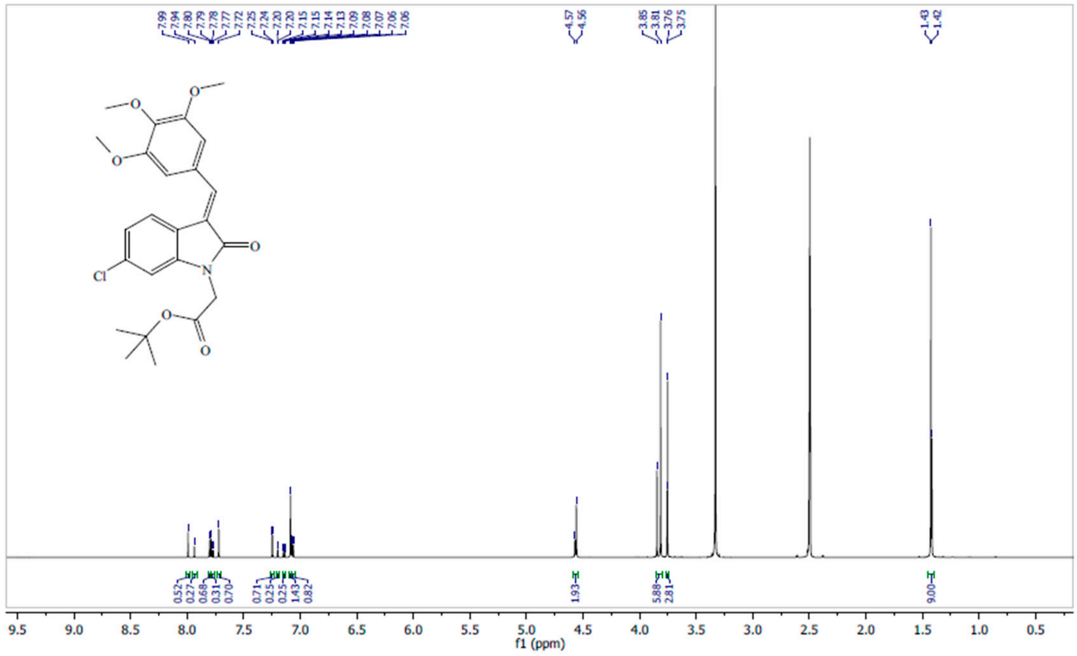

Figure S46. ^{1}H-NMR spectrum of compound **9a** in DMSO-*d6* at 400MHz.

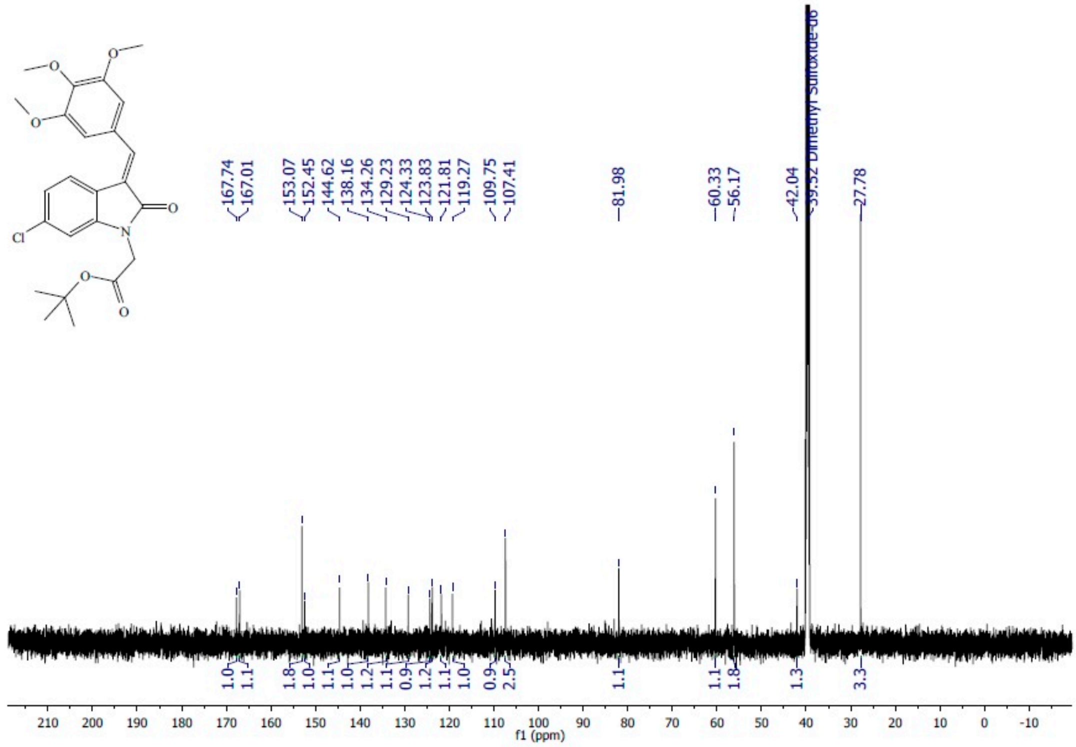

Figure S47. ^{13}C-NMR spectrum of compound **9a** in DMSO-*d6* at 100MHz.

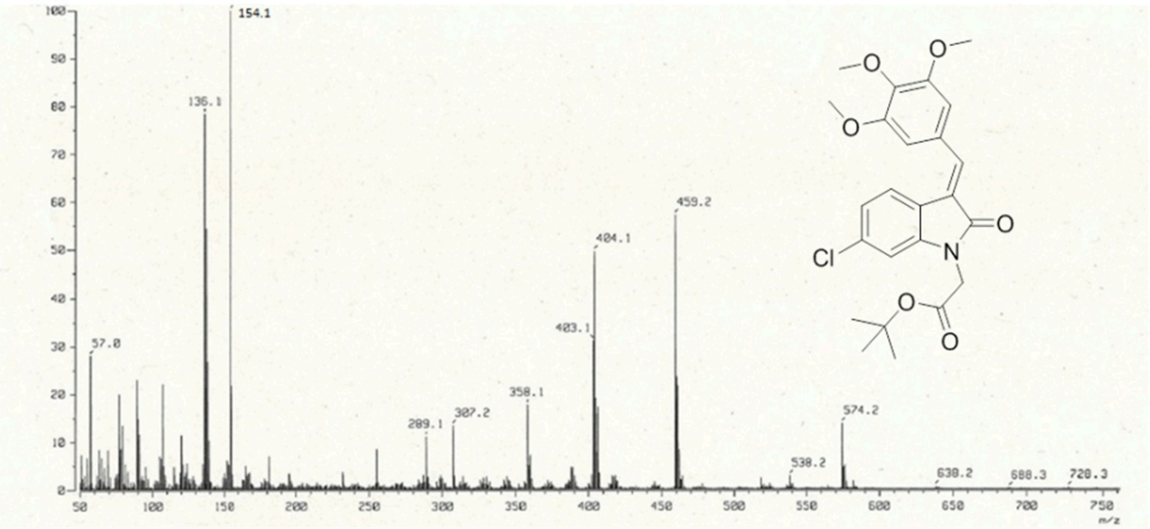

Figure S48. LC/MS spectrum of compound **9a.**

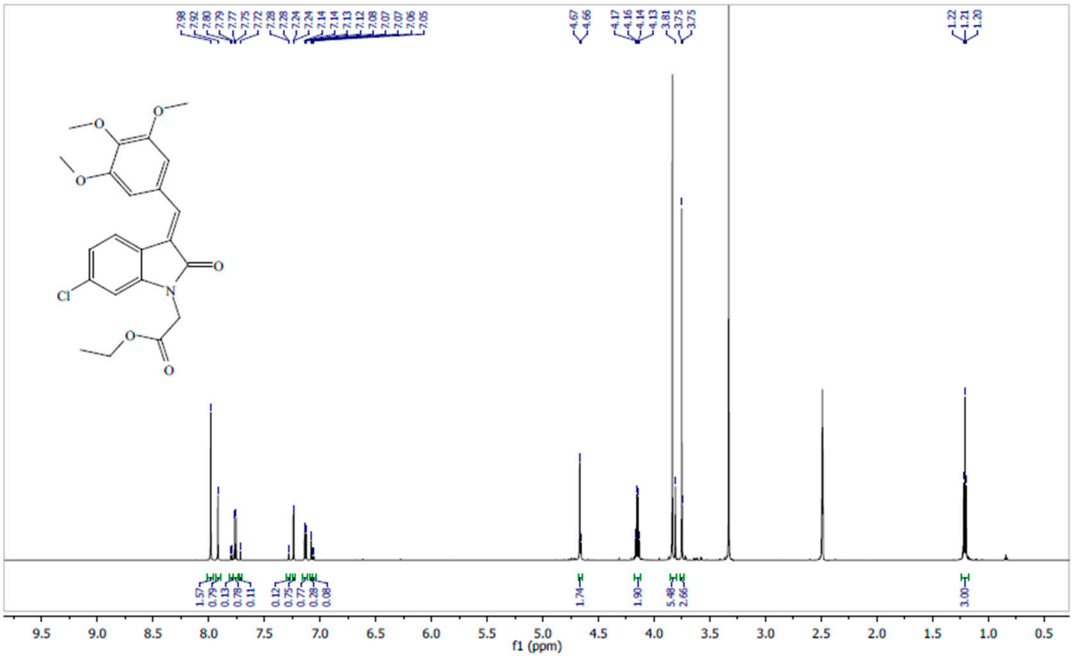

Figure S49. ^{1}H-NMR spectrum of compound **9b** in DMSO-*d6* at 400MHz.

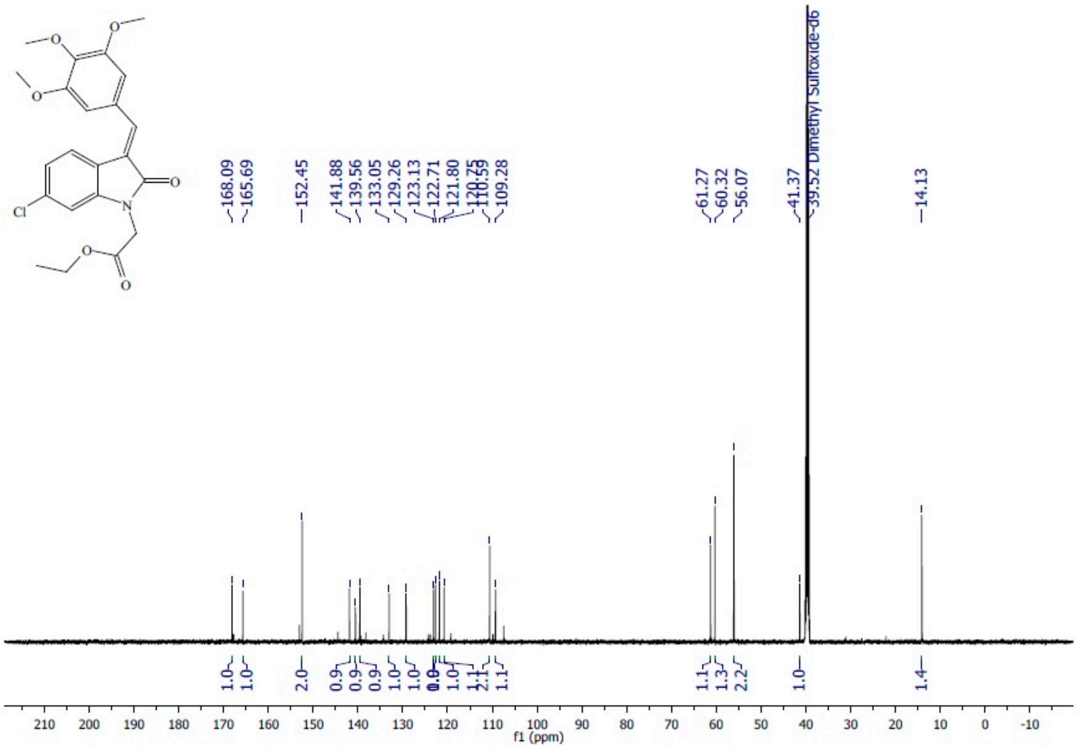

Figure S50. ^{13}C-NMR spectrum of compound **9b** in DMSO-*d6* at 100MHz.

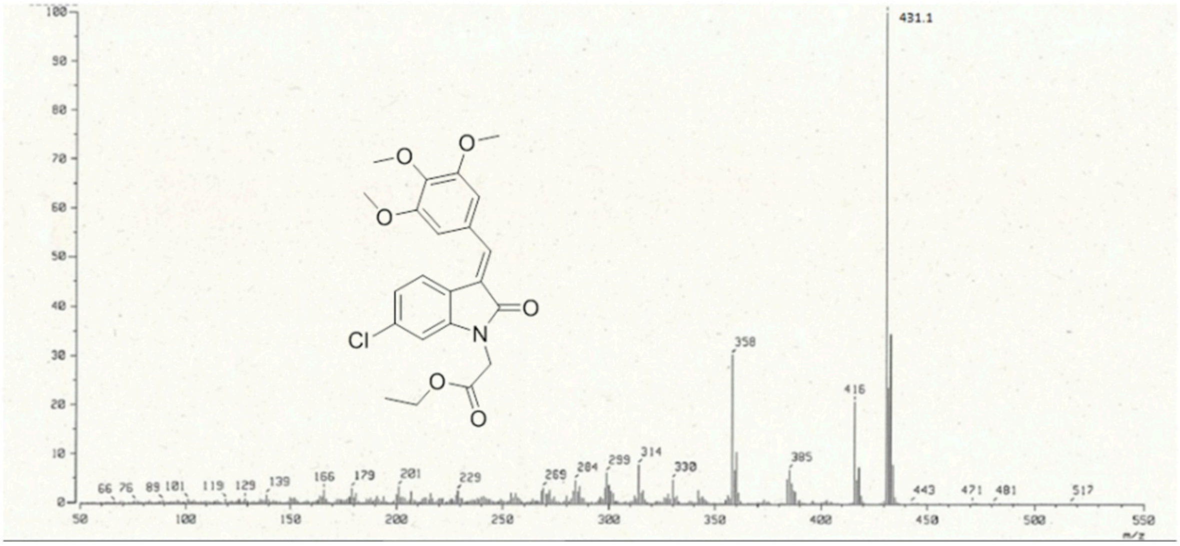

Figure S51. LC/MS spectrum of compound **9b.**

Detailed Results

Tub

code	IC50	conc.	log	%inh	T2	T1	ΔT	RFU2	RFU1	ΔRFU	slope	K.Activity	EC
4b		100	2	91.7	30	0	30	1347	0	1347	539.47	9.98758	120
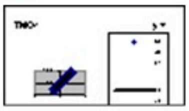													
		10	1	69.4	30	0	30	4955	0	4955	539.47	36.73976	120
		1	0	42.5	30	0	30	9303	0	9303	539.47	68.97881	120
		0.1	-1	22.7	30	0	30	12517	0	12517	539.47	92.80961	120
EC				0	30	0	30	16184	0	16184	539.47	120	120

code	IC50	conc.	log	%inh	T2	T1	ΔT	RFU2	RFU1	ΔRFU	slope	K.Activity	EC
CA4		100	2	92.3	30	0	30	1161	0	1161	501.27	9.264468	120
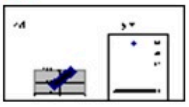													
		10	1	75.8	30	0	30	3638	0	3638	501.27	29.03026	120
		1	0	54.5	30	0	30	6842	0	6842	501.27	54.59732	120
		0.1	-1	39.8	30	0	30	9057	0	9057	501.27	72.27243	120
EC				0	30	0	30	15038	0	15038	501.27	120	120

**Assay**	: Tubulin polymerization assay
**Samples**	: 02 compounds
**Date**	: 09-03-2021
**Reader**	: Tecan –spark READER wl Ex. 360 nm and Em. 450 nm.
**Solvent**	: DMSO

Lab Report

ser	Compound			Results<br>Tubulin polymerization inh.	SD ±
	ID	M.W g/mol	conc uM	IC50 uM	
1	4b	345.0		1.66	0.08
2	CA4	334.36		0.42	0.02

Figure S52. Tubulin polymerization assay for compound 4b.

*Detailed Results:

Tub

code	IC50	conc	log	%inh	T2	T1	ΔT	RFU2	RFU1	ΔRFU	slope	K.Activity	EC
NH10		100	2	78	30	0	30	0.085	0	0.085	0.013	26.15385	120
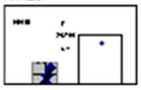		10	1	43	30	0	30	0.224	0	0.224	0.013	68.92308	120
		1	0	18	30	0	30	0.319	0	0.319	0.013	98.15385	120
		0.1	-1	4	30	0	30	0.375	0	0.375	0.013	115.3846	120
EC				0	30	0	30	0.404	0	0.404	0.013	120	120

code	IC50	conc.uM	log conc	%inh	T2	T1	ΔT	RFU2	RFU1	ΔRFU	slope	K.Activity	EC
NH24		100	2	82	30	0	30	0.071	0	0.071	0.013	21.84615	120
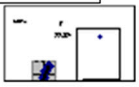		10	1	59	30	0	30	0.159	0	0.159	0.013	48.92308	120
		1	0	31	30	0	30	0.269	0	0.269	0.013	82.76923	120
		0.1	-1	15	30	0	30	0.331	0	0.331	0.013	101.8462	120
EC				0	30	0	30	0.404	0	0.404	0.013	120	120

NH10	y = 24.744x + 23.333
NH24	y = 22.821x + 35.385
CA-4	y = 20.795x + 41.974

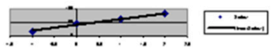

Researcher : Dr. Mohamed badr email : mohabadr180@gmail.com mob. 01067157143
Assay : Tubulin enzyme assay
Samples : 02 compounds
Ref. : ---
Date : 28-09-2021
Reader : Tecan-Spark reader
Kit used : Cloud-clone corp) SEB870Hu EIA Kit For Tubulin Beta (TUBb).
Solvent : DMSO
Assay samples : ---

Lab Report

ser	Compound			Tubulin polymerization Inhibition	SD ±
	ID	M.W g/mol	conc	IC50 uM	
1	4a	311.12		5.045	0.31
2	CA-4	316.34		1.384	0.09

Figure S53. Tubulin polymerization assay for compound 4a.

Detailed results

[redacted]

EGFR [redacted]

code	IC50	conc	log	%inh	T2	T1	ΔT	RFU2	RFU1	ΔRFU	slope	K.Activity
4e		10	1	78	30	0	30	22.06	0	22.06	3.33333	26.47203
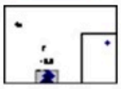		1	0	60	30	0	30	39.55	0	39.55	3.33333	47.46005
		0.1	-1	33	30	0	30	67.25	0	67.25	3.33333	80.70008
		0.01	-2	21	30	0	30	79.42	0	79.42	3.33333	95.3041
EC				0	30	0	30	100	0	100	3.33333	120

[redacted]

code	IC50	conc.ng/ml	log conc	%inh	T2	T1	ΔT	RFU2	RFU1	ΔRFU	slope	K.Activity
4b		10	1	88	30	0	30	11.51	0	11.51	3.33333	13.81201
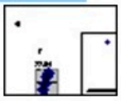		1	0	69	30	0	30	31.42	0	31.42	3.33333	37.70404
		0.1	-1	40	30	0	30	59.96	0	59.96	3.33333	71.95207
		0.01	-2	23	30	0	30	77.13	0	77.13	3.33333	92.55609
EC				0	30	0	30	100	0	100	3.33333	120

[redacted]

code	IC50	conc.ng/ml	log conc	%inh	T2	T1	ΔT	RFU2	RFU1	ΔRFU	slope	K.Activity
Gefitinib		10	1	93	30	0	30	7.39	0	7.39	3.33333	8.868009
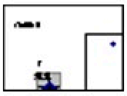		1	0	82	30	0	30	18.34	0	18.34	3.33333	22.00802
		0.1	-1	56	30	0	30	44.12	0	44.12	3.33333	52.94405
		0.01	-2	32	30	0	30	68.13	0	68.13	3.33333	81.75608
EC				0	30	0	30	100	0	100	3.33333	120

[redacted]

**4e**	y = 19.978x + 57.919
**4b**	y = 22.54x + 66.265
**Gefitinib**	y = 20.8x + 75.905

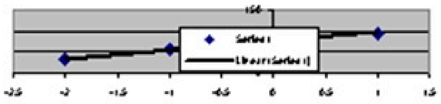

Researcher	: Dr.Mohamed Ibrahim email: Mia06@fayoum.edu.eg mob. 01025727434
Assay	: EGFR inh.assay
Samples	: 02 compounds
Cell lines	: ---
Ref.	: ---
Date	: 10-05-2021
Reader	: ROBONIK P2000 ELISA READER wl 450 nm
Kit used	: ---.
Solvent	: DMSO

Lab Report

ser	Compound			EGFR	SD ±
	code	MW	conc. ug/ml	IC50 ug/ml	
1	4e			0.401	0.008
2	4b			0.19	0.004
3	Gefitinib			0.057	0.001

Figure S54. Epidermal growth factor receptor (EGFR) inhibition assay for compound 4b.

Detailed results

[redacted]

EGFR

[redacted]

code	IC50	conc	log	%inh	T2	T1	ΔT	RFU2	RFU1	ΔRFU	slope	K.Activity	EC
4a		10	1	89	30	0	30	11.42	0	11.42	3.33333	13.70401	120
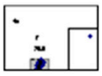		1	0	72	30	0	30	27.55	0	27.55	3.33333	33.06003	120
		0.1	-1	50	30	0	30	49.65	0	49.65	3.33333	59.58006	120
		0.01	-2	27	30	0	30	72.51	0	72.51	3.33333	87.01209	120
EC				0	30	0	30	100	0	100	3.33333	120	120

[redacted]

code	IC50	conc.ng/ml	log conc	%inh	T2	T1	ΔT	RFU2	RFU1	ΔRFU	slope	K.Activity	EC
CMJ		10	1	93	30	0	30	6.56	0	6.56	3.33333	7.872008	120
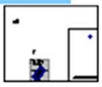		1	0	83	30	0	30	17.02	0	17.02	3.33333	20.42402	120
		0.1	-1	59	30	0	30	41.05	0	41.05	3.33333	49.26005	120
		0.01	-2	36	30	0	30	63.95	0	63.95	3.33333	76.74008	120
EC				0	30	0	30	100	0	100	3.33333	120	120

[redacted]

s4	y = 20.537x + 69.986
s5	y = 19.62x + 77.665

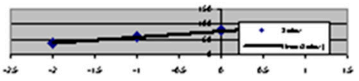

Researcher	: Dr. Mohamed badr email : mohabadr180@gmail.com mob. 01067157143
Assay	: EGFR inh.assay
Samples	: 02 compounds
Cell lines	: ---
Ref.	: ---
Date	: 29-09-2021
Reader	: Tecan Spark Reader
Kit used	: ---.
Solvent	: DMSO

Lab Report

ser	Compound			EGFR	SD ±
	code	MW	conc. ug/ml	IC50 uM	
1	4a	311.12		0.106	0.0022
2	CJM 126	226.30		0.039	0.0008

Figure S55. Epidermal growth factor receptor (EGFR) inhibition assay for compound 4a.

Detailed results

[redacted]

CK1													
code	IC50	conc	log	%inh	T2	T1	ΔT	RFU2	RFU1	ΔRFU	slope	K.Activity	EC
4e		100	2	65.47	30	0	30	0.634	0	0.634	0.0612	41.4379	120
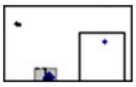		10	1	48.15	30	0	30	0.952	0	0.952	0.0612	62.2222	120
		1	0	30.23	30	0	30	1.281	0	1.281	0.0612	83.7255	120
		0.1	-1	12.64	30	0	30	1.604	0	1.604	0.0612	104.837	120
EC				0	30	0	30	1.836	0	1.836	0.0612	120	120

code	IC50	conc.ng/ml	log conc	%inh	T2	T1	ΔT	RFU2	RFU1	ΔRFU	slope	K.Activity	EC
4b		100	2	88.62	30	0	30	0.209	0	0.209	0.0612	13.6601	120
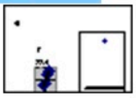		10	1	69.99	30	0	30	0.551	0	0.551	0.0612	36.0131	120
		1	0	35.62	30	0	30	1.182	0	1.182	0.0612	77.2549	120
		0.1	-1	25.16	30	0	30	1.374	0	1.374	0.0612	89.8039	120
EC				0	30	0	30	1.836	0	1.836	0.0612	120	120

code	IC50	conc.ng/ml	log conc	%inh	T2	T1	ΔT	RFU2	RFU1	ΔRFU	slope	K.Activity	EC
IC261		100	2	85.59	30	0	30	0.287	0	0.287	0.0664	17.2892	120
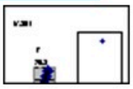		10	1	61.7	30	0	30	0.763	0	0.763	0.0664	45.9639	120
		1	0	36.8	30	0	30	1.259	0	1.259	0.0664	75.8434	120
		0.1	-1	25.9	30	0	30	1.476	0	1.476	0.0664	88.9157	120
EC				0	30	0	30	1.993	0	1.993	0.0664	120	120

**4e**	$y = 17.642x + 30.3$
**4b**	$y = 22.473x + 43.611$
**IC261**	$y = 20.397x + 42.299$

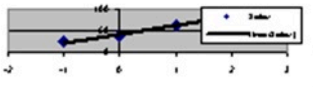

Researcher : Dr.Mohamed Ibrahim email: Mia06@fayoum.edu.egmob. 01025727434
Assay : CK1 assay
Samples : 02compounds
Cell lines : ---
Ref. : ---
Date : 20-05-2021
Reader : ROBONIK P2000 ELISA READER wl 450 nm
Kit used : ---.
Solvent : DMSO

Lab Report

ser	Compound			Casein kinase I CK1	SD ±
	code	MW	conc. ug/ml	IC50 ug/ml	
1	4e			13.08	0.66
2	4b			1.92	0.09
3	IC261			2.385	0.12

Figure S56. Casein kinase I (CK1) inhibition assay

detailed results

STANDARDS	Pg/ml
St.1	1000
St.2	500
St.3	250
St.4	100
St.5	50

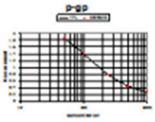

Plate map

	1	2	3	4	5	6	7	8	9	10	11	12
A	st 1	4b	--	--	--	--	--	--	--	--	--	--
B	st 2	4b	--	--	--	--	--	--	--	--	--	--
C	st 3	4b	--	--	--	--	--	--	--	--	--	--
D	st 4	Ketoconazole	--	--	--	--	--	--	--	--	--	--
E	st 5	Ketoconazole	--	--	--	--	--	--	--	--	--	--
F	B	Ketoconazole	--	--	--	--	--	--	--	--	--	--
G	B	control	--	--	--	--	--	--	--	--	--	--
H	B	control	--	--	--	--	--	--	--	--	--	--

Samples OD results

	1	2	3	4	5	6	7	8	9	10	11	12
A	0.291	0.782	0	0	0	0	0	0	0	0	0	0
B	0.442	0.808	0	0	0	0	0	0	0	0	0	0
C	0.788	0.796	0	0	0	0	0	0	0	0	0	0
D	1.394	0.949	0	0	0	0	0	0	0	0	0	0
E	1.846	0.961	0	0	0	0	0	0	0	0	0	0
F	0.002	0.933	0	0	0	0	0	0	0	0	0	0
G	0.007	0.549	0	0	0	0	0	0	0	0	0	0
H	0.004	0.562	0	0	0	0	0	0	0	0	0	0

Calibrator	Wells	Conc.	Raw (Corrected)	Backfit	Recovery %
Standard1	A1	1000	0.287	962.5	96.25
Standard2	B1	500	0.438	522.3	104.5
Standard3	C1	250	0.784	245	98.01
Standard4	D1	100	1.39	100.7	100.7
Standard5	E1	50	1.84	49.84	99.68

Sample	Wells	Raw	Background Corrected	Conc.	Conc. (Average)	%CV	SD	SEM
Control	G2	0.549	0.551	393	387.1	2.14	8.3	5.87
	H2	0.562		381.2				
4b	A2	0.782	0.791	247.5	242	2.21	5.34	3.08
	B2	0.808		236.9				
	C2	0.796		241.7				
Ketoconazole	D2	0.949	0.943	189.4	189.8	2.13	4.05	2.34
	E2	0.961		186				
	F2	0.933		194.1				
Blank	F1	0.002	0	< Curve	-	-	-	-
	G1	0.007		< Curve				
	H1	0.004		< Curve				

Figure S57. P-gp substrate assay.

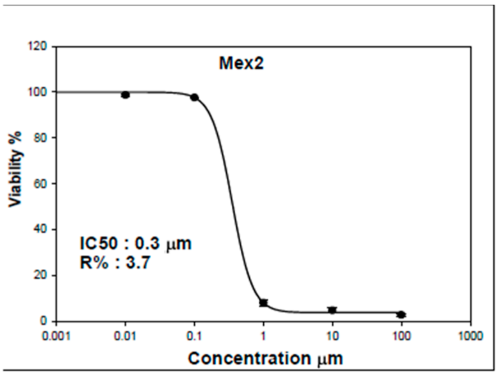
Mex2
Viability %
Concentration µm
IC50 : 0.3 µm
R% : 3.7
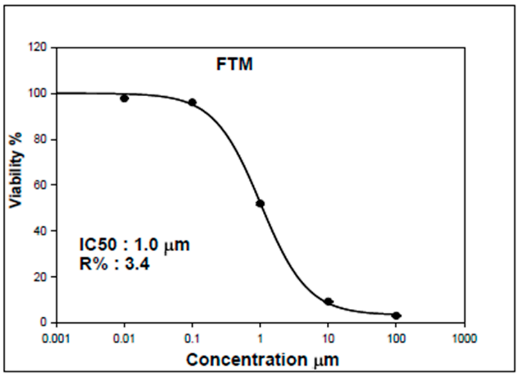
FTM
Viability %
Concentration µm
IC50 : 1.0 µm
R% : 3.4
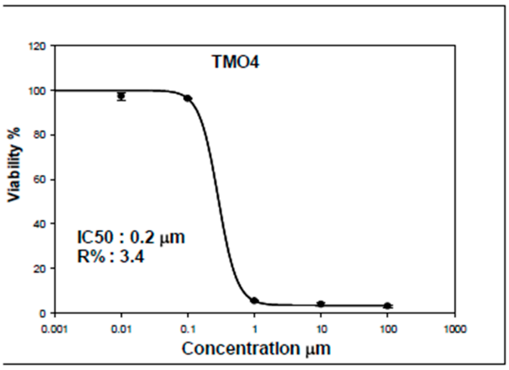
TMO4
Viability %
Concentration µm
IC50 : 0.2 µm
R% : 3.4
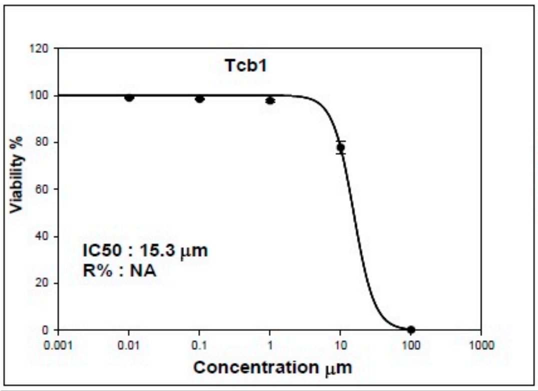
Tcb1
Viability %
Concentration µm
IC50 : 15.3 µm
R% : NA
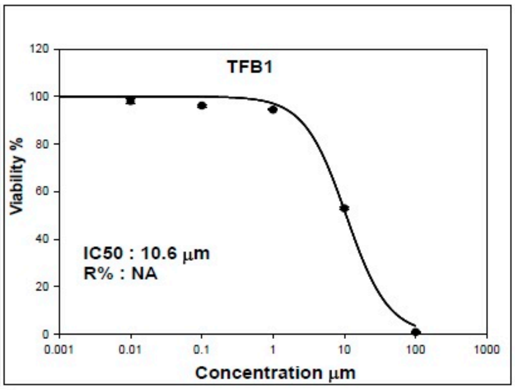
TFB1
Viability %
Concentration µm
IC50 : 10.6 µm
R% : NA
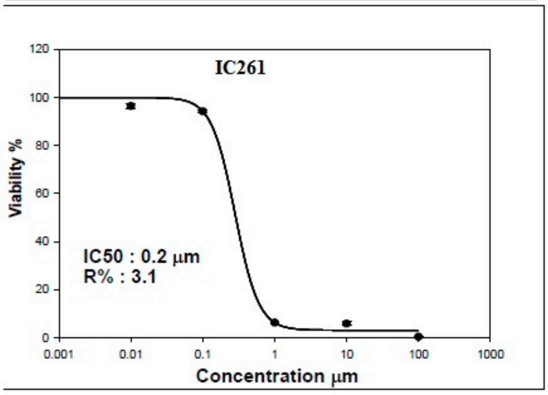
IC261
Viability %
Concentration µm
IC50 : 0.2 µm
R% : 3.1

Figure S58. IC_{50} of compounds **4a (IC261), 4b, 4d, 4e, 6e** and **6f against COLO-205 using SRB assay.**

|
Researcher	: Dr. Mohamed badr email : mohabadr180@gmail.com mob. 01067157143
Assay	: MTT cytotoxicity assay
Samples	: 01 compound.
Cell lines	: ----
Ref.	: ----
Date	: 17-10-2021
Reader	: BIOLINE ELIZA READER wl 450 nm
Kit used	: SIGMA chemicals
Solvent	: DMSO

Lab Report

*Cytotoxicity results

Ser	Sample		cytotoxicity IC50 uM	SD ±
	code	M.W g/mol	A549	
1	**4a**	311.12	**28.4**	1.52
2	**4b**	345.08	**9.5**	0.51
***	**5-FU**	130.078	**15.6**	0.83

Figure S59. Cytotoxicity assay and IC_{50} of compounds **4a**, **4b** and **5-FU** against A549 cell line.

Cell culture Protocol

Cell Line cells were obtained from American Type Culture Collection , cells were cultured using DMEM (Invitrogen/Life Technologies) supplemented with 10% FBS (Hyclone,), 10 ug/ml of insulin (Sigma), and 1% penicillin-streptomycin. All of the other chemicals and reagents were from Sigma, or Invitrogen.

Plate cells (cells density 1.2 – 1.8 × 10,000 cells/well) in a volume of 100µl complete growth medium + 100 ul of the tested compound per well in a 96-well plate for 24 hours before the MTT assay .

Cell cuture protocol

1. Remove culture medium to a centrifuge tube.

2. Briefly rinse the cell layer with 0.25% (w/v) Trypsin 0.53 mM EDTA solution to remove all traces of serum which contains Trypsin inhibitor.

3. Add 2.0 to 3.0 ml of Trypsin EDTA solution to flask and observe cells under an inverted microscope until cell layer is dispersed (usually within 5 to 15 minutes).

Note: To avoid clumping do not agitate the cells by hitting or shaking the flask while waiting for the cells to detach. Cells that are difficult to detach may be placed at 37°C to facilitate dispersal.

4. Add 6.0 to 8.0 mL of complete growth medium and aspirate cells by gently pipetting.

5. Transfer the cell suspension to the centrifuge tube with the medium and cells from step 1, and centrifuge at approximately 125 xg for 5 to 10 minutes. Discard the supernatant.

6. Resuspend the cell pellet in fresh growth medium. Add appropriate aliquots of the cell suspension to new culture vessels.

7. Incubate cultures at 37°C for 24 hrs.

8-After treatment of cells with the serial concentrations of the compound to be tested incubation is carried out for 48 h at 37ºC ,then the plates are to be examined under the inverted microscope and proceed for the MTT assay

MTT – Cytotoxicity assay protocol

The MTT method of monitoring in vitro cytotoxicity is

well suited for use with multiwell plates. For best results, cells in the log phase of growth should be employed and final cell number should not exceed 106 cells/cm2. Each test should include a blank containing complete medium without cells.

1. Remove cultures from incubator into laminar flow hood or other sterile work area.

2. Reconstitute each vial of MTT [M-5655] to be used with 3 ml of medium or balanced salt solution without phenol red and serum. Add reconstituted MTT in an amount equal to 10% of the culture medium volume.

3. Return cultures to incubator for 2-4 hours depending on cell type and maximum cell density. (An incubation period of 2 hours is generally adequate but may be lengthened for low cell densities or cells with lower metabolic activity.) Incubation times should beconsistent when making comparisons.

4. After the incubation period, remove cultures from incubator and dissolve the resulting formazan crystals by adding an amount of MTT Solubilization Solution [M-8910] equal to the original culture medium volume.

5. Gentle mixing in a gyratory shaker will enhance dissolution. Occasionally, especially in dense cultures, pipetting up and down [trituration] may be required to completely dissolve the MTT formazan crystals.

6. Spectrophotometrically measure absorbance at a wavelength of 570 nm. Measure the background absorbance of multiwell plates at 690 nm and subtract from the 450 nm measurement. Tests performed in multiwell plates can be read using the appropriate type of plate reader or the contents of individual wells may be transferred to appropriate size cuvets for spectrophotometric measurement.

researcher	assay	Date	cells
Dr.Moh.Badr	MTT	17-Oct	A549

	Blank	CC	Sample No.	4a/A549				Sample No.	4b/A549			
	1	2	3	4	5	6	7	8	9	10	11	12
A	B	C	100uM	25uM	6.3uM	1.6uM	0.4uM	100uM	25uM	6.3uM	1.6uM	0.4uM
B	B	C	100uM	25uM	6.3uM	1.6uM	0.4uM	100uM	25uM	6.3uM	1.6uM	0.4uM
C	B	C	100uM	25uM	6.3uM	1.6uM	0.4uM	100uM	25uM	6.3uM	1.6uM	0.4uM

ROBONIK P2000 Eia reader
Wave length: 450 nm
Reference: 630 nm

	1	2	3	4	5	6	7	8	9	10	11	12
A	0	0.549	0.215	0.288	0.339	0.402	0.454	0.167	0.245	0.298	0.343	0.379
B	0	0.533	0.203	0.283	0.347	0.392	0.462	0.172	0.237	0.303	0.346	0.388
C	0	0.561	0.217	0.292	0.345	0.398	0.457	0.178	0.242	0.306	0.352	0.394
mean	0	0.548	0.2117	0.2877	0.3437	0.3973	0.4577	0.1723	0.241	0.3023	0.347	0.387
%		`	38.649	52.526	62.751	72.55	83.567	31.467	44.07	55.204	63.36	70.663

4a/A549

log conc.	% viability
2	38.6
1.398	52.5
0.796	62.8
0.193	72.6
-0.41	83.6

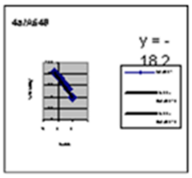

4b/A549

log conc.	% viability
2	31.47
1.3979	44.07
0.7959	55.2
0.1931	63.36
-0.409	70.66

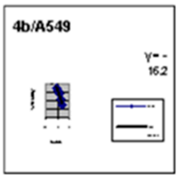

IC50= [redacted]

IC50= [redacted]

	Blank	CC	Sample No.	5-FU/A549				Sample No.				
	1	2	3	4	5	6	7	8	9	10	11	12
A	B	C	100uM	25uM	6.3uM	1.6uM	0.4uM					
B	B	C	100uM	25uM	6.3uM	1.6uM	0.4uM					
C	B	C	100uM	25uM	6.3uM	1.6uM	0.4uM					

ROBONIK P2000 Eia reader
Wave length: 450 nm
Reference: 630 nm

	1	2	3	4	5	6	7	8	9	10	11	12
A	0	0.488	0.178	0.228	0.272	0.324	0.367					
B	0	0.475	0.193	0.236	0.279	0.308	0.356					
C	0	0.505	0.184	0.234	0.265	0.319	0.358					
mean	0	0.489	0.185	0.2327	0.272	0.317	0.3603	0	0	0	0	0
% viability			37.807	47.548	55.586	64.782	73.638	0	0	0	0	0

5-FU/A549

log conc.	% viability
2	37.8
1.398	47.5
0.796	55.6
0.193	64.8
-0.41	73.6

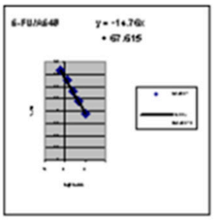

log conc.	% viability
2	0
1.3979	0
0.7959	0
0.1931	0
-0.409	0

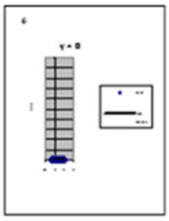

IC50= [redacted]

IC50= [redacted]

Figure S60. Raw data of cytotoxicity assay and IC_{50} of compounds **4a**, **4b** and **5-FU** against A549 cell line.

Al-Azhar University

The Regional Center for Mycology and Biotechnology

Requester Data:

Name: Dr. Moaamen Ramadan

Authority: Faculty of Pharmacy, Assuit University

Sample Data:

Fourteen samples had been submitted for elemental analysis.

Analysis Report:

Sample Code		C%	H%	N%
4b	TMO_4	62.74	4.89	4.21
4d	FTM	65.89	5.07	4.38
4e	MEX_2	66.68	5.84	4.36
6a	OB_1	74.59	5.89	3.75
6c	XFB_1	71.48	5.47	3.49
6d	$OB2_1$	69.13	5.17	3.43
6e	TCB_1	64.06	4.71	3.25
6f	TFB_1	65.98	4.78	3.31
9a	XMB_1	74.63	6.01	3.70
9b	XCB_2	69.12	5.30	3.45
9c	XFB_2	71.34	5.42	3.61
9d	TMB_1	69.03	5.18	3.47
9e	TCB_2	64.11	4.69	3.21
9f	TFB_2	66.03	4.79	3.38

INVESTIGATOR DIRECTOR

Al-Azhar University Campus - Nasr City, Cairo, Egypt.
Tel: 0202 22620373 Fax : 0202 22620373
E.mail:rcmb@azhar.edu.eg
Website: http://www.azhar.edu.eg.htm * http://www.azhar.edu.eg/pages/fungi_center.htm
Facebook : RCMB AZHAR P.O. box mail : 11751 Nasr City Cairo, Egypt.

Figure S61. elemental analysis data.
